# Characterization of *Adelphocoris suturalis* (Hemiptera: Miridae) Transcriptome from Different Developmental Stages

**DOI:** 10.1038/srep11042

**Published:** 2015-06-05

**Authors:** Caihong Tian, Wee Tek Tay, Hongqiang Feng, Ying Wang, Yongmin Hu, Guoping Li

**Affiliations:** 1 Henan Key Laboratory of Crop Pest Control, MOA Key Regional Crop Integrated Pest Management (IPM) Laboratory in Southern Part of Northern China, International Joint Research Laboratory for Crop Protection of Henan Institute of Plant Protection, Henan Academy of Agricultural Sciences, Zhengzhou 450002, China; 2 CSIRO, Clunies Ross Street, ACT 2601, Australia

## Abstract

*Adelphocoris suturalis* is one of the most serious pest insects of Bt cotton in China, however its molecular genetics, biochemistry and physiology are poorly understood. We used high throughput sequencing platform to perform *de novo* transcriptome assembly and gene expression analyses across different developmental stages (eggs, 2^nd^ and 5^th^ instar nymphs, female and male adults). We obtained 20 GB of clean data and revealed 88,614 unigenes, including 23,830 clusters and 64,784 singletons. These unigene sequences were annotated and classified by Gene Ontology, Clusters of Orthologous Groups, and Kyoto Encyclopedia of Genes and Genomes databases. A large number of differentially expressed genes were discovered through pairwise comparisons between these developmental stages. Gene expression profiles were dramatically different between life stage transitions, with some of these most differentially expressed genes being associated with sex difference, metabolism and development. Quantitative real-time PCR results confirm deep-sequencing findings based on relative expression levels of nine randomly selected genes. Furthermore, over 791,390 single nucleotide polymorphisms and 2,682 potential simple sequence repeats were identified. Our study provided comprehensive transcriptional gene expression information for *A. suturalis* that will form the basis to better understanding of development pathways, hormone biosynthesis, sex differences and wing formation in mirid bugs.

Bt cotton has been widely used to control the Old World cotton bollworm *Helicoverpa armigera* in China for nearly two decades and achieved great economic and ecological benefits^1^. However, some non-target mirid bugs species (Hemiptera: Miridae) have gradually increased their population size and acquired ecological niches on Bt cotton and other economic crops^2^,^3^. Among them, *Adelphocoris suturalis* Jakovlev (Hemiptera: Miridae) is one of the most serious pests in China’s major cotton growing regions^4^,^5^.

*A. suturalis* overwinters with diapause eggs laid in weed hosts that neighbor cotton fields^6^,^7^,^8^. In early April, overwintered eggs hatch and the first generation of nymphs survive on early-season non-target host plants from overwintering habitats^9^. Adults emerge in early May and disperse to flowering wild hosts and produce a second generation there, follow by second generation adults migrating to cotton fields in mid June, and producing two additional generations on cotton^10^. Both nymphs and adults feed on cotton flower buds and developing plant parts, causing damaged plant parts to be perforated, leading to abnormal growth, wilting or abscise^11^. Other plant hosts such as fruit trees (e.g., *Vitis vinifera* Linnaeus, *V. labrusca* L., *Ziziphus jujuba* Mill), alfalfa and vegetable crops (e.g., *Phaseolus vulgaris* L., *Daucus carota* L.) can also be impacted by damages to immature fruits and developing stems^12^. In the Yellow River cotton region, four generations of *A. suturalis* are typically recorded, while 5–6 generations are reported for the Changjiang river cotton region. The insect’s annual life cycle is characterized by the final adult generation migrating out of cotton fields and feed on non-target flowering hosts, and subsequently ovipositing diapause eggs during early autumn^13^. Currently, the use of broad-spectrum insecticides is the most preferred method for *A. suturalis* management^12^. However, prolonged dependence on insecticides can lead to the development of resistance and increasing residue build-up, and as a result, contributes to environmental pollution problems.

*A. suturalis* has been extensively studied from ecological and physiological perspectives^2^,^3^,^4^,^5^,^6^,^7^,^8^,^9^,^10^,^11^,^12^,^13^,^14^,^15^, and its metathoracic scent glands transcriptome was recently reported^16^. However, molecular regulatory mechanisms of *A. suturalis* largely remained unexplored due to the general lack of genomic resources especially relating to major transitional stages of its life cycle, which are needed to better enable the development of alternative control and management strategies of this pest. In this report, we sequenced and analyzed the transcriptomes from various developmental and adult life stages of *A. suturalis.* The assembled and annotated transcriptome sequences will assist with the identification of genes involved in *A. suturalis* development modulation and physiological aspects, help to better understand gene expression differences between developmental stages, provide the molecular basis for developing genomic markers, enable gene discovery, and functional analysis of expressed genes.

## Results

### Illumina sequencing and reads assembly

To acquire an in-depth understanding of molecular mechanisms controlling *A. suturalis* biology at various developmental life stages, cDNA was generated from eggs (clean reads accession number: SRX533456), 2^nd^ instar nymphs (clean reads accession number: SRX534296), 5^th^ instar nymphs (clean reads accession number: SRX534293), *A. suturalis* female adults (AFA) (clean reads accession number: SRX534295), and *A. suturalis* male adults (AMA) (clean reads accession number: SRX534294), followed by sequencing using the Illumina HiSeq^TM^ 2000 sequencing platform. A total of 264,212,834 clean reads were generated and assembled into 677,305 contigs from the five cDNA libraries (Table 1 and Table 2). The egg cDNA library produced the most data (55,038,084 clean reads), while the AFA cDNA library produced the least (51,295,410 clean reads). Q20 score (the average quality value) was > 97% (Table 1), and SOAPdenovo was used to map raw reads^17^. Quality checks and contig assembly were carried out using the Trinity software < http://trinityrnaseq.sourceforge.net/>^18^ after removal of adaptor sequences. The assembled reads representing these different developmental stages resulted in 88,614 unigenes and a total length of 70,479,040 base pair (bp), with a mean contig length of 795 bp (N50 = 1,398 bp), including 23,830 distinct clusters and 64,784 distinct singletons (Table 2). The unigene sequence length size distributions showed that 22,230 unigenes were over 1,000 bp. Across the entire developmental stages of *A. suturalis*, the unigene sequence length size distribution indicated that 11,993 unigenes of eggs, 11,344 unigenes of 2^nd^ instar nymphs, 10,692 unigenes of 5^th^ instar nymphs, 10,352 unigenes of AFA, and 13,223 unigenes of AMA were more than 1,000 bp (Table S1).

### Annotation of predicted proteins

**Figure 1:**
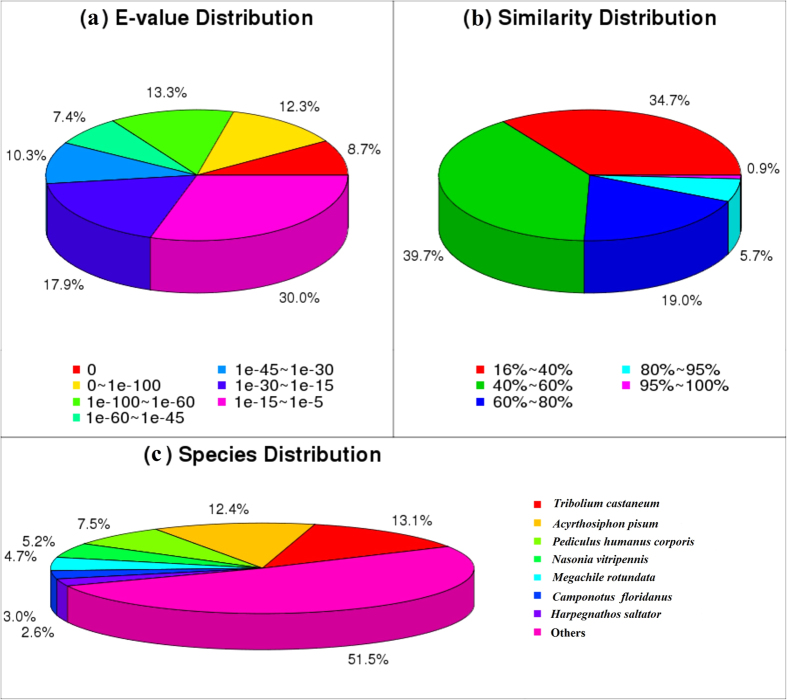
Pie-chars showing distributions from BLASTx matches of *Adelphocoris suturalis* transcriptome unigenes with respect to (**a**) E-values, (**b**) gene identity, and (**c**) insect species from which the homologous genes were matched to.

We searched all 88,614 unigene sequences using the NCBI BLASTx^19^ tool to annotate these hypothetical proteins of *A. suturalis,* and setting a cut-off E-value of 1e^−5^ as the minimum level of significance. 29,971 of total *A. suturalis* unigenes (34%) from the search were successfully annotated, with 27,392 of these (30%) returning specific matches in the non-redundant (nr) database. 11,026 of unigenes (12%) had significant matches in the nucleotide (nt) database, and 21,564 unigenes (24%) had specific matches in the Swiss-Prot database (Table 3). E-value distributions suggested that 52.0% of the assembled sequences had significant homology (<1e^−30^) to previously reported sequences when searched against the nr database, while 47.9% of homologous sequences had significant E-values that ranged between 1e^−30^ and 1e^−5^ (Fig. 1a). The similarity distributions showed that 25.6% of sequences had more than 60% similarity to sequences within the nr database, while 74.4% of matches had lower similarity that ranged from 16% to 59% (Fig. 1b). Matches to the nr database also indicated that a large number of *A. suturalis* unigenes closely matched sequences of the red flour beetle *Tribolium castaneum* (13.1%), the pea aphid *Acyrthosiphon pisum* (12.4%), the body louse *Pediculus humanus corporis* (7.5%), the parasitic wasp *Nasonia vitripennis* (5.2%), the alfalfa leafcutter bee *Megachile rotundata* (4.7%), the Florida carpenter ant *Camponotus floridanus* (3.0%), and the ponerine ant *Harpegnathos saltator* (2.6%) (Fig. 1c).

### GO, COG and KEGG Classification

**Figure 2:**
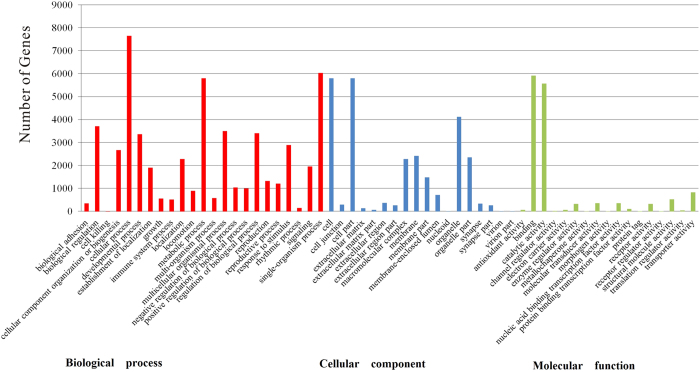
Annotations of *Adelphocoris suturalis unigenes* according to the GO categories of ‘Biological process (red)’, ‘Cellular component (blue)’ and ‘Molecular function (green)’. Subcategories within each GO category are also listed. The y-axis indicates the number of a specific category of genes in the main category.

We characterized predicted *A. suturalis* unigenes by Gene Ontology (GO), Clusters of Orthologous Groups (COG), and Kyoto Encyclopedia of Genes and Genomes (KEGG) to enable conceptualization of its transcripts into potential functional groups. A total of 12,121 unigenes were allocated to specific GO categories, 93,859 unigenes were putatively identified with GO functions, including 52,726 (56.18%) sequences at the biological process level, 26,660 (28.4%) sequences at the cellular component level, and 14,473 (15.42%) sequences at the molecular function level. 57 categories were subdivided from the main categories, and within the ‘Biological process’ GO category, 7,650 isotig sequences were assigned to ‘Cellular process’ and 6,030 to ‘Single-organism process’. Unigenes assigned to the ‘Cell’ (5,803) and ‘cell part’ (5,802) subcategories were most abundant among the ‘Cellular component’ category, and within the ‘Molecular function’ category, 5,920 isotig sequences were associated with binding and catalytic activity functions (Fig. 2).

**Figure 3:**
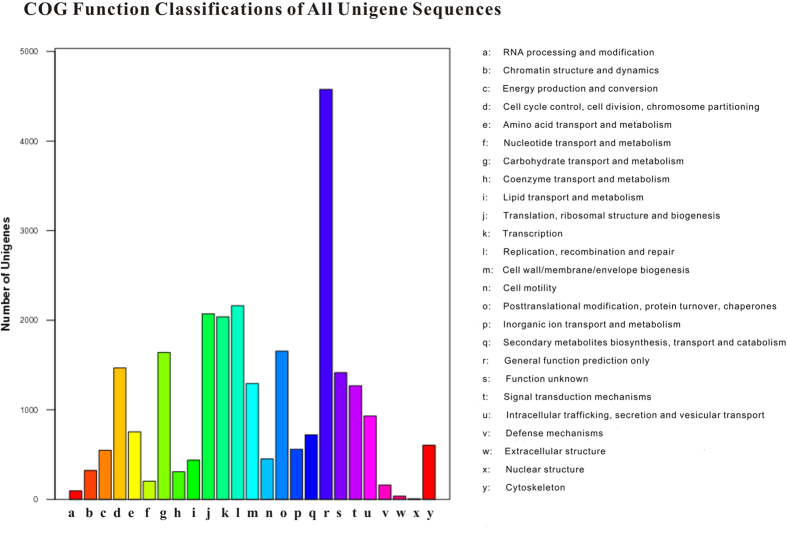
COG categories of 88,614 *Adelphocoris suturalis* unigenes. The name (including number and percentage present) for each class definition is also provided: **a**: RNA processing and modification (95, 0.37%). **b**: Chromatin structure and dynamics (323, 1.26%). **c**: Energy production and conversion (547, 2.13%). **d**: Cell cycle control, cell division, chromosome partitioning (1,467, 5.71%). **e**: Amino acid transport and metabolism (753, 2.93%). **f**: Nucleotide transport and metabolism (202, 0.79%). **g**: Carbohydrate transport and metabolism (1,639, 6.37%). **h**: Coenzyme transport and metabolism (308, 1.20%). **i**: Lipid transport and metabolism (438, 1.70%). **j**: Translation, ribosomal structure and biogenesis (2,072, 8.06%). **k**: Transcription (2,037, 7.92%). **l**: Replication, recombination and repair (2,162, 8.41%). **m**: Cell wall/membrane/envelope biogenesis (1,293, 5.03%). **n**: Cell motility (452, 1.76%). **o**: Posttranslational modification, protein turnover, chaperones (1,653, 6.43%). **p**: Inorganic ion transport and metabolism (559, 2.17%). **q**: Secondary metabolites biosynthesis, transport and catabolism (721, 2.80%). **r**: General function prediction only (4,575, 17.79%). **s**: Function unknown (1,414, 5.50%). **t**: Signal transduction mechanisms (1,268, 4.93%). **u**: Intracellular trafficking, secretion, and vesicular transport (930, 3.62%). **v**: Defense mechanisms (159, 0.62%). **w**: Extracellular structures (36, 0.14%). **x**: Nuclear structure (6, 0.02%). **y**: Cytoskeleton (604, 2.35%).

Among COG functional classifications, 10,351 functions involved in 25 COG categories were assigned to 25,713 unigenes (Fig. 3). The largest group was the ‘general function prediction’ (4,575 genes, 17.79%), followed by ‘replication, recombination and repair functions’ (2,162 genes, 8.41%), ‘translation, ribosomal structure and biogenesis’ (2,072 genes, 8.06%), ‘transcription’ (2,037 genes, 7.92%), ‘post translational modification, protein turnover, chaperones’ (1,653 genes, 6.43%), ‘carbohydrate transport and metabolism’ (1,639 genes, 6.37%), ‘cell cycle control, cell division, chromosome partitioning’ (1,467 genes, 5.71%), ‘cell membrane/envelope biogenesis’ (1,293 genes, 5.03%), and ‘signal transduction mechanisms’ (1,268 genes, 4.93%). Intermediate groups (i.e., 500–1,000 genes) as predicted by COG included those annotated as ‘intracellular trafficking, secretion, and vesicular transport’ (930 genes, 3.62%), ‘amino acid transport and metabolism’ (753 genes, 2.93%), ‘secondary metabolites biosynthesis, transport and catabolism’ (721 genes, 2.80%), ‘cytoskeleton’ (604 genes, 2.35%), ‘inorganic ion transport and metabolism’ (559 genes, 2.17%), and ‘energy production and conversion’ (547 genes, 2.13%). Genes annotated as ‘cell motility’ (452 genes, 1.76%), ‘lipid transport and metabolism’ (438 genes, 1.70%), ‘chromatin structure and dynamics’ (323 genes, 1.25%), ‘coenzyme transport and metabolism’ (308 genes, 1.20%), ‘nucleotide transport and metabolism’ (202 genes, 0.79%), ‘defense mechanisms’ (159 genes, 0.62%), ‘RNA processing and modification’ (95 genes, 0.37%), ‘extracellular structures’ (36 genes, 0.14%), and ‘nuclear structure’ (6 genes, 0.02%), represented the smallest groups (1–499 genes) as predicted by COG. There were 1,414 genes (5.50%) with unknown functions.

To better understand the biological pathways involved in *A. suturalis*, potential pathways were investigated using all 19,204 sequences identified in the KEGG Orthology (KO) annotation of the mirid bug, with sequences mapped to the reference authoritative pathways in the KEGG database. As a result, a total of 258 KEGG pathways were identified, with 2,971 unigenes (15.47%) being involved in metabolic pathways. These pathways played a dominant role in pathways associated with RNA transport, olfactory transduction, insect hormone biosynthesis, and ubiquitin mediated proteolysis, amongst others (Table S2).

### CDS prediction of the *A. suturalis* transcriptome

**Figure 4:**
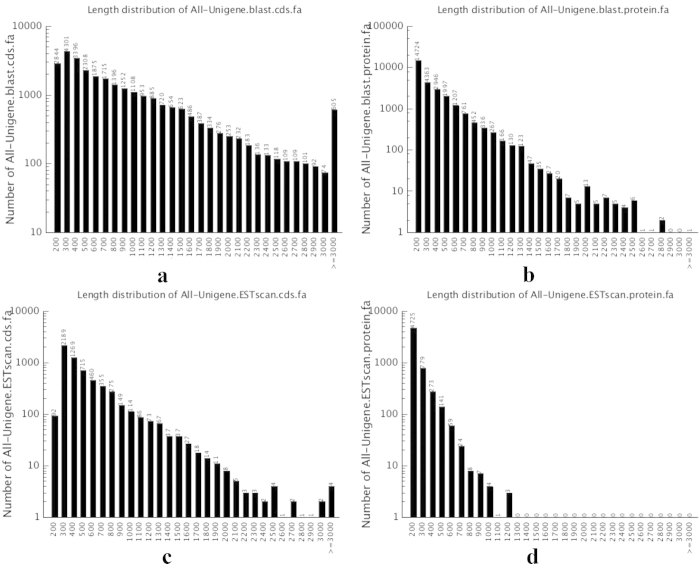
Summary of *Adelphocoris suturalis* transcriptome coding sequence predictions for: (**a**) Length distribution of CDS predicted from BLASTx (E-value < 0.00001) to protein databases in the priority order of nr, Swiss-Prot, KEGG and COG; (**b**) Length distribution of proteins predicted by BLASTx results; (**c**) Length distribution of CDS predicted by ESTScan, and **(d**) Length distribution of proteins predicted by ESTScan.

CDS were extracted from BLASTx results with 88,614 unigene sequences being translated into protein sequences. We annotated 33,682 CDS through such procedures that included 27,658 CDSs being identified from BLASTx results, with 6,024 CDS remained unidentified. To better assign gene names and annotations, CDS and predicted proteins, all assembled unigenes were first searched against the NCBI nr and Swiss-Prot databases using BLASTx. As a result, 73.11% (64,782 of the total 88,614 unigene sequences) of the predicted protein unigenes showed significant similarity to known proteins of various insects that included *Lygus lineolaris* (Hemiptera: Miridae)*, Papilio polytes* (Lepidoptera: Papilionidae), *A. pisum* (Homoptera: Aphididae) and *T. castaneum* (Coleoptera: Tenebrionidae). The distribution of unigene lengths with homologous matches was: 200-500 nucleotides (bp) (n = 12,849; 14.50%), 600–1,000 bp (n = 7,346; 8.29%), 1,100–3,000 bp (n = 6,858; 7.74%), and >3,000 bp (n = 605; 0.69%) (Fig. 4a). Coding regions of unigenes were also translated into amino acid (aa) sequences, where 14,724 (16.62%) unigenes coded for polypeptides of approximately 200 aa long, 10,513 (11.86%) for polypeptide lengths that ranged from 300 to 600 aa, 2,317 (2.61%) for lengths of between 700 and 1,500 aa, and 104 unigenes (0.12%) for polypeptide lengths of between 1,600 and 3,000 aa (Fig. 4b). These included unigenes coding for a hypothetical protein (unigene CL171; 3,716 aa), for a protein similar to fatty acid synthase (unigene CL303; 2,402 aa), for fatty acid synthase-like isoform 2 (unigene CL303; 2,256 aa), and voltage-sensitive sodium channel alpha-subunit (unigene CL46; 2,009 aa).

The remaining 6,024 CDS with no specific matches to the above databases were predicted by ESTScan. Potential protein unigenes with lengths between 200 to 500 bp accounted for 70.80% (n = 4,265). Other categories of nucleotide lengths included: (i) 600–1,000 bp (1,353; 22.46%), (ii) 1,100–3,000 bp (402; 6.67%), and (iii) >3,000 bp (4; 0.07%) (Fig. 4c). 78.44% (n = 4, 725) of these unigenes were translated into polypeptide sequences of approximately 200 bp long from the putative protein unigenes by ESTScan, while 17.46% (n = 1,052), 3.32% (n = 200), 0.71% (n = 43) and 0.07% (n = 4) were translated into polypeptide sequences of between 300–400 bp, 500–600 bp, 700–1,000 bp, and 1,100–1,200 bp, respectively (Fig. 4d).

### Simple sequence repeats (SSRs) identification and primers development

**Figure 5:**
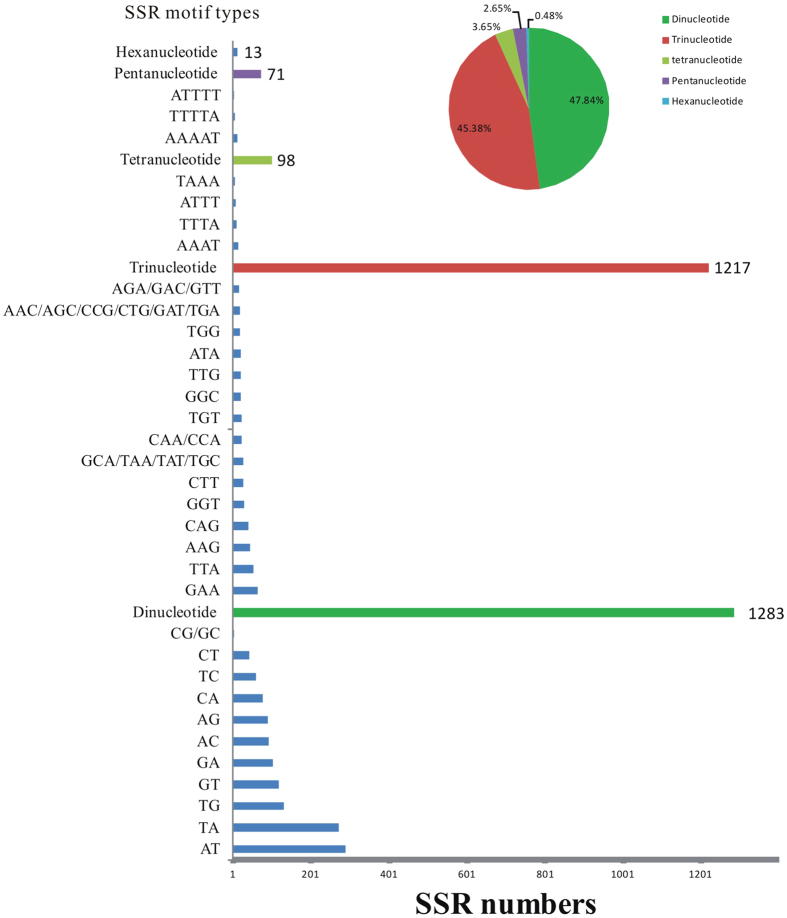
Simple sequence repeats (SSRs) characterization from *Adelphocoris suturalis* transcriptomes. The pie chart indicates the percentage of the nucleotide. The column chart represents the SSR number (x-axis) and SSR motif types (y-axis).

We screened the *A. suturalis* unigene dataset to determine the nature and frequency of SSRs within our reduced representation of its genome. Among the total of 3,293 unigenes mapped, more than 3,200 pairs of potential SSR primers were designed, although primer efficacies were not tested with respect to *A. suturalis* population and evolutionary genetics, nor their relationships to microsatellite DNA family^20^, transposable elements^21^, and implications to genome organization and rates of evolution^22^. By searching for di-, tri-, tetra-, penta- and hexa-nucleotide repeats, we obtained a total of 2,682 SSRs, of which the majority of SSRs have less than 24 repeat units, with 5–8 repeat units being the most common categories. Among all SSRs identified, dinucleotide repeats (47.84%) represented the most abundant microsatellite repeat units, followed by trinucleotide (45.38%), tetranucleotide (3.65%), pentanucleotide (2.65%), and hexanucleotide (0.48%) repeats, respectively (Fig. 5).

### SNPs identification

791,390 potential SNPs were identified using the program PolyBayes (Table 4), of which 470,677 (59.47%) were transitions (A-G, C-T). Within individual life-stages, the greatest amount of transitional base changes (63,306 A-G; 62,494 C-T) were from egg samples, while the AMA samples recorded the least (39,225 A-G; 38,365 C-T). 40.53% of SNPs were transversions, with 14.78% being A-T transversions, followed by G-T (9.83%), A-C (9.77%), and C-G (6.14%) transversions. From the entire dataset, the average SNP frequency is 0.011 SNP/nt and the average transition and transversion frequency is 0.0067 transition/nt and 0.0046 transversion/nt, respectively. The complete list of SNPs identified across the selected developmental stages (i.e., eggs; 2^nd^ and 5^th^ nymph stages; AMA; and AFA) is provided in Table S3.

### Comparison of gene expression profile among *A. suturalis* developmental stages

**Figure 6:**
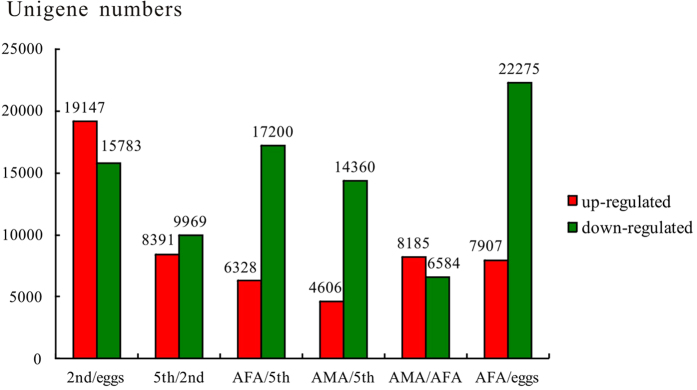
Numbers of unigenes between selected *Adelphocoris suturalis* developmental stage comparisons. Up-regulated (red) and down-regulated (green) unigenes were quantified using the tag-based digital gene expression (IDEG6) tool. Details of results from six comparisons, representing major life-stage transitional phases (i.e., eggs/2nd; 2nd/5th; 5th/AFA and 5th/AMA), sex differences (AFA/AMA), and reproductive stage (AFA/eggs), are provided in the main text. Note ‘2nd’, ‘5th’, AMA, and AFA are 2^nd^ instar nymph stage, 5^th^ instar nymph stage, adult male and adult female life stages, respectively.

To confirm the relative expression levels of differentially expressed genes across the major developmental stages, we calculated the number of clean tags for every gene, and gene expressed differentially were identified using the IDEG6 tool^23^ between every two *A. suturalis* life-stages that represented selected transitional developmental stages (eggs and 2^nd^ instar nymphs, 2^nd^ and 5^th^ instar nymphs, 5^th^ instar nymphs and adult stages (i.e., AFA, AMA), the reproductive stage (i.e., AFA and eggs), and sex differences (i.e., AMA and AFA)) (Fig. 6). Furthermore, we also analyzed the change of gene expression between different developmental stages by GO and KEGG pathway enrichment analysis.

In the comparison between eggs and 2^nd^ instar nymphs, the expression profiles of 34,930 genes had changed. There were 19,147 unigenes that were up-regulated in the 2^nd^ instar nymph library and 15,783 unigenes down-regulated in the egg library (Fig. 6). Among the top ten up-regulated genes, significant matches included the gene homologous to one that encodes the odorant-binding protein 2 in the related species *A. lineolatus*; a cysteine proteinase gene in *Dictyostelium fasciculatum*, three cuticular protein genes (*Bombyx mori* putative cuticle protein; *C. floridanus* cuticle protein 21, and *C. floridanus* cuticle protein 19.8), and three predicted functional genes (*T. castaneum* hypothetical protein TcasGA2_TC007306; *Columba livia* putative serine/threonine-protein kinase kinX, and *Macaca mulatta* type I inositol-1,4,5-trisphosphate 5-phosphatase-like). The top ten down-regulated genes in eggs libraries included predicted functional genes; an uncharacterized protein gene LOC101240627 (*Hydra magnipapillata*); Proclotting enzyme gene (*C. floridanus*), GK14571 (*Drosophila willistoni*), Toutatis gene (*T. castaneum*), one Longitudinals lacking protein isoforms A/B/D/L (*Acromyrmex echinatior*), the hypothetical protein TcasGA2_TC006306 gene (*T. castaneum*)); the GF17401 gene (*D. ananassae*), and one transport protein Sec24C (*H. saltator*) (Table S4).

Differentially expressed genes between eggs and 2^nd^ instar nymphs were characterized into three groups from the GO classification: (i) Biological process, (ii) Cellular component, and (iii) Molecular function. The results showed 4,118 biological processes-related genes were predominantly concentrated in the ‘Cellular process’ subcategory, and 3,329 genes involved in the ‘single-organism process’ subcategory. A total of 3,067 genes were associated with the ‘cell/cell part’ subcategory within the ‘Cellular component’ category, while 3,038 genes were involved in ‘binding’ within the ‘Molecular function’ category (Table S5a). In the pathway analysis (Table S5b), among all 19,204 unigenes involved in 257 pathways, the most differentially expressed genes from the eggs and 2^nd^ instar nymphs comparison were involved in ‘metabolic pathways’ (1,302), ‘regulation of actin cytoskeleton’ (444), ‘RNA transport’ (394) and ‘focal adhesion’ (376).

Between 2^nd^ and 5^th^ instar nymph libraries, 18,360 genes showed significant expression changes from the comparative analysis, with 8,391 genes up-regulated in the 5^th^ instar nymph library and 9,969 down-regulated genes in 2^nd^ instar nymph library (Fig. 6). Among the top ten differentially up-regulated and down-regulated expressed genes, 4 showed defined functions, i.e., a cytochrome c oxidase subunit 6B1 gene (*A. echinatior*) in up-regulated genes group; a 48 kDa subunit-like protein (*M. rotundata*), a Translocon-associated protein subunit delta precursor gene, and a sugar transporter (*Culex quinquefasciatus*) in the down-regulated genes group. Two of ten up-regulated genes have predicted functions, i.e., TcasGA2_TC005836 protein gene (*T. castaneum*), and CBG23181 protein gene (*Caenorhabditis briggsae*). Among ten most down-regulated genes, five genes have predicted functions, i.e., putative transporter SVOPL-like gene (*Bombus impatiens*), dolichyl-diphosphooligosaccharide--protein glycosyltransferase 48 kDa subunit-like (*M. rotundata*), hypothetical protein SINV_03497 (*Solenopsis invicta*), RNA-binding protein (*Triatoma infestans*), and regulator of G-protein signaling 12-like (*A. pisum*) (Table S4). Gene sets in this library were predicted by GO analysis (Table S6a) to have high accordance with genes associated with ‘cellular process’ (2,024), ‘single-organism process’ (1,710), ‘metabolic process’ (1,437), and ‘biological regulation’ (1,045) within the ‘Cellular component’ category. Among the ‘Molecular function’ category, 16 subcategories were identified with the highest two subcategories being ‘catalytic activity’ (1,489) and ‘binding’ (1,472). Pathway analysis of 5,241 gene sets identified those involved in ‘metabolic pathways’ (747), ‘regulation of actin cytoskeleton’ (230), ‘RNA transport’ (177), and ‘mRNA surveillance pathway’ (125) (Table S6b).

The comparative analysis between 5^th^ instar and AFA libraries revealed 23,528 genes with significant expression profile changes (Fig. 6). Two of the ten most up-regulated genes in the 5^th^ instar library have homologies to *D. ananassae* GF17401 and GH24370 genes. Three genes have predicted functions, i.e., cathepsin B-like of *Oryzias latipes* and cathepsin L-like of *Strongylocentrotus purpuratus*, and opacity protein and related surface antigens of *Ixodes scapularis*. Three genes have defined functions: RP45 gene of *Rhodnius prolixus*, CBN-CPR-4 protein gene of *C. brenneri*, reproduction-associated glycoprotein 7 of *Odocoileus virginianus,* and two unannotated genes. Among the ten most down-regulated genes, one matched the reverse transcriptase gene of *Aedes aegypti* and one matched *D. melanogaster*’s sallimus, isoform Z. Three genes have predicted functions, i.e., general transcription factor II-I repeat domain-containing protein 2A-like (*Xenopus (Silurana) tropicalis*), RNA-directed DNA polymerase from mobile element jockey-like (*Metaseiulus occidentalis*), and high affinity cAMP-specific and IBMX-insensitive phosphodiesterase (*A. pisum*) (Table S4). GO analysis (Table S7a) revealed that gene sets in this library showed high accordance with genes relating to ‘Cellular process’ (2,173), ‘single-organism process’ (1,772), ‘metabolic process’ (1,664), and ‘biological regulation’ (1,010) within the ‘Biological process’ category. Among the ‘Cellular component’ category, ‘cell/cell part’ (1,623), ‘organelle’ (1,215) and ‘organelle part’ (719) were the three subcategories with the highest number of gene sets. Among the ‘Molecular function’ category, 1,748 genes acted as catalytic activity, and 1,607 genes were involved in binding. Among 256 pathways identified (Table S7b), gene sets were correlated to ‘metabolic pathway’ (885), ‘regulation of actin cytoskeleton’ (302), ‘RNA transport’ (243), ‘cell cycle’ (131), ‘RNA polymerase’ (90), and ‘RNA degradation’ (58).

In the comparison of 5^th^ instar and AMA, 18,966 genes had significant expression profiles, with 4,606 up-regulated in AMA and 14,360 down-regulated genes identified in 5^th^ libraries (Fig. 6). No homology to previously characterized genes was identified among the ten most differentially up-regulated genes. While among the ten most differentially up-regulated genes, three genes showed defined functions, i.e., exuperantia (*T. castaneum*), ankyrin repeat-containing protein (*P. h. corporis*), Dynein light chain 1 (*D. melanogaster*) and two hypothetical protein genes (CAPTEDRAFT_141578 and LOC100160904; *A. pisum*) (Table S4). GO analysis (Table S8a) showed that five of the largest gene sets in this library was related to ‘Cellular process’ (1,091), ‘Metabolic process’ (890), ‘Single-organism process’ (881), and ‘Multicellular organismal process’ (523) within the ‘Biological process’ category. Among the ‘Cellular component’ category, of the 18 subcategories identified, 797 and 583 genes were related to the ‘cell/cell part’ and the ‘Organelle’ subcategories. Among the ‘Molecular function’ category, the top three subcategories were ‘catalytic activity’ (868), ‘binding’ (833), and ‘structural molecular activity’ (201) from a total of 14 subcategories identified. The pathway analysis identified gene sets associated with ‘metabolic pathway’ (524), ‘ribosome’ (121), ‘protein digestion and absorption’ (119), and ‘RNA transport’ (109) amongst a total of 252 pathways (Table S8b).

In the comparison between AFA and AMA libraries, the expression profiles of 14,769 unigenes had changed. Among these genes, 8,185 unigenes were up-regulated in AFA and 6,584 unigenes were down-regulated in AMA (Fig. 6). Two functional defined up-regulated genes were predicted to encode catalase-like protein of *A. pisum* and masticatory epithelia keratin 2p protein of *Canis lupus familiaris*. Among these down-regulated genes, four of them have predicted functions, i.e., TcasGA2_TC009637 (*T. castaneum*), LOC100165704 (*A. pisum*), U4/U6 small nuclear ribonucleoprotein Prp31-like protein (*B. impatiens*) and LOC100576321 (*Apis mellifera*). Three of ten down-regulated genes have defined/predicted functions, i.e., zinc finger protein RTS2 (*C. floridanus*), supporter of activation of yellow protein (*C. floridanus*), and AGAP001145-PA (*Anopheles gambiae*) (Table S4). Our analysis showed that gene sets in this library have high accordance with genes related to ‘cellular process’ (2,027), ‘single-organism process’ (1,645), ‘metabolic process’ (1,472), and ‘biological regulation’ (971) within the ‘Biological process’ category. Among the ‘Cellular component’ category, genes associated with ‘cell/cell part’ (1,502 genes), and ‘organelle’ (1,120) were most abundant (Table S9a). Among the ‘Molecular function’ category, 1,568 genes were associated with catalytic activity, and 1,459 genes were involved in binding. Among the 251 pathways identified in the KEGG pathway analysis, most of these gene sets were correlated to ‘metabolic pathway’ (701), ‘regulation of actin cytoskeleton’ (245), ‘RNA transport’ (215), ‘focal adhesion’ (174), ‘lysosome’ (153), amongst others (Table S9b).

The comparative analysis between the AFA and eggs libraries revealed expressional changes in 30,182 genes, with a total of 7,907 and 22,275 genes being found to be up- and down-regulated in the AFA library, respectively (Fig. 6). Seven of the ten most up-regulated genes have defined/predicted functions, i.e., cathepsin-L (*L. lineolaris*), GF17401 (*D. ananassae*), CG8483 (*D. melanogaster*), two RP45 genes (*Rhodnius prolixus*) and two vitellogenin genes (*Trigonotylus caelestialium*). Six of the ten most down-regulated genes have predicted functions, i.e., hypothetical protein LOC100115461 isoform 1 (*N. vitripennis*), ubiquitin carboxyl-terminal hydrolase 5-like (*B. impatiens*), eukaryotic translation initiation factor 3 subunit B-like isoform 1 (*B. impatiens*), ABC transporter G family member 20-like isoform 1 (*A*. *pisum*), TcasGA2_TC000880 (*T. castaneum*) and EEB14277.1 (*P. h. corporis*) (Table S4). Gene sets identified in GO analyses were correlated to the ‘Biological process’ category of ‘cellular process’ (4,014), ‘single-organism process’ (3,122), ‘metabolic process’ (3,037), and 20 other subcategories of ‘biological process’; a total of 16 subcategories of the ‘Cellular component’ category that included ‘cell/cell part’ (3,021), ‘organelle’ (2,224), ‘organelle part’ (1,337), ‘macromolecular complex’ (1,233); and 17 subcategories of ‘Molecular function’ that included ‘binding’ (2,980), ‘catalytic activity’ (2,817), and ‘transporter activity’ (342) (Table S10a). The gene sets of pathways showed that 1,244 unigenes were involved in metabolic pathways, 400 unigenes were involved in regulation of actin cytoskeleton and 256 other pathways (Table S10b).

### Quantitative Real-time PCR (qRT-PCR) analysis

**Figure 7:**
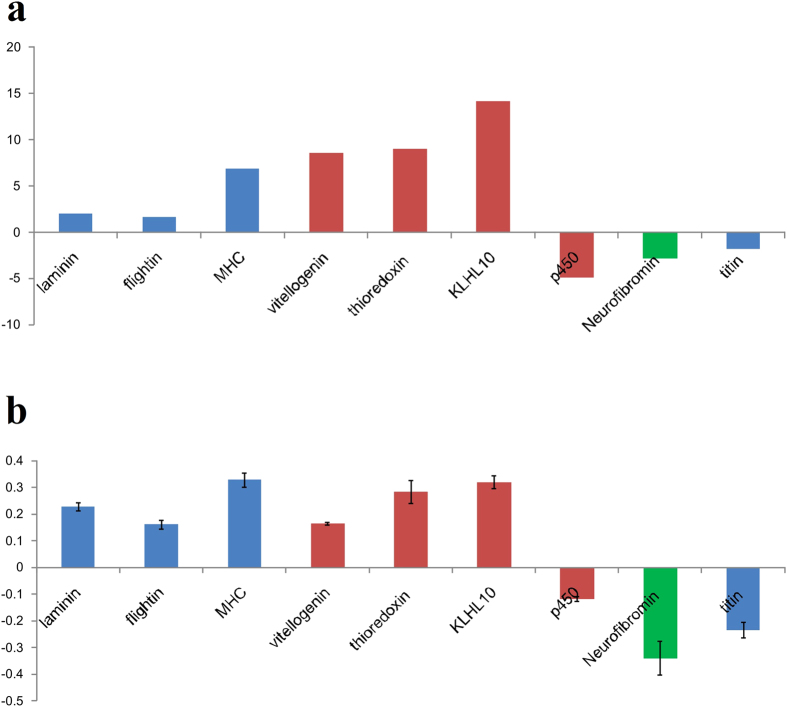
Sex-, hormone-, and wing-specific genes in *Adelphocoris suturalis*. Blue bars represent genes related to wing muscle architecture, red bars for genes associated with sex-differences, and the green bar represents hormone biosynthesis-related genes. (**a**) The fold changes for genes from transcriptome sequence analysis between AMA and AFA comparison. (**b**) The relative expression levels (Error bars represent standard deviation. of each gene by qRT-PCR in AMA compared with AFA.

To better validate the sequencing data, various genes related to wing formation (flightin, titin, myosin heavy chain (MHC), laminin), hormone biosynthesis (neurofibromin), sex difference (vitellogenin, thioredoxin, KLHL10 (Kelch-like protein 10), and detoxification (Cytochrome P450) were chosen randomly and quantified by the qRT-PCR method. Results from the qRT-PCR experiment supported those obtained from transcriptome analysis and demonstrated similar tendency in up- or down-regulation of *A. suturalis* genes (Fig. 7). From analysis of deep sequencing data, some genes related to wing dimorphism had higher copy numbers in the AMA library when compared with the AFA library, such as flightin, MHC and laminin. In the AMA cDNA library (Fig. 7a), among the 45 copies of flightin genes and 34 copies of KLHL10 genes as identified in comparison with the AFA library, our chosen single flightin gene and three KLHL10 genes showed up-regulation that concurred with our differential gene expression (DGE) analysis. Down-regulation of the titin gene as determined from DGE analysis (Fig. 7a) was also validated by qRT-PCR (Fig. 7b).

## Discussion

In this study, we report on transcriptomic analyses derived from targeted *A. suturalis* life developmental stages, thereby significantly increased molecular resources available for this arthropod pest of Bt cotton and food crops, and provides a framework for understanding changes in gene expression during development. This transcriptional information not only serves as a valuable resource to better understand underlying biological and physiological mechanisms governing *A. suturalis* life cycle, but may also lead to the identification of novel targets for bio-rationally designed strategies for the control of this insect pest.

Analysis from the RNA sequence data, amongst the generated 88,614 unigenes and predicted against the NCBI nr protein database, 29,971 sequences of the *A. suturalis* transcriptome were annotated and showed specific Swiss-Prot matches. Considering the above results, the lower-bound of genes for the *A. suturalis* transcriptome should be more than 88,614. In fact, many sequences assembled did not match significantly to DNA/protein databases due to their general short sequence length, or that they represented significantly different genes. For example, 13.1% of genes in the red flour beetle *T. castaneum*, the closest relative of *A. suturalis* with a sequenced and annotated genome, showed no homology to other metazoan genes^24^. Although lacking the full genome information of *A. suturalis*, our collection of unique transcripts for different developmental stages possibly represents significant proportion of functional genes from this insect.

An interesting finding from this study is that identification of many differentially expressed genes was achieved through comparative transcriptomic analyses across the pest insect’s developmental stages, thereby greatly enriching current knowledge of *A. suturalis* gene expression profiles. This will contribute to future research relating to molecular characterization of olfactory systems, chemical targets, developmental mechanisms and sex difference of this and other mirid plant bugs. During the early developmental stage of *A. suturalis,* most of the differentially expressed genes were up-regulated in the egg stage when compared with the 2^nd^ instar nymph stage. In contrast, most of these genes were found to be down-regulated in the AFA and eggs life stages comparison. When comparing between different developmental libraries (e.g., eggs and 2^nd^ instar nymphs; AFA and eggs), a large number of genes showed specific life stage-related expression profiles (e.g., odorant-binding protein, cuticular protein, cysteine proteinase, vitellogenin) that were likely involved in developmental differentiations.

From the comparison between AMA and AFA libraries, 8,185 up-regulated genes in the AFA library and 6,584 down-regulated genes in the AMA library were identified. 23 genes encoding catalase-like proteins were found to be up-regulated, while various genes such as the U4/U6 small nuclear ribonucleoprotein Prp31-like protein gene and the zinc finger protein RTS2 gene supporter of activation of yellow protein gene were down-regulated. Results obtained for the gene set enrichments in the study that compared AMA and AFA suggested that *A. suturalis* female adults likely to possess more active transcriptional and translational processes than did adult males, a phenomenon also found in the brown planthopper *Nilaparvata lugens* (Stål)^25^. Additionally, 1,351 sex biased genes (e.g. transformer-2 (tra-2) gene, odorant binding protein 6, cuticular protein 58) were investigated by transcriptomic analysis in the whitefly *Bemisia tabaci* (Hemiptera: Aleyrodidae)^26^, many of these genes were also identified and characterized in our transcriptome data, thereby further enriching understanding of sex differentiation in non-model pest organisms. Our transcriptome data also indicated that some *A. suturalis* genes were likely to have multiple roles, such as to both be associated with sex difference and participated in mechanisms related to cellular components, such as the masticatory epithelia keratin 2p protein, which was also involved in mechanisms associated with cytochrome P450 enzyme precursors. Interestingly, in the related *L. hesperus* (Hemiptera: Miridae), homologous protein domains were also found in transcriptome analysis of adults^27^. Our study showed that 191 cytochrome P450 genes in nymphs and 75 in adults had significantly high expression levels; almost ten times higher than the 28 cytochrome P450 genes that showed high relative expression level in pre-adults and adults stages of the hemipteran bug *Halyomorpha halys*^28^, and 65 times more than the transcriptome analysis of cytochrome P450 genes in the bed bug *Cimex lectularius*^29^. Combining this and previous studies will significantly contribute to understanding the functional roles of cytochrome P450 gene family in hemipteran insects.

In the hemipteran insect *Diaphorina citri*, 13 juvenile hormone genes were previously identified by transcriptome sequencing^30^. In this study, however, our transcriptomic data have identified only three hormone related neurofibromin genes, of which we validated the gene expression profiles of only one of these three genes in both AFA and AMA. Other highly expressed genes in *A. suturalis* adult life stage (e.g., masticatory epithelia keratin formation, zinc finger protein RTS2, supporter of activation of yellow protein) were identified which were potentially relevant to various key life developmental processes (e.g., morphogenesis, sex difference). Validating the roles of these genes in morphogenesis or sex differences will be necessary to confirm and characterize gene functions, and may offer novel gene-targeted pest management strategies for *A. suturalis*. We have validated expression profiles of selected groups of genes in this study based on qRT-PCR but have not validated their gene functional profiles. Our qRT-PCR results, although limited due to the low number of genes being tested, nevertheless offered support for the fold change as detected by Illumina sequencing. Furthermore, a total of 2,368 genes were found to show differential expression profiles across the whole life cycle of this insect, and these included candidate genes that underpinned homeostasis functionalities or feeding behavior (e.g., heat shock proteins, calcium-binding proteins, salivary secreted proteins) based on DGE analyses, and will require future empirical expression profile studies and gene characterization, with novel molecular gene-knockdown techniques (e.g., the CRSPR method^31^ or miRNA technique^32^) offering promising insights to characterizing gene functions in this insect pest.

This study has generated much needed genetic and genomic resources for the development of targeted control of *A. suturalis*. We have also identified 2,682 microsatellite DNA/simple sequence repeat units and 791,390 putative SNPs from different life stages of *A. suturalis*. We showed that the most common SSR types in the reduced genome of *A. suturalis* being dinucleotide repeats (i.e., GC/TG/AT), while transition SNPs far out numbered transversion SNPs across all life stages studied. As with other biological systems^33^,^34^,^35^,^36^, these potential molecular markers identified in this study will serve as useful tools to facilitate future construction of genetic maps, infer gene flow, identify genetic signatures of selection, and infer parentage studies in this emergence pest of Bt cotton and food crops in China.

## Materials and Methods

### Insect materials and rearing conditions

*A. suturalis* adults (male and female) were originally collected from a Bt cotton field at the Henan Research and Experiment Base for Modern Agriculture (35°0'13.2372“N, 113°42'28.8792“E) of Henan Academy of Agricultural Sciences (HAAS), Yuanyang, Henan province, China. Eggs, nymphs and adults were reared on green beans at 26 ± 1 °C, 70 ± 10% relative humidity, and a photoperiod cycle of 14 hr light/10 hr dark according to Feng *et al.*^8^. All insects of different life stages collected in this study originated from a single *A. suturalis* (monandrous) mating pair. Due to the limitation in female lifetime egg-laying capacity when reared with artificial diet (range 35–172 eggs/generation^37^), we obtained sufficient eggs, 2^nd^ and 5^th^ instar nymphs, male and female adult individuals through a second-generation population-wide mating between siblings.

### Samples collection and RNA isolation

Eggs were collected with damp filter papers within 24 h of oviposition^8^, 350 eggs were used in RNA extraction. The 2^nd^ and 5^th^ instar nymphs were distinguished based on developmental time since emergence from eggs. AFA individuals were identified by the black line in the abdomen of female adults. Male and female adults were collected four days after emergence from final (5^th^) nymph stage. 100 2^nd^ instar nymphs and 70 5^th^ instar nymphs, and 50 individuals of each female and male adults were collected into a glass tube using an aspirator. RNA isolation from all samples was performed as described in Dong *et al.*^38^, and the integrity of all RNA samples was ascertained using a 2100 Bioanalyzer (Agilent Technologies) with a minimum RNA integration value of 6. Total RNA was isolated from eggs (OD260/280 = 1.926), 2^nd^ instar nymphs (OD260/280 = 2.017), 5^th^ instar nymphs (5th) (OD260/280 = 2.024), female (AFA) (OD260/280 = 2.115) and male (AMA) adults (OD260/280 = 2.022) by the SV Total RNA Isolation System (Promega, USA).

### cDNA library construction and Illumina sequencing

To acquire the entire gene expression information, Illumina sequencing was performed for all five *A. suturalis* cDNA libraries as described in Xie *et al.*^39^ that represented the various developmental stages. All cDNA libraries were generated according to the protocol as supplied by Illumina (Illumina Inc., San Diego, CA, USA). Briefly, 20 μg of pooled total RNA samples of individual *A. suturalis* developmental stages were digested by DNase I (Sigma, USA), and mRNA purified by oligo(dT) magnetic beads and fragmented into fragments of ~100–400 bp. The cDNA was synthetized by reverse transcriptase (Invitrogen, Carlsbad, CA, USA) using random hexamer-primers with the mRNA fragments as templates. Agilent 2100 Bioanaylzer and ABI StepOnePlus Real-Time PCR System were used to quantify and qualify all libraries. We selected five cDNA libraries with 200 bp insert size for sequencing by the Illumina HiSeq2000^TM^ (Illumina Inc., San Diego, CA, USA).

### Bioinformatics analysis of sequencing results

Short sequence reads were assembled for individual samples from the targeted developmental stages of *A. suturalis* using the Trinity software. Unigenes from each of developmental stages were combined to create a lifetime unigene database of *A. suturalis* by the BLAST-Like Alignment Tool < http://genome.ucsc.edu/cgi-bin/hgBlat>. All raw sequence data has been uploaded to the NCBI Sequence Read Archive (SRA; accession number SRP041523).

Unigenes longer than 350 bp were aligned against the NCBI nucleotide database (non-redundant (nr)/non-redundant nucleotide (nt)), Swiss-Prot, COG, KEGG and TrEMBL databases. Gene ontology (GO) annotations of unigenes were achieved by the Blast2GO software < http://www.geneontology.org>. For coding sequences (CDS) annotation, BLASTx alignments were carried out between unigenes against nr, KEGG, Swiss-Prot and COG protein databases, and the transcriptional directions and coding frame of unigenes were predicted from BLASTx results. Unigene CDS with no specific BLASTx matches were predicted by ESTScan^40^. All the above searches were carried out with a cut-off e-value of <10^−5^.

### Identification of simple sequence repeats (SSRs) and single nucleotide polymorphisms (SNPs)

To assess the assembled unigene quality and to identify potential new molecular markers, potential SSR markers were identified among all unigenes using the MISA tool < http://pgrc.ipk-gatersleben.de/misa/> for detecting SSR sequences^41^ and possible PCR primers were designed using PRIMER v.3^42^. SNPs within unigenes were evaluated using SOAP2 and SOAPsnp^43^ across the various *A. suturalis* developmental stages (i.e., eggs, 2^nd^ instar nymphs, 5^th^ instar nymphs, AFA, and AMA), using all unigenes as reference for SNPs discovery. All common and unique SNP sites among these samples were identified as previously described^36^.

### Differential gene expression analyses

Fragments Per Kilobase of transcripts per Million mapped reads (FPKM) method^44^ was used to obtain the unigene expression levels of different developmental stages. Base on FPKM values, differentially expressed genes were identified using the IDEG6 software < http://telethon.bio.unipd.it/bioinfo/IDEG6/>^23^. Gene expression variations were analyzed for specific life-stages comparisons that included the following three categories: (i) transitional developmental stages (i.e., eggs and 2^nd^ instar nymphs (eggs and 2^nd^), 2^nd^ instar nymphs and 5^th^ instar nymphs (2^nd^ and 5^th^), 5^th^ instar nymphs and female adults (5^th^ and AFA), 5^th^ instar nymphs and male adults (5^th^ and AMA)); (ii) sex differences (i.e., female adults and male adults (AFA and AMA)); and (iii) reproductive stage (i.e., female adults and eggs (AFA and eggs)).

### Quantitative Real-time PCR (qRT-PCR) validation

A total of 50 male and 50 female adults were collected four days after emergence from the final (i.e., 5^th^) nymph stage, and their total RNA purified for cDNA libraries preparation as described above. For generating the first-strand cDNA, 2 μg total RNA of female and male *A. suturalis* adults was reverse transcribed in a 20 μl reaction volume using the PrimeScript RT reagent Kit with gDNA Eraser (TaKaRa). To confirm single amplicon product for qRT-PCR and bio-specificity for *A. suturalis*, gradient PCR was performed for all primer pairs and optimized for a 60 °C annealing temperature for all primer pairs. PCR amplification products were run on 1.5% 1xTAE agarose gel electrophoresis and Sanger sequenced (Biological Engineering (Shanghai) Co., Ltd.).

Quantitative Real-time PCR was performed using SYBR Premix Ex Taq™ (Tli RNase H Plus) (TaKaRa) according to the manufacturer’s protocol. In the StepOnePlus™ Real-Time PCR system (Applied Biosystems) with the following PCR-cycling conditions: 95 °C for 10 s, 40 cycles of 95 °C for 30 s, 60 °C for 30 s with the concentration at 200 nM of each primer pair. The mean threshold cycle (Ct) was measured by three replicates of each gene, to test sample contamination and dimer formation, nuclease free water as template was included in all qRT-PCR experiments as the negative control. The 2^−ΔΔ Ct^ method^45^ was used to calculate individual gene’s relative expression levels. The 18 S rRNA gene (unigene 62750) was chosen as the endogenous reference gene in the qRT-PCR experiment as previously demonstrated^25^,^46^,^47^, and was expressed at the same level in the AMA (FPKM: 3.7717) and AFA (FPKM: 3.336) in FPKM analysis. All primers pairs for qRT-PCR are listed in Table S11.

## Additional Information

**How to cite this article**: Tian, C. *et al.* Characterization of *Adelphocoris suturalis* (Hemiptera: Miridae) Transcriptome from Different Developmental Stages. *Sci. Rep.* **5**, 11042; doi: 10.1038/srep11042 (2015).

## Supplementary Material

Supplementary Information

## Figures and Tables

**Table 1:** Summary of output statistics from selected *A. suturalis* developmental stages.

**Samples**	**Total Raw Reads**	**Total Clean Reads**	**Total Clean Nucleotides (bp)**	**Q20 %**	**GC %**
eggs	65,321,014	55,038,084	4,953,427,560	97.64%	41.27%
2nd	63,297,708	54,099,184	4,868,926,560	98.34%	44.98%
5th	65,236,428	51,876,024	4,668,842,160	97.72%	43.10%
AFA	60,988,212	51,295,410	4,616,586,900	98.65%	43.24%
AMA	60,094,480	51,904,132	4,671,371,880	98.58%	44.01%
All	314,937,842	264,212,834	23,779,155,060		

Life stages are eggs, 2^nd^ instar nymphs (2nd), 5^th^ instar nymphs (5th), adult females (AFA), and adult males (AMA).

**Table 2:** Summary of assembly statistics from different developmental stages of *A. suturalis.*

	**Sample**	**Total Number**	**Total Length (bp)**	**Mean Length (bp)**	**N50**	**Total Consensus Sequences**	**Distinct Clusters**	**Distinct Singletons**
Contig	eggs	120,081	41,567,566	346	717	—	—	—
	2nd	159,448	45,674,162	286	417	—	—	—
	5th	172,589	45,866,576	266	364	—	—	—
	AFA	108,070	35,269,280	326	545	—	—	—
	AMA	117,117	35,246,492	301	468	—	—	—
	All	677,305	20,3624,076					
Unigene	eggs	70,195	42,719,920	609	1,063	70,195	15,085	55,110
	2nd	82,834	44,164,544	533	820	82,834	15,107	67,727
	5th	82,762	42,097,221	509	764	82,762	14,175	68,587
	AFA	61,212	35,484,816	580	927	61,212	10,977	50,235
	AMA	62,028	35,428,426	571	887	62,028	11,661	50,367
	ALL	88,614	70,479,040	795	1,398	88,614	23,830	64,784

Life stages are eggs, 2^nd^ instar nymphs (2nd), 5^th^ instar nymphs (5th), adult females (AFA), and adult males (AMA).

**Table 3:** Functional annotations of *A. suturalis* total unigenes.

**Sequence File**	**NR**	**NT**	**Swiss-Prot**	**KEGG**	**COG**	**GO**	**ALL**
unigenes	27,392	11,026	21,564	19,204	10,351	12,121	29,971

**NR (**non-redundant database**), NT (**nucleotide database), Swiss-Prot (Swiss Protein database), **KEGG** (Kyoto Encyclopedia of Genes and Genomes), **COG** (Clusters of Orthologous Groups), **GO (**Gene Ontology).

**Table 4:** Summary of single nucleotide polymorphisms (SNPs) including transition and transversion base changes identified from analyses of transcriptomic data from *A. suturalis*.

**SNP Type**	**eggs**	**2nd**	**5th**	**AFA**	**AMA**	**Total**
Transition	125,800	97,988	87,646	81,653	77,590	470,677
A-G	63,306	49,191	44,195	41,101	39,225	237,018
C-T	62,494	48,797	43,451	40,552	38,365	233,659
Transversion	83,077	70,797	61,421	54,518	50,900	320,713
A-C	19,820	17,294	14,848	13,125	12,251	77,338
A-T	30,986	25,517	22,285	19,998	18,176	116,962
C-G	12,439	10,755	9,231	8,179	8,008	48,612
G-T	19,832	17,231	15,057	13,216	12,465	77,801
Total	208,877	168,785	149,067	136,171	128,490	791,390

Developmental stages are eggs, 2^nd^ and 5^th^ instar nymph stages (‘2nd’ and ‘5th’, respectively), male (AMA) and female (AFA) adult life stages.
