# Supplementary material for: Characterization of Adelphocoris suturalis (Hemiptera: Miridae) Transcriptome from Different Developmental Stages
Source: Sci Rep. 2015 Jun 5;5:11042. doi: 10.1038/srep11042 (PMC4457133; doi:10.1038/srep11042)
Supplement: Supplementary Information [file srep11042-s1.pdf]

Supplementary information

Characterization of *Adelphocoris suturalis* (Hemiptera: Miridae) Transcriptome from Different Developmental stages

Caihong Tian[1], Wee Tek Tay[2], Hongqiang Feng[1*], Ying Wang[1], Yongmin Hu[1], Guoping Li[1]

[1]Henan Key Laboratory of Crop Pest Control, MOA Key Regional Crop Integrated Pest Management (IPM) Laboratory in Southern Part of Northern China, Institute of Plant Protection, Henan Academy of Agricultural Sciences, Zhengzhou 450002, China.

[2] CSIRO, Clunies Ross Street, ACT 2601, Australia.

[*] Author for correspondence:

Hongqiang Feng, PhD, Professor

Henan Key Laboratory of Crop Pest Control, MOA Key Regional Crop Integrated Pest Management (IPM) Laboratory in Southern Part of Northern China, Institute of Plant Protection, Henan Academy of Agricultural Sciences, Zhengzhou 450002, China.

Phone: +86-0371-6571-7371, Fax: +86-0371-6571-7371

E-mail: feng_hq@163.com

Table S1: Summary for lengths of *A. suturalis* unigenes

sequence size	all unigenes	eggs	2nd instar nymphs	5th instar nymphs	AFA	AMA
200	0	18357	21014	11801	12760	15000
300	28459	22731	20545	15807	14900	17889
400	14492	11334	11815	8514	8823	8691
500	7997	6424	6417	4848	5095	5199
600	5486	4395	4230	3386	3579	3619
700	4086	3200	3115	2534	2709	2676
800	3203	2416	2398	2019	2153	2137
900	2661	1984	1884	1611	1657	1761
1000	2220	1639	1639	1396	1451	1525
1100	1856	1307	1291	1088	1099	1269
1200	1589	1093	1061	1003	963	1073
1300	1496	934	968	878	871	996
1400	1286	829	844	718	742	848
1500	1172	733	646	679	637	753
1600	1058	623	603	615	584	667
1700	1002	533	542	568	462	621
1800	930	459	428	426	440	581
1900	829	407	392	407	341	519
2000	727	380	352	365	330	455
2100	671	343	284	296	256	416
2200	649	297	252	257	259	389
2300	540	230	228	216	168	346
2400	470	207	204	203	182	313
2500	482	187	200	189	177	292
2600	440	163	160	135	126	229
2700	380	157	163	122	132	186
2800	355	159	131	140	121	189
2900	357	130	103	109	99	178
3000	310	122	92	79	83	147
**≥3000**	**3411**	**1061**	**761**	**803**	**829**	**1231**
**Total ≥1000bp**	**22230**	**11993**	**11344**	**10692**	**10352**	**13223**

Table S2: KO annotations of *A. suturalis* unigenes

Pathway	Count (44442)	Pathway ID
Metabolic pathways	2737	ko01100
Regulation of actin cytoskeleton	804	ko04810
Focal adhesion	744	ko04510
RNA transport	703	ko03013
Huntington's disease	632	ko05016
Amoebiasis	617	ko05146
Pathways in cancer	613	ko05200
Spliceosome	564	ko03040
Purine metabolism	563	ko00230
Vascular smooth muscle contraction	557	ko04270
Neuroactive ligand-receptor interaction	552	ko04080
Bile secretion	530	ko04976
Epstein-Barr virus infection	525	ko05169
Ubiquitin mediated proteolysis	520	ko04120
Endocytosis	518	ko04144
Tight junction	496	ko04530
Vibrio cholerae infection	476	ko05110
Dilated cardiomyopathy	458	ko05414
Hypertrophic cardiomyopathy (HCM)	450	ko05410
HTLV-I infection	434	ko05166
mRNA surveillance pathway	429	ko03015
Lysosome	428	ko04142
Protein processing in endoplasmic reticulum	425	ko04141
Herpes simplex infection	420	ko05168
Pyrimidine metabolism	411	ko00240
Calcium signaling pathway	401	ko04020
ABC transporters	400	ko02010
Phagosome	398	ko04145
Transcriptional misregulation in cancer	391	ko05202
Cell cycle	389	ko04110
Salmonella infection	384	ko05132
Alzheimer's disease	378	ko05010
Insulin signaling pathway	373	ko04910
Influenza A	372	ko05164
Protein digestion and absorption	345	ko04974
MAPK signaling pathway	345	ko04010
Amyotrophic lateral sclerosis (ALS)	340	ko05014

Tuberculosis	338	ko05152
Progesterone-mediated oocyte maturation	336	ko04914
Cardiac muscle contraction	331	ko04260
Pancreatic secretion	318	ko04972
Vasopressin-regulated water reabsorption	316	ko04962
Gastric acid secretion	309	ko04971
Viral myocarditis	306	ko05416
Ribosome	299	ko03010
ECM-receptor interaction	299	ko04512
Chemokine signaling pathway	293	ko04062
Lysine degradation	286	ko00310
Pathogenic *Escherichia coli* infection	279	ko05130
Adherens junction	278	ko04520
RNA polymerase	275	ko03020
Starch and sucrose metabolism	273	ko00500
Oxidative phosphorylation	272	ko00190
Wnt signaling pathway	271	ko04310
Parkinson's disease	269	ko05012
Ribosome biogenesis in eukaryotes	258	ko03008
Salivary secretion	255	ko04970
Axon guidance	253	ko04360
Fc gamma R-mediated phagocytosis	244	ko04666
Leukocyte transendothelial migration	234	ko04670
Bacterial invasion of epithelial cells	231	ko05100
Oocyte meiosis	231	ko04114
Toxoplasmosis	229	ko05145
Glutamatergic synapse	228	ko04724
Glycerophospholipid metabolism	226	ko00564
Peroxisome	223	ko04146
Dopaminergic synapse	214	ko04728
Galactose metabolism	212	ko00052
Drug metabolism - other enzymes	206	ko00983
Small cell lung cancer	202	ko05222
Neurotrophin signaling pathway	200	ko04722
Phosphatidylinositol signaling system	198	ko04070
Glycerolipid metabolism	191	ko00561
Pentose and glucuronate interconversions	189	ko00040
Dorso-ventral axis formation	187	ko04320
Shigellosis	185	ko05131
T-cell receptor signaling pathway	184	ko04660

Prostate cancer	181	ko05215
Alcoholism	175	ko05034
Arrhythmogenic right ventricular cardiomyopathy (ARVC)	173	ko05412
Glycolysis / Gluconeogenesis	167	ko00010
RNA degradation	167	ko03018
Carbohydrate digestion and absorption	163	ko04973
VEGF signaling pathway	163	ko04370
Melanogenesis	162	ko04916
Synaptic vesicle cycle	158	ko04721
Long-term potentiation	158	ko04720
GnRH signaling pathway	157	ko04912
Basal transcription factors	154	ko03022
Antigen processing and presentation	147	ko04612
Fat digestion and absorption	146	ko04975
Cell adhesion molecules (CAMs)	145	ko04514
Amphetamine addiction	144	ko05031
Inositol phosphate metabolism	143	ko00562
Measles	143	ko05162
ErbB signaling pathway	143	ko04012
PPAR signaling pathway	142	ko03320
Amino sugar and nucleotide sugar metabolism	139	ko00520
Cytokine-cytokine receptor interaction	139	ko04060
Arginine and proline metabolism	138	ko00330
GABAergic synapse	137	ko04727
Retrograde endocannabinoid signaling	137	ko04723
Fanconi anemia pathway	137	ko03460
Hepatitis C	136	ko05160
Pyruvate metabolism	135	ko00620
Renal cell carcinoma	134	ko05211
Metabolism of xenobiotics by cytochrome P450	134	ko00980
Morphine addiction	132	ko05032
Glutathione metabolism	131	ko00480
Drug metabolism - cytochrome P450	130	ko00982
Cytosolic DNA-sensing pathway	129	ko04623
Cocaine addiction	129	ko05030
Phototransduction - fly	128	ko04745
Prion diseases	126	ko05020
Gap junction	123	ko04540
Rheumatoid arthritis	123	ko05323
Olfactory transduction	122	ko04740

Epithelial cell signaling in *Helicobacter pylori* infection	122	ko05120
Cholinergic synapse	119	ko04725
Glycine, serine and threonine metabolism	117	ko00260
Legionellosis	116	ko05134
Glioma	116	ko05214
Retinol metabolism	115	ko00830
Glycosaminoglycan biosynthesis - heparan sulfate	114	ko00534
Other types of O-glycan biosynthesis	114	ko00514
Complement and coagulation cascades	112	ko04610
Serotonergic synapse	111	ko04726
Steroid hormone biosynthesis	110	ko00140
TGF-beta signaling pathway	110	ko04350
Adipocytokine signaling pathway	109	ko04920
Mineral absorption	108	ko04978
mTOR signaling pathway	108	ko04150
Hedgehog signaling pathway	104	ko04340
Endometrial cancer	104	ko05213
Pentose phosphate pathway	104	ko00030
Porphyrin and chlorophyll metabolism	103	ko00860
Osteoclast differentiation	103	ko04380
Colorectal cancer	103	ko05210
Fructose and mannose metabolism	102	ko00051
Apoptosis	100	ko04210
p53 signaling pathway	99	ko04115
Notch signaling pathway	99	ko04330
Endocrine and	97	ko04961
Base excision repair	97	ko03410
Ascorbate and aldarate metabolism	97	ko00053
Vitamin digestion and absorption	96	ko04977
NF-kappa B signaling pathway	94	ko04064
Chronic myeloid leukemia	94	ko05220
Citrate cycle (TCA cycle)	93	ko00020
Cysteine and methionine metabolism	90	ko00270
Jak-STAT signaling pathway	90	ko04630
Phototransduction	88	ko04744
Nucleotide excision repair	87	ko03420
Type II diabetes mellitus	87	ko04930
Tryptophan metabolism	85	ko00380
Systemic lupus erythematosus	84	ko05322
N-Glycan biosynthesis	84	ko00510

alpha-Linolenic acid metabolism	83	ko00592
Natural killer cell mediated cytotoxicity	83	ko04650
B cell receptor signaling pathway	83	ko04662
Basal cell carcinoma	82	ko05217
Staphylococcus aureus infection	82	ko05150
Pertussis	78	ko05133
Long-term depression	78	ko04730
Toll-like receptor signaling pathway	78	ko04620
Fatty acid elongation	78	ko00062
Chagas disease (American trypanosomiasis)	76	ko05142
Pancreatic cancer	76	ko05212
DNA replication	76	ko03030
Collecting duct acid secretion	75	ko04966
Valine, leucine and isoleucine degradation	75	ko00280
Fc epsilon RI signaling pathway	74	ko04664
Aminoacyl-tRNA biosynthesis	74	ko00970
Thyroid cancer	73	ko05216
Primary immunodeficiency	72	ko05340
Tyrosine metabolism	72	ko00350
Nicotine addiction	69	ko05033
Hematopoietic cell lineage	69	ko04640
Acute myeloid leukemia	67	ko05221
NOD-like receptor signaling pathway	66	ko04621
Proteasome	66	ko03050
Non-small cell lung cancer	64	ko05223
Arachidonic acid metabolism	63	ko00590
Alanine, aspartate and glutamate metabolism	63	ko00250
Fatty acid metabolism	62	ko00071
Glyoxylate and dicarboxylate metabolism	59	ko00630
Propanoate metabolism	58	ko00640
beta-Alanine metabolism	58	ko00410
Terpenoid backbone biosynthesis	56	ko00900
Homologous recombination	55	ko03440
Glycosylphosphatidylinositol (GPI)-anchor biosynthesis	55	ko00563
Butanoate metabolism	55	ko00650
Melanoma	54	ko05218
Proximal tubule bicarbonate reclamation	54	ko04964
Insect hormone biosynthesis	52	ko00981
Ether lipid metabolism	52	ko00565
Fatty acid biosynthesis	52	ko00061

Biosynthesis of unsaturated fatty acids	52	ko01040
Bladder cancer	52	ko05219
Circadian rhythm - fly	51	ko04711
SNARE interactions in vesicular transport	51	ko04130
Glycosaminoglycan degradation	49	ko00531
MAPK signaling pathway - fly	49	ko04013
Malaria	47	ko05144
Sphingolipid metabolism	47	ko00600
Renin-angiotensin system	46	ko04614
Linoleic acid metabolism	46	ko00591
Aldosterone-regulated sodium reabsorption	46	ko04960
Leishmaniasis	45	ko05140
RIG-I-like receptor signaling pathway	45	ko04622
Mismatch repair	41	ko03430
Nicotinate and nicotinamide metabolism	41	ko00760
Riboflavin metabolism	40	ko00740
Phenylalanine metabolism	38	ko00360
Other glycan degradation	38	ko00511
Protein export	37	ko03060
Circadian rhythm - mammal	37	ko04710
Histidine metabolism	37	ko00340
Caffeine metabolism	36	ko00232
Taste transduction	36	ko04742
Steroid biosynthesis	33	ko00100
Glycosaminoglycan biosynthesis - chondroitin sulfate	33	ko00532
Glycosphingolipid biosynthesis - lacto and neolacto series	30	ko00601
Folate biosynthesis	29	ko00790
Selenocompound metabolism	27	ko00450
Pantothenate and CoA biosynthesis	26	ko00770
Glycosaminoglycan biosynthesis - keratan sulfate	25	ko00533
Mucin type O-Glycan biosynthesis	24	ko00512
Maturity onset diabetes of the young	23	ko04950
One carbon pool by folate	23	ko00670
Ubiquinone and other terpenoid-quinone biosynthesis	22	ko00130
Sulfur metabolism	22	ko00920
Non-homologous end-joining	22	ko03450
Glycosphingolipid biosynthesis - globo series	21	ko00603
Type I diabetes mellitus	20	ko04940

Regulation of autophagy	19	ko04140
Autoimmune thyroid disease	19	ko05320
Sulfur relay system	18	ko04122
Cyanoamino acid metabolism	17	ko00460
Butirosin and neomycin biosynthesis	16	ko00524
D-Arginine and D-ornithine metabolism	15	ko00472
Valine, leucine and isoleucine biosynthesis	15	ko00290
Vitamin B6 metabolism	15	ko00750
Taurine and hypotaurine metabolism	13	ko00430
African trypanosomiasis	13	ko05143
D-Glutamine and D-glutamate metabolism	11	ko00471
Synthesis and degradation of ketone bodies	11	ko00072
Glycosphingolipid biosynthesis - ganglio series	10	ko00604
Biotin metabolism	9	ko00780
Primary bile acid biosynthesis	8	ko00120
Phenylalanine, tyrosine and tryptophan biosynthesis	7	ko00400
Lipoic acid metabolism	4	ko00785
Lysine biosynthesis	4	ko00300
Asthma	3	ko05310
Thiamine metabolism	3	ko00730
Allograft rejection	1	ko05330
Graft-versus-host disease	1	ko05332
Intestinal immune network for IgA production	1	ko04672

Table S3: SNPs identified from the targeted developmental stages of *A. suturalis*

SNP Type	Eggs (%)	2nd (%)	5th (%)	AFA (%)	AMA (%)	Total (%)
Transition	60.23	58.05	58.80	59.96	60.39	59.47
A-G	30.31	29.14	29.65	30.18	30.53	29.95
C-T	29.92	28.91	29.15	29.78	29.86	29.53
Transversion	39.77	41.95	41.20	40.04	39.61	40.53
A-C	9.49	10.25	9.96	9.64	9.53	9.77
A-T	14.83	15.12	14.95	14.69	14.15	14.78
C-G	5.96	6.37	6.19	6.01	6.23	6.14
G-T	9.49	10.21	10.10	9.71	9.70	9.83

Table S4: Top ten differentially expressed genes in each library identified from comparison between different *A. suturalis* life stages

Comparison	Unigene	Hit Number	Description	FDR	Fold Change
	Unigene32031_All	--	--	0.00	23.24
	Unigene1946_All	gi|91081177|ref|XP_975589.1|	PREDICTED: similar to CG32603 CG32603-PA [*Tribolium castaneum*]	0.00	21.06
	CL6720.Contig2_All	gi|223670982|tpd|FAA00442.1|	TPA: putative cuticle protein [*Bombyx mori*]	0.00	20.67
	CL5666.Contig2_All	gi|449266330|gb|EMC77394.1|	putative serine/threonine-protein kinase kinX [*Columba livia*]	0.00	19.95
**2nd vs Eggs**	CL1468.Contig1_All	gi|307175910|gb|EFN65723.1|	Cuticle protein 21 [*Camponotus floridanus*]	0.00	19.84
	Unigene226_All	gi|297302112|ref|XP_001113817.2|	PREDICTED: type I inositol-1,4,5-trisphosphate 5-phosphatase-like [*Macaca mulatta*]	0.00	19.83
	Unigene27671_All	gi|270000362|gb|ACZ58028.1|	odorant-binding protein 2 [*Adelphocoris lineolatus*]	0.00	19.79
	Unigene37865_All	gi|74778856 |sp|P82166.1|CU198_LOCMI	RecName: Full=Cuticle protein 19.8; AltName: Full=LmNCP19.8	0.00	19.74
	CL8176.Contig1_All	--	--	0.00	19.69
	CL3319.Contig1_All	gi|470247497|ref|XP_004357478.1|	cysteine proteinase [*Dictyostelium fasciculatum*]	0.00	19.63
	Unigene37393_All	--	--	0.00	-18.09
	Unigene28710_All	gi|449678845|ref |XP_004209172.1|	PREDICTED: uncharacterized protein LOC101240627 [*Hydra magnipapillata*]	0.00	-18.76
	CL606.Contig3_All	gi|307170134|gb|EFN62552.1|	Proclotting enzyme [*Camponotus floridanus*]	0.00	-19.02
	CL306.Contig53_All	gi|195435271|ref |XP_002065625.1|	GK14571 [*Drosophila willistoni*]	0.10	-19.03
	CL5310.Contig1_All	--	--	0.10	-19.54
	Unigene12207_All	--	--	1.00	-19.82

	Unigene16020_All	gi|189239425|ref |XP_001814901.1|	PREDICTED: similar to Toutatis [*Tribolium castaneum*]	1.00	-20.01
	Unigene18074_All	gi|332026407|gb|EGI66536.1|	Longitudinals lacking protein, isoforms A/B/D/L [*Acromyrmex echinatior*]	1.00	-20.14
	Unigene20241_All	gi|270012195|gb|EFA08643.1|	hypothetical protein TcasGA2_TC006306 [*Tribolium castaneum*]	1.00	-20.20
	Unigene20645_All	gi|307201550|gb|EFN81313.1|	Protein transport protein Sec24C [*Harpegnathos saltator*]	1.00	-20.54
**5th vs 2nd**	Unigene16431_All	--	--	0.00	17.92
	Unigene39147_All	--	--	0.00	17.69
	CL1428.Contig2_All	--	--	0.00	17.39
	Unigene12532_All	gi|270011761|gb|EFA08209.1|	hypothetical protein TcasGA2_TC005836 [*Tribolium castaneum*]	0.00	17.35
	Unigene39096_All	--	--	0.00	16.96
	Unigene8506_All	gi|332016388|gb|EGI57301.1|	Cytochrome c oxidase subunit 6B1 [*Acromyrmex echinatior*]	0.00	16.69
	Unigene31684_All	--	--	0.00	16.62
	Unigene39345_All	gi|332372957|gb|AEE61620.1|	unknown [*Dendroctonus ponderosae*]	0.00	16.61
	Unigene35540_All	gi|268557034|ref| XP_002636506.1|	Hypothetical protein CBG23181 [*Caenorhabditis briggsae*]	0.00	16.47
	Unigene8752_All	--	--	0.00	16.45
	Unigene14053_All	--	--	0.00	-13.29
	Unigene1013_All	gi|350407083|ref| XP_003487978.1|	PREDICTED: putative transporter SVOPL-like [*Bombus impatiens*]	0.00	-13.36
	CL1721.Contig3_All	gi|383851209|ref |XP_003701131.1|	PREDICTED: dolichyl-diphosphooligosaccharide--protein glycosyltransferase 48 kDa subunit-like [*Megachile rotundata]*	0.00	-13.57
	CL6430.Contig2_All	gi|322801079 |gb|EFZ21832.1|	hypothetical protein SINV_03497 [*Solenopsis invicta*]	1.00	-14.10
	Unigene21390_All	gi|149898790 |gb|ABR27857.1|	predicted RNA-binding protein [*Triatoma infestans*]	1.00	-14.31
	Unigene509_All	--	--	1.00	-14.78

	Unigene18264_All	gi|242022735|ref|XP_002431794.1|	translocon-associated protein subunit delta precursor, putative [*Pediculus humanus corporis*]	1.00	-14.92
	CL7700.Contig1_All	gi|170043906|ref|XP_001849608.1|	sugar transporter [*Culex quinquefasciatus*] >gi|167867183|gb|EDS30566.1| sugar transporter [*Culex quinquefasciatus*]	1.00	-14.95
	Unigene14444_All	--	--	0.99	-15.03
	CL5634.Contig2_All	gi|193706883|ref|XP_001946924.1|	PREDICTED: regulator of G-protein signaling 12-like [*Acyrthosiphon pisum*]	0.99	-15.10
**AFA vs 5th**	CL1016.Contig3_All	gi|194741178|ref|	GF17401 [*Drosophila ananassae*]	0.00	21.64
	Unigene63645_All	--	--	0.00	21.42
	Unigene8962_All	gi|432946172|ref|XP_004083803.1|	PREDICTED: cathepsin B-like [*Oryzias latipes*]	0.00	20.86
	Unigene3413_All	gi|390337642|ref|XP_780653.3|	PREDICTED: cathepsin L-like [*Strongylocentrotus purpuratus*]	0.00	20.49
	CL3475.Contig2_All	--	--	0.00	20.40
	Unigene39139_All	gi|122720928|gb|ABM66454.1|	RP45 [*Rhodnius prolixus*]	0.00	20.28
	CL7556.Contig1_All	gi|341891084|gb|EGT47019.1|	CBN-CPR-4 protein [*Caenorhabditis brenneri*]	0.00	20.27
	CL1353.Contig1_All	gi|195046414|ref|XP_001992148.1|	GH24370 [*Drosophila grimshawi*]	0.00	20.12
	Unigene60991_All	gi|241742198|ref|XP_002414167.1|	Opacity protein and related surface antigens [*Ixodes scapularis*]	0.00	20.11
	CL7714.Contig2_All	gi|40557501|gb|AAR88049.1|	reproduction-associated glycoprotein 7 [*Odocoileus virginianus*]	0.00	20.08
	Unigene20697_All	gi|70907192|gb|AAZ15237.1|	reverse transcriptase [*Aedes aegypti*]	0.00	-17.29
	CL3245.Contig2_All	gi|442629659|ref|	sallimus, isoform Z [*Drosophila melanogaster*]	0.00	-17.32

		NP_001261314.1|			
	Unigene20687_All	--	--	0.00	-17.51
	CL4459.Contig2_All	--	--	0.00	-17.77
	CL4042.Contig1_All	--	--	0.00	-17.77
	CL21.Contig5_All	gi|301605844|ref| XP_002932560.1|	PREDICTED: general transcription factor II-I repeat domain-containing protein 2A-like [*Xenopus* (*Silurana*) *tropicalis*]	0.00	-18.05
	Unigene21592_All	gi|391336237|ref| XP_003742488.1|	PREDICTED: RNA-directed DNA polymerase from mobile element jockey-like [*Metaseiulus occidentalis*]	0.00	-18.38
	CL4105.Contig2_All	--	--	0.00	-18.80
	Unigene9998_All	--	--	0.00	-19.07
	Unigene21149_All	gi|328723074|ref| XP_001943552.2|	PREDICTED: high affinity cAMP-specific and IBMX-insensitive 3',5'-cyclic phosphodiesterase 8A-like, partial [*Acyrthosiphon pisum*]	0.00	-19.76
	CL9440.Contig1_All	--	--	0.00	22.45
	CL9440.Contig5_All	--	--	0.00	21.86
	CL3400.Contig2_All	--	--	0.00	21.63
	Unigene62783_All	--	--	0.00	21.03
	Unigene63401_All	--	--	0.00	20.95
	CL9440.Contig2_All	--	--	0.00	20.95
**AMA vs 5th**	CL5387.Contig1_All	--	--	0.00	20.87
	Unigene62177_All	--	--	0.00	20.81
	CL9440.Contig4_All	--	--	0.00	20.73
	Unigene32111_All	--	--	0.00	20.59
	CL1504.Contig1_All	gi|270011046|gb|EFA07494.1|	exuperantia [*Tribolium castaneum*]	0.00	-17.51
	Unigene28405_All	--	--	0.00	-17.51
	Unigene36335_All	gi|443721234|gb|ELU10627.1|	hypothetical protein CAPTEDRAFT_141578, partial [*Capitella teleta*]	0.00	-17.77

	CL7402.Contig1_All	--	--	0.00	-17.77
	Unigene16871_All	--	--	0.00	-18.06
	Unigene24676_All	gi|242020390|ref XP_002430638.1|	ankyrin repeat-containing protein [*Pediculus humanus corporis*]	0.00	-18.14
	CL715.Contig4_All	--	--	0.00	-18.25
	CL7996.Contig1_All	gi|193713722|ref |XP_001942734.1|	PREDICTED: hypothetical protein LOC100160904 [*Acyrthosiphon pisum*]	0.00	-18.49
	CL2789.Contig4_All	gi|332374518|gb| AEE62400.1|	unknown [*Dendroctonus ponderosae*]	0.00	-18.80
	CL2443.Contig4_All	gi|149243127|pdb|2PG1|A	Dynein light chain 1, cytoplasmic OS=*Drosophila melanogaster*	0.00	-20.48
**AFA vs eggs**	Unigene36723_All	gi|122720928|gb|ABM66454.1|	RP45 [*Rhodnius prolixus*]	0.00	23.73
	Unigene32426_All	gi|312190375|dbj|BAJ33507.1|	vitellogenin [*Trigonotylus caelestialium*]	0.00	22.75
	CL1353.Contig4_All	gi|312190375|dbj|BAJ33507.1|	vitellogenin [*Trigonotylus caelestialium*]	0.00	21.66
	CL718.Contig1_All	gi|94421564|gb|ABF18889.1|	cathepsin-L [*Lygus lineolaris*]	0.00	21.65
	CL1016.Contig3_All	gi|194741178|ref| XP_001953066.1|	GF17401 [*Drosophila ananassae*]	0.00	21.64
	Unigene63658_All	gi|21357507|ref| NP_650264.1|	CG8483 [*Drosophila melanogaster*]	0.00	21.57
	CL3475.Contig6_All	--	--	0.00	21.49
	Unigene63645_All	--	--	0.00	21.42
	CL3677.Contig7_All	gi|194218271|ref| XP_001501895.2|	PREDICTED: pepsin A-like [*Equus caballus*]	0.00	21.39
	Unigene26491_All	gi|122720928|gb| ABM66454.1|	RP45 [*Rhodnius prolixus*]	0.00	21.08
	CL2027.Contig2_All	gi|345487094|ref| XP_001600181.2|	PREDICTED: hypothetical protein LOC100115461 isoform 1 [*Nasonia vitripennis*]	0.00	-19.02

	Unigene5303_All	--	--	0.00	-19.03
	Unigene1694_All	--	--	0.00	-19.60
	Unigene36777_All	gi|350417343|ref| XP_003491376.1|	PREDICTED: ubiquitin carboxyl-terminal hydrolase 5-like [*Bombus impatiens*]	0.00	-19.81
	Unigene32741_All	gi|340724912|ref| XP_003400822.1|	PREDICTED: eukaryotic translation initiation factor 3 subunit B-like isoform 1 [*Bombus terrestris*]	0.00	-19.93
	CL7232.Contig4_All	gi|193700092|ref| XP_001951744.1|	PREDICTED: ABC transporter G family member 20-like isoform 1 [*Acyrthosiphon pisum*]	0.00	-20.01
	Unigene11277_All	--	--	0.00	-20.20
	CL550.Contig3_All	gi|270001965|gb| EEZ98412.1|	hypothetical protein TcasGA2_TC000880 [*Tribolium castaneum*]	0.00	-20.33
	Unigene32776_All	--	--	0.00	-20.36
	Unigene25341_All	gi|242012592|ref| XP_002427015.1|	conserved hypothetical protein [*Pediculus humanus corporis*]	0.00	-21.89
	CL7827.Contig1_All	--	--	0.00	18.30
	CL1284.Contig4_All	--	--	0.00	18.14
	CL9254.Contig1_All	--	--	0.00	17.97
	Unigene62205_All	--	--	0.00	17.71
	Unigene63160_All	--	--	0.00	17.68
	Unigene62060_All	gi|193713906|ref| XP_001943641.1|	PREDICTED: catalase-like [*Acyrthosiphon pisum*]	0.00	17.60
**AMA vs AFA**	Unigene62175_All	--	--	0.00	17.38
	Unigene63257_All	gi|62122751|ref| NP_001014299.1|	keratin, type II cytoskeletal 2 oral [*Canis lupus familiaris*]	0.00	17.33
	CL7184.Contig1_All	--	--	0.00	17.27

Unigene63313_All	--	--	0.00	17.19
CL2673.Contig5_All	gi|380020103|ref|XP_003693935.1|	PREDICTED: uncharacterized protein LOC100871420 [*Apis florea*]	0.00	-15.34
Unigene28851_All	--	--	0.00	-15.43
Unigene885_All	gi|270010258|gb|EFA06706.1|	hypothetical protein TcasGA2_TC009637 [*Tribolium castaneum*]	0.00	-15.50
CL1741.Contig3_All	gi|328705734|ref|XP_001948004.2|	PREDICTED: hypothetical protein LOC100165704 [*Acyrthosiphon pisum*]	0.00	-15.51
Unigene32880_All	gi|242017981|ref|XP_002429462.1|	zinc finger protein RTS2, putative [*Pediculus humanus corporis*]	0.00	-15.52
CL6647.Contig2_All	gi|307177781|gb|EFN66778.1|	Supporter of activation of yellow protein [*Camponotus floridanus*]	0.00	-15.59
Unigene6763_All	gi|158302472|ref|XP_322016.4|	AGAP001145-PA [*Anopheles gambiae str. PEST*]	0.00	-15.90
Unigene36589_All	gi|340729136|ref|XP_003402864.1|	PREDICTED: u4/U6 small nuclear ribonucleoprotein Prp31-like [*Bombus terrestris*]	0.00	-16.34
Unigene9291_All	gi|328783167|ref|XP_003250246.1|	PREDICTED: hypothetical protein LOC100576321 [*Apis mellifera*]	0.00	-16.76
CL5956.Contig2_All	--	--	0.00	-16.90

Table S5a: Gene set enrichment analysis comparing eggs and 2nd instar nymphs of *A. suturalis*

Category	Subcategory	Total
Biological Process	cellular process	4118
	single-organism process	3329
	metabolic process	3029
	biological regulation	2054
	regulation of biological process	1879
	multicellular organismal process	1850
	developmental process	1756
	response to stimulus	1545
	cellular component organization or biogenesis	1431
	localization	1210
	signaling	1058
	establishment of localization	1041
	reproduction	666
	reproductive process	593
	positive regulation of biological process	519
	negative regulation of biological process	500
	locomotion	436
	multi-organism process	310
	immune system process	293
	growth	281
	biological adhesion	151
	rhythmic process	68
Cellular Component	cell	3067
	cell part	3067
	organelle	2152
	membrane	1227
	organelle part	1217
	macromolecular complex	1132
	membrane part	734
	membrane-enclosed lumen	415
	extracellular region	180
	synapse	179
	cell junction	151
	synapse part	137
	extracellular region part	112
	extracellular matrix	57

	extracellular matrix part	23
	nucleoid	2
	virion	1
	virion part	1
Molecular Function	binding	3038
	catalytic activity	2869
	transporter activity	476
	structural molecule activity	291
	molecular transducer activity	194
	enzyme regulator activity	190
	nucleic acid binding transcription factor activity	187
	receptor activity	173
	protein binding transcription factor activity	66
	electron carrier activity	31
	antioxidant activity	19
	channel regulator activity	11
	translation regulator activity	7
	receptor regulator activity	4
	morphogen activity	3
	metallochaperone activity	1
	protein tag	1

Table S5b: Gene set pathway analysis comparing eggs and 2nd instar nymphs of *A. suturalis*

	Pathway	Eggs/2nd (23181)	Unigenes (44431)	P-value	Q-value	Pathway ID
1	Metabolic pathways	1302	2737	1	1	ko01100
2	Regulation of actin cytoskeleton	444	804	0.3391196	0.9473232	ko04810
3	RNA transport	394	703	0.2038002	0.6983554	ko03013
4	Amoebiasis	376	617	0.000545745	0.01148601	ko05146
5	Focal adhesion	376	744	0.9867324	1	ko04510
6	Pathways in cancer	362	613	0.01093412	0.1040766	ko05200
7	*Vibrio cholerae* infection	310	476	1.08862E-06	0.000139888	ko05110
8	Hypertrophic cardiomyopathy (HCM)	298	450	1.84691E-07	4.74655E-05	ko05410
9	Dilated cardiomyopathy	298	458	1.96797E-06	0.000168589	ko05414
10	Epstein-Barr virus infection	296	525	0.1956107	0.6793507	ko05169
11	Purine metabolism	292	563	0.9016257	1	ko00230
12	Spliceosome	281	564	0.9886218	1	ko03040
13	Tight junction	279	496	0.2204519	0.735794	ko04530
14	Endocytosis	276	518	0.7208232	1	ko04144
15	Vascular smooth muscle contraction	268	557	0.9989715	1	ko04270
16	Ubiquitin mediated proteolysis	267	520	0.9309437	1	ko04120
17	Neuroactive ligand-receptor interaction	248	552	0.9999978	1	ko04080
18	HTLV-I infection	244	434	0.2415926	0.7761162	ko05166
19	Herpes simplex infection	239	420	0.1652711	0.6546988	ko05168
20	mRNA surveillance pathway	239	429	0.3150846	0.9307672	ko03015
21	Pyrimidine metabolism	237	411	0.1011737	0.4727571	ko00240
22	Huntington's disease	231	632	1	1	ko05016
23	*Salmonella* infection	230	384	0.0169722	0.140705	ko05132
24	Transcriptional misregulation in cancer	223	391	0.1620318	0.6546988	ko05202
25	Influenza A	212	372	0.1732277	0.6546988	ko05164
26	MAPK signaling pathway	210	345	0.008835044	0.08733101	ko04010
27	Cell cycle	210	389	0.5939732	1	ko04110
28	Bile secretion	207	530	1	1	ko04976
29	Protein processing in endoplasmic reticulum	205	425	0.995905	1	ko04141
30	Lysosome	203	428	0.9986073	1	ko04142
31	Cardiac muscle contraction	197	331	0.03459138	0.2222496	ko04260
32	Phagosome	197	398	0.9798724	1	ko04145
33	Viral myocarditis	185	306	0.01886764	0.1515307	ko05416
34	Insulin signaling pathway	182	373	0.9881623	1	ko04910
35	ECM-receptor interaction	179	299	0.03265357	0.2154843	ko04512
36	Calcium signaling pathway	178	401	0.9999817	1	ko04020

37	Chemokine signaling pathway	177	293	0.0220575	0.1717811	ko04062
38	Ribosome	175	299	0.08509895	0.428832	ko03010
39	Protein digestion and absorption	175	345	0.9270442	1	ko04974
40	Pathogenic *Escherichia coli* infection	172	279	0.00856073	0.08733101	ko05130
41	Tuberculosis	172	338	0.9161575	1	ko05152
42	Ribosome biogenesis in eukaryotes	166	258	0.00074767	0.01372509	ko03008
43	Progesterone-mediated oocyte maturation	166	336	0.9728573	1	ko04914
44	Axon guidance	162	253	0.001177526	0.01891401	ko04360
45	Adherens junction	158	278	0.2285443	0.7530242	ko04520
46	Bacterial invasion of epithelial cells	154	231	0.000096667	0.00276038	ko05100
47	Wnt signaling pathway	151	271	0.3592028	0.9926357	ko04310
48	Alzheimer's disease	151	378	1	1	ko05010
49	Lysine degradation	149	286	0.806177	1	ko00310
50	Pancreatic secretion	149	318	0.9973781	1	ko04972
51	RNA polymerase	147	275	0.653662	1	ko03020
52	Vasopressin-regulated water reabsorption	141	316	0.9998316	1	ko04962
53	Leukocyte transendothelial migration	136	234	0.1424624	0.6014731	ko04670
54	Amyotrophic lateral sclerosis (ALS)	136	340	1	1	ko05014
55	Toxoplasmosis	135	229	0.0947419	0.4594088	ko05145
56	Fc gamma R-mediated phagocytosis	134	244	0.4672762	1	ko04666
57	ABC transporters	133	400	1	1	ko02010
58	Gastric acid secretion	132	309	0.9999879	1	ko04971
59	Glycerophospholipid metabolism	130	226	0.1932361	0.6793507	ko00564
60	Starch and sucrose metabolism	128	273	0.9950876	1	ko00500
61	Shigellosis	123	185	0.000547243	0.01148601	ko05131
62	Salivary secretion	123	255	0.9805192	1	ko04970
63	Oocyte meiosis	122	231	0.7153417	1	ko04114
64	Neurotrophin signaling pathway	119	200	0.08481344	0.428832	ko04722
65	Small cell lung cancer	118	202	0.1427621	0.6014731	ko05222
66	Peroxisome	116	223	0.7883909	1	ko04146
67	RNA degradation	112	167	0.000581005	0.01148601	ko03018
68	Drug metabolism - other enzymes	112	206	0.5378174	1	ko00983
69	Dopaminergic synapse	108	214	0.8932038	1	ko04728
70	Glutamatergic synapse	108	228	0.9868394	1	ko04724
71	Phosphatidylinositol signaling system	106	198	0.6301962	1	ko04070
72	Prostate cancer	105	181	0.1862322	0.672261	ko05215
73	Basal transcription factors	102	154	0.001884513	0.0284894	ko03022
74	T cell receptor signaling pathway	100	184	0.5412612	1	ko04660
75	Alcoholism	98	175	0.3684131	0.9964466	ko05034
76	Glycerolipid metabolism	97	191	0.8630024	1	ko00561

77	Measles	95	143	0.002300444	0.03284523	ko05162
78	Pentose and glucuronate interconversions	95	189	0.8911832	1	ko00040
79	Arrhythmogenic right ventricular cardiomyopathy (ARVC)	94	173	0.5431933	1	ko05412
80	Cytosolic DNA-sensing pathway	92	129	6.08902E-05	0.002214288	ko04623
81	Dorso-ventral axis formation	87	187	0.9879406	1	ko04320
82	Parkinson's disease	87	269	1	1	ko05012
83	Hepatitis C	86	136	0.02329741	0.176101	ko05160
84	Fanconi anemia pathway	85	137	0.04338812	0.265494	ko03460
85	Inositol phosphate metabolism	85	143	0.1313459	0.5922087	ko00562
86	VEGF signaling pathway	85	163	0.7492517	1	ko04370
87	GABAergic synapse	84	137	0.06198514	0.3620496	ko04727
88	ErbB signaling pathway	84	143	0.1709486	0.6546988	ko04012
89	Galactose metabolism	82	212	0.9999987	1	ko00052
90	Melanogenesis	78	162	0.9547862	1	ko04916
91	Retrograde endocannabinoid signaling	77	137	0.3722135	0.9964466	ko04723
92	Fat digestion and absorption	77	146	0.6917558	1	ko04975
93	GnRH signaling pathway	77	157	0.9255675	1	ko04912
94	Renal cell carcinoma	76	134	0.3299549	0.9318507	ko05211
95	Metabolism of xenobiotics by cytochrome P450	76	134	0.3299549	0.9318507	ko00980
96	Amino sugar and nucleotide sugar metabolism	74	139	0.6461039	1	ko00520
97	Antigen processing and presentation	74	147	0.8613654	1	ko04612
98	Morphine addiction	72	132	0.5268739	1	ko05032
99	Drug metabolism - cytochrome P450	71	130	0.5208253	1	ko00982
100	TGF-beta signaling pathway	70	110	0.03178703	0.2154843	ko04350
101	Amphetamine addiction	69	144	0.9516218	1	ko05031
102	Cell adhesion molecules (CAMs)	68	145	0.9721026	1	ko04514
103	Carbohydrate digestion and absorption	68	163	0.9995958	1	ko04973
104	Synaptic vesicle cycle	67	158	0.9991136	1	ko04721
105	Long-term potentiation	67	158	0.9991136	1	ko04720
106	Oxidative phosphorylation	67	272	1	1	ko00190
107	Steroid hormone biosynthesis	65	110	0.1883377	0.672261	ko00140
108	Prion diseases	65	126	0.7695608	1	ko05020
109	PPAR signaling pathway	65	142	0.984725	1	ko03320
110	Chronic myeloid leukemia	64	94	0.00481998	0.06193674	ko05220
111	Colorectal cancer	64	103	0.06986723	0.3903452	ko05210
112	Retinol metabolism	64	115	0.4350289	1	ko00830
113	Glycine, serine and threonine metabolism	64	117	0.5161533	1	ko00260
114	Cholinergic synapse	64	119	0.5952005	1	ko04725

115	Pyruvate metabolism	64	135	0.9583564	1	ko00620
116	Nucleotide excision repair	62	87	0.000958833	0.01642801	ko03420
117	Jak-STAT signaling pathway	61	90	0.006812615	0.07958373	ko04630
118	Mineral absorption	61	108	0.3721792	0.9964466	ko04978
119	Endometrial cancer	60	104	0.2859166	0.8747686	ko05213
120	Phototransduction - fly	60	128	0.9649821	1	ko04745
121	Legionellosis	59	116	0.8083026	1	ko05134
122	DNA replication	58	76	6.50277E-05	0.002214288	ko03030
123	Base excision repair	58	97	0.1691442	0.6546988	ko03410
124	p53 signaling pathway	58	99	0.2338659	0.7608043	ko04115
125	Cocaine addiction	58	129	0.9879208	1	ko05030
126	Cytokine-cytokine receptor interaction	58	139	0.9990392	1	ko04060
127	Glycolysis / Gluconeogenesis	58	167	1	1	ko00010
128	Notch signaling pathway	57	99	0.3004668	0.8979066	ko04330
129	Glutathione metabolism	57	131	0.9953987	1	ko00480
130	Osteoclast differentiation	56	103	0.546939	1	ko04380
131	mTOR signaling pathway	55	108	0.7979681	1	ko04150
132	Serotonergic synapse	55	111	0.8716205	1	ko04726
133	Fructose and mannose metabolism	53	102	0.7279929	1	ko00051
134	Complement and coagulation cascades	53	112	0.9464346	1	ko04610
135	Epithelial cell signaling in *Helicobacter pylori* infection	53	122	0.994368	1	ko05120
136	Fc epsilon RI signaling pathway	51	74	0.007806645	0.08359616	ko04664
137	Aminoacyl-tRNA biosynthesis	51	74	0.007806645	0.08359616	ko00970
138	Toll-like receptor signaling pathway	51	78	0.03269995	0.2154843	ko04620
139	Natural killer cell mediated cytotoxicity	51	83	0.1201322	0.551321	ko04650
140	Other types of O-glycan biosynthesis	51	114	0.9852911	1	ko00514
141	Gap junction	51	123	0.998588	1	ko04540
142	*Staphylococcus aureus* infection	50	82	0.1402416	0.6014731	ko05150
143	B cell receptor signaling pathway	50	83	0.1707104	0.6546988	ko04662
144	Cysteine and methionine metabolism	50	90	0.4592209	1	ko00270
145	Vitamin digestion and absorption	50	96	0.7158748	1	ko04977
146	Porphyrin and chlorophyll metabolism	50	103	0.9039064	1	ko00860
147	Glioma	50	116	0.9945991	1	ko05214
148	Olfactory transduction	50	122	0.998971	1	ko04740
149	NF-kappa B signaling pathway	49	94	0.7116508	1	ko04064
150	Pancreatic cancer	48	76	0.0781706	0.4099968	ko05212
151	Ascorbate and aldarate metabolism	48	97	0.8611943	1	ko00053
152	Chagas disease (American trypanosomiasis)	46	76	0.170939	0.6546988	ko05142
153	Fatty acid elongation	46	78	0.2455903	0.7780358	ko00062
154	Adipocytokine signaling pathway	46	109	0.9961604	1	ko04920

155	Glycosylphosphatidylinositol(GPI)-anchor biosynthesis	45	55	1.9268E-05	0.001237968	ko00563
156	Pertussis	45	78	0.322855	0.9318507	ko05133
157	Homologous recombination	44	55	6.89272E-05	0.002214288	ko03440
158	Nicotine addiction	44	69	0.07462701	0.3995654	ko05033
159	Basal cell carcinoma	44	82	0.6016438	1	ko05217
160	N-Glycan biosynthesis	44	84	0.6889676	1	ko00510
161	Glycosaminoglycan biosynthesis - heparan sulfate	44	114	0.9997675	1	ko00534
162	Hedgehog signaling pathway	43	104	0.997316	1	ko04340
163	Pentose phosphate pathway	43	104	0.997316	1	ko00030
164	Tyrosine metabolism	42	72	0.2939336	0.8887169	ko00350
165	Rheumatoid arthritis	41	123	0.9999993	1	ko05323
166	Hematopoietic cell lineage	40	69	0.3211455	0.9318507	ko04640
167	Type II diabetes mellitus	40	87	0.9549534	1	ko04930
168	Arginine and proline metabolism	40	138	1	1	ko00330
169	Endocrine and other factor-regulated calcium reabsorption	39	97	0.9982599	1	ko04961
170	Non-small cell lung cancer	38	64	0.2531357	0.7838057	ko05223
171	Long-term depression	38	78	0.871048	1	ko04730
172	alpha-Linolenic acid metabolism	38	83	0.9550442	1	ko00592
173	Tryptophan metabolism	38	85	0.972118	1	ko00380
174	Circadian rhythm - fly	37	51	0.006164542	0.07544225	ko04711
175	NOD-like receptor signaling pathway	37	66	0.4458681	1	ko04621
176	Thyroid cancer	37	73	0.7780033	1	ko05216
177	Phototransduction	37	88	0.992745	1	ko04744
178	Apoptosis	36	100	0.9999326	1	ko04210
179	Mismatch repair	34	41	0.000123267	0.003167972	ko03430
180	Systemic lupus erythematosus	34	84	0.9963506	1	ko05322
181	Linoleic acid metabolism	33	46	0.01241063	0.1139119	ko00591
182	Arachidonic acid metabolism	33	63	0.6768856	1	ko00590
183	Insect hormone biosynthesis	32	52	0.1873836	0.672261	ko00981
184	Ether lipid metabolism	32	52	0.1873836	0.672261	ko00565
185	Aldosterone-regulated sodium reabsorption	31	46	0.05166228	0.3087722	ko04960
186	Sphingolipid metabolism	31	47	0.0738916	0.3995654	ko00600
187	Acute myeloid leukemia	31	67	0.9288545	1	ko05221
188	Leishmaniasis	30	45	0.06586988	0.3761902	ko05140
189	MAPK signaling pathway - fly	30	49	0.2094763	0.7083606	ko04013
190	Melanoma	29	54	0.5987449	1	ko05218
191	Butanoate metabolism	29	55	0.6535511	1	ko00650
192	beta-Alanine metabolism	29	58	0.7921859	1	ko00410

193	SNARE interactions in vesicular transport	28	51	0.5316752	1	ko04130
194	Bladder cancer	28	52	0.5909979	1	ko05219
195	Primary immunodeficiency	28	72	0.9972175	1	ko05340
196	Valine, leucine and isoleucine degradation	28	75	0.9990261	1	ko00280
197	Circadian rhythm - mammal	27	37	0.01638908	0.1403998	ko04710
198	Propanoate metabolism	27	58	0.9098176	1	ko00640
199	Biosynthesis of unsaturated fatty acids	26	52	0.7838618	1	ko01040
200	Nicotinate and nicotinamide metabolism	25	41	0.2482449	0.7780358	ko00760
201	Alanine, aspartate and glutamate metabolism	25	63	0.9934544	1	ko00250
202	Glycosaminoglycan degradation	24	49	0.8195158	1	ko00531
203	Riboflavin metabolism	23	40	0.4112855	1	ko00740
204	Renin-angiotensin system	23	46	0.7751316	1	ko04614
205	Fatty acid metabolism	23	62	0.9979865	1	ko00071
206	Folate biosynthesis	22	29	0.01478635	0.1310377	ko00790
207	RIG-I-like receptor signaling pathway	22	45	0.815916	1	ko04622
208	Malaria	22	47	0.8846268	1	ko05144
209	Non-homologous end-joining	21	22	2.99103E-05	0.001537388	ko03450
210	Fatty acid biosynthesis	21	52	0.9852203	1	ko00061
211	Collecting duct acid secretion	21	75	0.999999	1	ko04966
212	Citrate cycle (TCA cycle)	21	93	1	1	ko00020
213	Caffeine metabolism	20	36	0.5154452	1	ko00232
214	Phenylalanine metabolism	20	38	0.6522314	1	ko00360
215	Proximal tubule bicarbonate reclamation	20	54	0.9966384	1	ko04964
216	Glyoxylate and dicarboxylate metabolism	20	59	0.9995392	1	ko00630
217	Proteasome	20	66	0.9999788	1	ko03050
218	Other glycan degradation	19	38	0.7628851	1	ko00511
219	Terpenoid backbone biosynthesis	19	56	0.9993813	1	ko00900
220	Steroid biosynthesis	18	33	0.5667992	1	ko00100
221	Type I diabetes mellitus	17	20	0.004273563	0.05780556	ko04940
222	Taste transduction	17	36	0.8504818	1	ko04742
223	Histidine metabolism	17	37	0.885611	1	ko00340
224	Glycosaminoglycan biosynthesis - chondroitin sulfate	16	33	0.8062015	1	ko00532
225	Regulation of autophagy	15	19	0.02500682	0.1785209	ko04140
226	Selenocompound metabolism	14	27	0.6799968	1	ko00450
227	Protein export	14	37	0.985953	1	ko03060
228	Sulfur relay system	13	18	0.09913723	0.4718198	ko04122
229	One carbon pool by folate	13	23	0.5063155	1	ko00670
230	Pantothenate and CoA biosynthesis	13	26	0.7436967	1	ko00770
231	Vitamin B6 metabolism	12	15	0.03867043	0.2423976	ko00750

232	Sulfur metabolism	12	22	0.5832801	1	ko00920
233	Mucin type O-Glycan biosynthesis	12	24	0.7405436	1	ko00512
234	Taurine and hypotaurine metabolism	11	13	0.02452832	0.1785209	ko00430
235	Maturity onset diabetes of the young	11	23	0.8018522	1	ko04950
236	Glycosphingolipid biosynthesis - lacto and neolacto series	11	30	0.9839136	1	ko00601
237	Autoimmune thyroid disease	10	19	0.6531286	1	ko05320
238	Glycosphingolipid biosynthesis - globo series	10	21	0.8019564	1	ko00603
239	Ubiquinone and other terpenoid-quinone biosynthesis	9	22	0.9317367	1	ko00130
240	Glycosaminoglycan biosynthesis - keratan sulfate	8	25	0.993203	1	ko00533
241	Biotin metabolism	7	9	0.1417724	0.6014731	ko00780
242	Synthesis and degradation of ketone bodies	7	11	0.3826856	1	ko00072
243	Cyanoamino acid metabolism	7	17	0.9101868	1	ko00460
244	Glycosphingolipid biosynthesis - ganglio series	5	10	0.7265991	1	ko00604
245	African trypanosomiasis	5	13	0.9245838	1	ko05143
246	Valine, leucine and isoleucine biosynthesis	5	15	0.9718857	1	ko00290
247	Butirosin and neomycin biosynthesis	5	16	0.9833213	1	ko00524
248	Lysine biosynthesis	4	4	0.08785813	0.4342219	ko00300
249	Lipoic acid metabolism	3	4	0.3819681	1	ko00785
250	Phenylalanine, tyrosine and tryptophan biosynthesis	3	7	0.8398092	1	ko00400
251	D-Arginine and D-ornithine metabolism	3	15	0.9987298	1	ko00472
252	Asthma	2	3	0.5665324	1	ko05310
253	Thiamine metabolism	2	3	0.5665324	1	ko00730
254	Primary bile acid biosynthesis	2	8	0.9804369	1	ko00120
255	Allograft rejection	1	1	0.5444699	1	ko05330
256	Graft-versus-host disease	1	1	0.5444699	1	ko05332
257	Intestinal immune network for IgA production	1	1	0.5444699	1	ko04672

Table S6a: Gene set enrichment analysis comparing 2nd and 5th instar *A. suturalis* nymphs

Category	Subcategory	Total (25962)
Cellular component	cellular process	2024
	single-organism process	1710
	metabolic process	1437
	biological regulation	1045
	multicellular organismal process	1005
	regulation of biological process	946
	developmental process	940
	response to stimulus	805
	cellular component organization or biogenesis	757
	localization	714
	signaling	588
	establishment of localization	584
	reproduction	360
	reproductive process	319
	negative regulation of biological process	316
	positive regulation of biological process	271
	locomotion	253
	growth	159
	multi-organism process	156
	biological adhesion	136
	immune system process	131
	rhythmic process	55
Biological process	cell killing	1
	cell	1541
	cell part	1541
	organelle	1059
	membrane	768
	organelle part	599
	macromolecular complex	598
	membrane part	481
	membrane-enclosed lumen	163
	extracellular region	130
	synapse	112
	cell junction	98
	synapse part	93

	extracellular region part	91
	extracellular matrix	47
	extracellular matrix part	33
	virion	1
	virion part	1
Molecular function	catalytic activity	1489
	binding	1472
	transporter activity	267
	structural molecule activity	162
	molecular transducer activity	121
	receptor activity	118
	nucleic acid binding transcription factor activity	114
	enzyme regulator activity	78
	protein binding transcription factor activity	24
	electron carrier activity	17
	antioxidant activity	12
	channel regulator activity	9
	translation regulator activity	5
	morphogen activity	3
	receptor regulator activity	2
	metallochaperone activity	1

Table S6b: Gene set pathway analysis comparing 2nd and 5th instar *A. suturalis* nymphs

	Pathway	5th/2nd (12610)	Unigenes (44419)	P-value	Q-value	Pathway ID
1	Metabolic pathways	747	2737	0.5075001	0.9723857	ko01100
2	Amoebiasis	230	617	2.5138E-08	2.10321E-06	ko05146
3	Regulation of actin cytoskeleton	230	804	0.2069426	0.5592704	ko04810
4	Focal adhesion	194	744	0.787927	0.9999998	ko04510
5	Pathways in cancer	184	613	0.0686637	0.2572327	ko05200
6	Huntington's disease	182	632	0.205787	0.5592704	ko05016
7	*Vibrio cholerae* infection	181	476	1.62665E-07	1.02073E-05	ko05110
8	RNA transport	177	703	0.908171	0.9999998	ko03013
9	Vascular smooth muscle contraction	174	557	0.02002071	0.1025551	ko04270
10	Dilated cardiomyopathy	164	458	3.35444E-05	0.000765422	ko05414
11	Protein digestion and absorption	159	345	4.11429E-14	1.03269E-11	ko04974
12	Hypertrophic cardiomyopathy (HCM)	157	450	0.000209526	0.002921724	ko05410
13	Tight junction	152	496	0.05089676	0.2094277	ko04530
14	Neuroactive ligand-receptor interaction	151	552	0.5029369	0.9723857	ko04080
15	Cardiac muscle contraction	143	331	2.56937E-10	3.22456E-08	ko04260
16	HTLV-I infection	141	434	0.008888878	0.05312163	ko05166
17	*Salmonella* infection	137	384	0.000172019	0.002539808	ko05132
18	Insulin signaling pathway	131	373	0.00049473	0.006208862	ko04910
19	Spliceosome	131	564	0.9886285	0.9999998	ko03040
20	Influenza A	127	372	0.001995799	0.01615953	ko05164
21	mRNA surveillance pathway	125	429	0.2072197	0.5592704	ko03015
22	Pancreatic secretion	124	318	3.29439E-06	0.000122081	ko04972
23	Transcriptional misregulation in cancer	124	391	0.02835821	0.1343002	ko05202
24	Purine metabolism	122	563	0.9991937	0.9999998	ko00230
25	Viral myocarditis	120	306	3.40466E-06	0.000122081	ko05416
26	Phagosome	120	398	0.108737	0.3499101	ko04145
27	Epstein-Barr virus infection	120	525	0.991765	0.9999998	ko05169
28	Herpes simplex infection	118	420	0.3722906	0.8125647	ko05168
29	ECM-receptor interaction	113	299	4.37699E-05	0.000915521	ko04512
30	Endocytosis	111	518	0.9992014	0.9999998	ko04144
31	Amyotrophic lateral sclerosis (ALS)	110	340	0.02134634	0.1071586	ko05014
32	Lysosome	108	428	0.8465452	0.9999998	ko04142
33	Ribosome	107	299	0.000736655	0.008638751	ko03010
34	Alzheimer's disease	107	378	0.3458527	0.7794076	ko05010
35	Calcium signaling pathway	107	401	0.6275691	0.9999998	ko04020

36	Ubiquitin mediated proteolysis	102	520	0.9999843	0.9999998	ko04120
37	Protein processing in endoplasmic reticulum	101	425	0.9574716	0.9999998	ko04141
38	Pathogenic *Escherichia coli* infection	99	279	0.00154949	0.01389007	ko05130
39	Bile secretion	98	530	0.9999994	0.9999998	ko04976
40	Tuberculosis	97	338	0.2978049	0.7308241	ko05152
41	Salivary secretion	93	255	0.000791599	0.008638751	ko04970
42	Parkinson's disease	93	269	0.004954229	0.03360842	ko05012
43	MAPK signaling pathway	91	345	0.6694767	0.9999998	ko04010
44	Gastric acid secretion	90	309	0.2511645	0.6499205	ko04971
45	Pyrimidine metabolism	89	411	0.9965828	0.9999998	ko00240
46	Oxidative phosphorylation	88	272	0.03608568	0.1589036	ko00190
47	Cell cycle	87	389	0.9892649	0.9999998	ko04110
48	Wnt signaling pathway	84	271	0.09615316	0.3217926	ko04310
49	Lysine degradation	76	286	0.6302855	0.9999998	ko00310
50	Chemokine signaling pathway	74	293	0.8027643	0.9999998	ko04062
51	Glutamatergic synapse	73	228	0.06382816	0.2464749	ko04724
52	Progesterone-mediated oocyte maturation	71	336	0.9963217	0.9999998	ko04914
53	Axon guidance	70	253	0.4701772	0.9219881	ko04360
54	Dorso-ventral axis formation	69	187	0.002499688	0.01960693	ko04320
55	ABC transporters	68	400	0.9999996	0.9999998	ko02010
56	Synaptic vesicle cycle	67	158	2.83823E-05	0.000712396	ko04721
57	Adherens junction	67	278	0.8992888	0.9999998	ko04520
58	Alcoholism	66	175	0.001641527	0.01395323	ko05034
59	Oocyte meiosis	66	231	0.3539939	0.7794076	ko04114
60	Peroxisome	65	223	0.288367	0.7166348	ko04146
61	Dopaminergic synapse	64	214	0.2145116	0.5727916	ko04728
62	Starch and sucrose metabolism	64	273	0.9357566	0.9999998	ko00500
63	Phosphatidylinositol signaling system	62	198	0.1165591	0.3658024	ko04070
64	Bacterial invasion of epithelial cells	62	231	0.58656	0.9999998	ko05100
65	Glycolysis / Gluconeogenesis	61	167	0.005523297	0.03554737	ko00010
66	Galactose metabolism	60	212	0.3953816	0.8482118	ko00052
67	Fc gamma R-mediated phagocytosis	60	244	0.8476237	0.9999998	ko04666
68	Vasopressin-regulated water reabsorption	60	316	0.9997882	0.9999998	ko04962
69	Glutathione metabolism	59	131	9.54114E-06	0.000299353	ko00480
70	Toxoplasmosis	59	229	0.7221363	0.9999998	ko05145
71	Melanogenesis	58	162	0.01063574	0.05932379	ko04916
72	Small cell lung cancer	57	202	0.4094523	0.8636347	ko05222
73	Shigellosis	54	185	0.3055507	0.7308241	ko05131
74	Arginine and proline metabolism	53	138	0.002857122	0.02109228	ko00330
75	Long-term potentiation	53	158	0.04837717	0.2023778	ko04720

76	Leukocyte transendothelial migration	52	234	0.9681364	0.9999998	ko04670
77	Glycerolipid metabolism	51	191	0.6002652	0.9999998	ko00561
78	Gap junction	50	123	0.000893353	0.008935121	ko04540
79	GABAergic synapse	50	137	0.01136181	0.06199596	ko04727
80	Complement and coagulation cascades	49	112	0.00012797	0.00225075	ko04610
81	Ribosome biogenesis in eukaryotes	49	258	0.9992896	0.9999998	ko03008
82	Prion diseases	48	126	0.005247405	0.03466049	ko05020
83	Glycerophospholipid metabolism	48	226	0.9851937	0.9999998	ko00564
84	Retrograde endocannabinoid signaling	47	137	0.04199254	0.1786462	ko04723
85	Inositol phosphate metabolism	47	143	0.08133015	0.2916267	ko00562
86	Arrhythmogenic right ventricular cardiomyopathy (ARVC)	47	173	0.5436762	0.9928603	ko05412
87	Drug metabolism - other enzymes	47	206	0.9389531	0.9999998	ko00983
88	Cholinergic synapse	46	119	0.004511647	0.03235495	ko04725
89	Morphine addiction	46	132	0.03383649	0.1533105	ko05032
90	Amphetamine addiction	46	144	0.1229986	0.3764957	ko05031
91	Carbohydrate digestion and absorption	46	163	0.4238933	0.8799672	ko04973
92	RNA polymerase	46	275	0.9999881	0.9999998	ko03020
93	Prostate cancer	45	181	0.793	0.9999998	ko05215
94	Endocrine and other factor-regulated calcium reabsorption	44	97	0.000100693	0.00194414	ko04961
95	Glycine, serine and threonine metabolism	44	117	0.00945445	0.0551876	ko00260
96	Neurotrophin signaling pathway	44	200	0.9638483	0.9999998	ko04722
97	Olfactory transduction	43	122	0.03258145	0.1514434	ko04740
98	Serotonergic synapse	42	111	0.009820098	0.0560192	ko04726
99	Pyruvate metabolism	42	135	0.1825395	0.5148024	ko00620
100	Amino sugar and nucleotide sugar metabolism	41	139	0.3082389	0.7308241	ko00520
101	Hepatitis C	39	136	0.3895048	0.8428078	ko05160
102	Fat digestion and absorption	39	146	0.5939062	0.9999998	ko04975
103	Cell adhesion molecules (CAMs)	38	145	0.6464059	0.9999998	ko04514
104	GnRH signaling pathway	38	157	0.8317506	0.9999998	ko04912
105	Pentose and glucuronate interconversions	38	189	0.9911905	0.9999998	ko00040
106	Measles	37	143	0.678985	0.9999998	ko05162
107	Epithelial cell signaling in *Helicobacter pylori* infection	36	122	0.3222339	0.7488955	ko05120
108	Phototransduction - fly	35	128	0.5283999	0.9928603	ko04745
109	Cocaine addiction	35	129	0.549831	0.9928603	ko05030
110	Drug metabolism - cytochrome P450	35	130	0.5709897	0.9999998	ko00982
111	Metabolism of xenobiotics by cytochrome P450	35	134	0.6518719	0.9999998	ko00980

112	Phototransduction	34	88	0.01339864	0.07006372	ko04744
113	Notch signaling pathway	34	99	0.0735822	0.2716049	ko04330
114	Fructose and mannose metabolism	34	102	0.1049684	0.346672	ko00051
115	Thyroid cancer	33	73	0.000764521	0.008638751	ko05216
116	Basal cell carcinoma	33	82	0.007406746	0.04647733	ko05217
117	Base excision repair	33	97	0.08623265	0.3006166	ko03410
118	Hedgehog signaling pathway	33	104	0.1809299	0.5148024	ko04340
119	Legionellosis	33	116	0.4242073	0.8799672	ko05134
120	Rheumatoid arthritis	33	123	0.580115	0.9999998	ko05323
121	VEGF signaling pathway	33	163	0.985024	0.9999998	ko04370
122	T cell receptor signaling pathway	33	184	0.9989114	0.9999998	ko04660
123	Citrate cycle (TCA cycle)	32	93	0.07889696	0.287002	ko00020
124	Apoptosis	32	100	0.1712305	0.4940098	ko04210
125	PPAR signaling pathway	32	142	0.9171009	0.9999998	ko03320
126	Antigen processing and presentation	32	147	0.948127	0.9999998	ko04612
127	Proximal tubule bicarbonate reclamation	31	54	3.06804E-06	0.000122081	ko04964
128	N-Glycan biosynthesis	31	84	0.03420474	0.1533105	ko00510
129	Pentose phosphate pathway	31	104	0.3156237	0.7403883	ko00030
130	TGF-beta signaling pathway	31	110	0.4527008	0.9097385	ko04350
131	ErbB signaling pathway	31	143	0.9487065	0.9999998	ko04012
132	Hematopoietic cell lineage	30	69	0.002736217	0.02081183	ko04640
133	Other types of O-glycan biosynthesis	30	114	0.62764	0.9999998	ko00514
134	Renal cell carcinoma	30	134	0.9178734	0.9999998	ko05211
135	*Staphylococcus aureus* infection	29	82	0.06677172	0.2539349	ko05150
136	Glioma	29	116	0.7424823	0.9999998	ko05214
137	Colorectal cancer	28	103	0.5472562	0.9928603	ko05210
138	Collecting duct acid secretion	27	75	0.06140773	0.2446562	ko04966
139	p53 signaling pathway	27	99	0.5399437	0.9928603	ko04115
140	Mineral absorption	27	108	0.7371154	0.9999998	ko04978
141	Cytokine-cytokine receptor interaction	27	139	0.9877973	0.9999998	ko04060
142	Basal transcription factors	27	154	0.9983585	0.9999998	ko03022
143	RNA degradation	27	167	0.9997675	0.9999998	ko03018
144	Terpenoid backbone biosynthesis	26	56	0.001667717	0.01395323	ko00900
145	Tyrosine metabolism	26	72	0.06330681	0.2464749	ko00350
146	Pertussis	26	78	0.1420828	0.4195622	ko05133
147	Jak-STAT signaling pathway	26	90	0.4054661	0.8624745	ko04630
148	Endometrial cancer	26	104	0.7343677	0.9999998	ko05213
149	Steroid hormone biosynthesis	26	110	0.8338825	0.9999998	ko00140
150	Fatty acid biosynthesis	25	52	0.001095279	0.01018204	ko00061
151	Toll-like receptor signaling pathway	25	78	0.2047885	0.5592704	ko04620

152	Chagas disease (American trypanosomiasis)	24	76	0.2354087	0.6154957	ko05142
153	Fatty acid elongation	24	78	0.2821918	0.7083014	ko00062
154	Vitamin digestion and absorption	24	96	0.7287402	0.9999998	ko04977
155	Cytosolic DNA-sensing pathway	24	129	0.9918512	0.9999998	ko04623
156	Nicotine addiction	23	69	0.1600533	0.4671323	ko05033
157	B cell receptor signaling pathway	23	83	0.5074893	0.9723857	ko04662
158	Type II diabetes mellitus	23	87	0.6114056	0.9999998	ko04930
159	Porphyrin and chlorophyll metabolism	22	103	0.9317061	0.9999998	ko00860
160	Long-term depression	21	78	0.572093	0.9999998	ko04730
161	Cysteine and methionine metabolism	21	90	0.8320913	0.9999998	ko00270
162	Adipocytokine signaling pathway	21	109	0.9799668	0.9999998	ko04920
163	NF-kappa B signaling pathway	20	94	0.9264573	0.9999998	ko04064
164	Osteoclast differentiation	20	103	0.9752073	0.9999998	ko04380
165	Retinol metabolism	20	115	0.995348	0.9999998	ko00830
166	Aldosterone-regulated sodium reabsorption	19	46	0.0277191	0.1343002	ko04960
167	Arachidonic acid metabolism	19	63	0.3489449	0.7794076	ko00590
168	Alanine, aspartate and glutamate metabolism	19	63	0.3489449	0.7794076	ko00250
169	Glycosphingolipid biosynthesis - lacto and neolacto series	18	30	0.000170567	0.002539808	ko00601
170	Glycosaminoglycan biosynthesis - chondroitin sulfate	18	33	0.000848704	0.008876027	ko00532
171	Renin-angiotensin system	18	46	0.05413691	0.2191672	ko04614
172	Glycosaminoglycan degradation	18	49	0.09496127	0.3217926	ko00531
173	MAPK signaling pathway - fly	18	49	0.09496127	0.3217926	ko04013
174	Circadian rhythm - fly	18	51	0.1307941	0.3908252	ko04711
175	Glyoxylate and dicarboxylate metabolism	18	59	0.3346175	0.7705412	ko00630
176	NOD-like receptor signaling pathway	18	66	0.5481388	0.9928603	ko04621
177	alpha-Linolenic acid metabolism	18	83	0.9007666	0.9999998	ko00592
178	Systemic lupus erythematosus	18	84	0.9112801	0.9999998	ko05322
179	Tryptophan metabolism	18	85	0.9208605	0.9999998	ko00380
180	Chronic myeloid leukemia	18	94	0.9743018	0.9999998	ko05220
181	mTOR signaling pathway	18	108	0.9966906	0.9999998	ko04150
182	Taste transduction	17	36	0.00827833	0.05067953	ko04742
183	Fc epsilon RI signaling pathway	17	74	0.8327932	0.9999998	ko04664
184	DNA replication	17	76	0.8641602	0.9999998	ko03030
185	Pancreatic cancer	17	76	0.8641602	0.9999998	ko05212
186	Nucleotide excision repair	17	87	0.9635126	0.9999998	ko03420
187	Ascorbate and aldarate metabolism	17	97	0.9911295	0.9999998	ko00053
188	Glycosaminoglycan biosynthesis - keratan sulfate	16	25	0.000134507	0.00225075	ko00533

189	SNARE interactions in vesicular transport	16	51	0.3032268	0.7308241	ko04130
190	beta-Alanine metabolism	16	58	0.5297654	0.9928603	ko00410
191	Type I diabetes mellitus	15	20	1.21981E-05	0.00034019	ko04940
192	Leishmaniasis	15	45	0.2252709	0.5951894	ko05140
193	Biosynthesis of unsaturated fatty acids	15	52	0.4524437	0.9097385	ko01040
194	Non-small cell lung cancer	15	64	0.7959043	0.9999998	ko05223
195	Acute myeloid leukemia	15	67	0.851381	0.9999998	ko05221
196	Fanconi anemia pathway	15	137	0.9999993	0.9999998	ko03460
197	Other glycan degradation	14	38	0.1282101	0.3877197	ko00511
198	Glycosylphosphatidylinositol(GPI)-anchor biosynthesis	14	55	0.6695929	0.9999998	ko00563
199	Natural killer cell mediated cytotoxicity	14	83	0.9908552	0.9999998	ko04650
200	Glycosphingolipid biosynthesis - globo series	13	21	0.00092555	0.008935121	ko00603
201	Mucin type O-Glycan biosynthesis	13	24	0.00481979	0.03360465	ko00512
202	Bladder cancer	13	52	0.6947273	0.9999998	ko05219
203	Melanoma	13	54	0.7490067	0.9999998	ko05218
204	Butanoate metabolism	13	55	0.7735872	0.9999998	ko00650
205	Propanoate metabolism	13	58	0.8370641	0.9999998	ko00640
206	Glycosaminoglycan biosynthesis - heparan sulfate	13	114	0.9999902	0.9999998	ko00534
207	Malaria	12	47	0.660508	0.9999998	ko05144
208	Valine, leucine and isoleucine degradation	12	75	0.992765	0.9999998	ko00280
209	D-Arginine and D-ornithine metabolism	11	15	0.000269672	0.003562509	ko00472
210	One carbon pool by folate	11	23	0.02826842	0.1343002	ko00670
211	Steroid biosynthesis	11	33	0.2732264	0.6989485	ko00100
212	Circadian rhythm - mammal	11	37	0.4302239	0.8851328	ko04710
213	Phenylalanine metabolism	11	38	0.4701735	0.9219881	ko00360
214	Riboflavin metabolism	11	40	0.5481446	0.9928603	ko00740
215	Linoleic acid metabolism	11	46	0.7473753	0.9999998	ko00591
216	Insect hormone biosynthesis	11	52	0.877071	0.9999998	ko00981
217	Histidine metabolism	10	37	0.5766038	0.9999998	ko00340
218	Nicotinate and nicotinamide metabolism	10	41	0.7171654	0.9999998	ko00760
219	RIG-I-like receptor signaling pathway	10	45	0.8234528	0.9999998	ko04622
220	Fatty acid metabolism	10	62	0.9867965	0.9999998	ko00071
221	Sulfur metabolism	9	22	0.1180478	0.3658024	ko00920
222	Ubiquinone and other terpenoid-quinone biosynthesis	9	22	0.1180478	0.3658024	ko00130
223	Homologous recombination	9	55	0.98042	0.9999998	ko03440
224	Maturity onset diabetes of the young	8	23	0.2756809	0.6989485	ko04950
225	Selenocompound metabolism	8	27	0.4642446	0.9219881	ko00450

226	Sphingolipid metabolism	8	47	0.9651726	0.9999998	ko00600
227	Proteasome	8	66	0.9991675	0.9999998	ko03050
228	D-Glutamine and D-glutamate metabolism	7	11	0.01259186	0.06724589	ko00471
229	Taurine and hypotaurine metabolism	7	13	0.0385426	0.1667964	ko00430
230	Vitamin B6 metabolism	7	15	0.08574772	0.3006166	ko00750
231	Pantothenate and CoA biosynthesis	7	26	0.5907998	0.9999998	ko00770
232	Folate biosynthesis	7	29	0.7148158	0.9999998	ko00790
233	Protein export	7	37	0.9126288	0.9999998	ko03060
234	Mismatch repair	7	41	0.9560628	0.9999998	ko03430
235	Cyanoamino acid metabolism	6	17	0.3086349	0.7308241	ko00460
236	Ether lipid metabolism	6	52	0.9983612	0.9999998	ko00565
237	Primary immunodeficiency	6	72	0.9999865	0.9999998	ko05340
238	Glycosphingolipid biosynthesis - ganglio series	5	10	0.1077151	0.3499101	ko00604
239	Butirosin and neomycin biosynthesis	5	16	0.453057	0.9097385	ko00524
240	Autoimmune thyroid disease	5	19	0.6239386	0.9999998	ko05320
241	Synthesis and degradation of ketone bodies	4	11	0.3513841	0.7794076	ko00072
242	Caffeine metabolism	4	36	0.9950284	0.9999998	ko00232
243	Aminoacyl-tRNA biosynthesis	4	74	0.9999998	0.9999998	ko00970
244	African trypanosomiasis	3	13	0.7323473	0.9999998	ko05143
245	Regulation of autophagy	3	19	0.9245346	0.9999998	ko04140
246	Phenylalanine, tyrosine and tryptophan biosynthesis	2	7	0.610357	0.9999998	ko00400
247	Valine, leucine and isoleucine biosynthesis	2	15	0.9444335	0.9999998	ko00290
248	Non-homologous end-joining	2	22	0.9916816	0.9999998	ko03450
249	Asthma	1	3	0.6156422	0.9999998	ko05310
250	Primary bile acid biosynthesis	1	8	0.9219346	0.9999998	ko00120
251	Sulfur relay system	1	18	0.9967844	0.9999998	ko04122

Table S7a: Gene set enrichment analysis comparing 5th instar nymphs and *A. suturalis* female adults (AFA)

Category	Subcategory	Total
Biological process	cellular process	2173
	single-organism process	1772
	metabolic process	1664
	biological regulation	1010
	multicellular organismal process	985
	developmental process	960
	regulation of biological process	922
	cellular component organization or biogenesis	759
	response to stimulus	736
	localization	598
	signaling	499
	establishment of localization	488
	reproduction	364
	reproductive process	318
	positive regulation of biological process	268
	negative regulation of biological process	261
	locomotion	206
	multi-organism process	149
	growth	143
	immune system process	130
	biological adhesion	80
	rhythmic process	42
Cellular component	cell	1623
	cell part	1623
	organelle	1215
	organelle part	719
	macromolecular complex	634
	membrane	578
	membrane part	333
	membrane-enclosed lumen	217
	extracellular region	129
	extracellular region part	78
	cell junction	43

	synapse	42
	extracellular matrix	41
	synapse part	32
	extracellular matrix part	25
	virion	1
	virion part	1
Molecular function	catalytic activity	1748
	binding	1607
	structural molecule activity	216
	transporter activity	180
	molecular transducer activity	92
	nucleic acid binding transcription factor activity	91
	receptor activity	74
	enzyme regulator activity	68
	protein binding transcription factor activity	31
	antioxidant activity	20
	electron carrier activity	18
	translation regulator activity	6
	receptor regulator activity	3
	channel regulator activity	1
	protein tag	1

Table S7b: Gene set pathway analysis comparing 5th instar nymphs and *A. suturalis* female adults (AFA)

	Pathway	AFA/5th (15450)	Unigenes (44435)	Q-value	E-value	Pathway ID
1	Metabolic pathways	885	2737	0.9412499	0.9999997	ko01100
2	Amoebiasis	312	617	9.31265E-19	1.19202E-16	ko05146
3	Regulation of actin cytoskeleton	304	804	0.0060214	0.04972511	ko04810
4	*Vibrio cholerae* infection	266	476	5.8363E-24	1.49409E-21	ko05110
5	Huntington's disease	256	632	0.000139285	0.002413061	ko05016
6	RNA transport	243	703	0.3079884	0.8130113	ko03013
7	Focal adhesion	237	744	0.8603033	0.9999997	ko04510
8	Pathways in cancer	215	613	0.2323716	0.6998486	ko05200
9	Vascular smooth muscle contraction	212	557	0.0143354	0.09657533	ko04270
10	Dilated cardiomyopathy	189	458	0.000329602	0.004440947	ko05414
11	Protein digestion and absorption	187	345	1.98263E-15	1.69185E-13	ko04974
12	HTLV-I infection	187	434	2.0812E-05	0.00056682	ko05166
13	Neuroactive ligand-receptor interaction	187	552	0.4653628	0.8886488	ko04080
14	Epstein-Barr virus infection	186	525	0.199781	0.6556915	ko05169
15	Hypertrophic cardiomyopathy (HCM)	185	450	0.000473105	0.005505216	ko05410
16	Purine metabolism	185	563	0.666647	0.9999997	ko00230
17	Tight junction	178	496	0.1508294	0.5438356	ko04530
18	Influenza A	177	372	1.3281E-08	8.49985E-07	ko05164
19	Lysosome	176	428	0.000630906	0.006729663	ko04142
20	Bile secretion	168	530	0.8408552	0.9999997	ko04976
21	*Salmonella* infection	165	384	7.35184E-05	0.001447747	ko05132
22	Herpes simplex infection	156	420	0.06871088	0.3085962	ko05168
23	Endocytosis	156	518	0.9617407	0.9999997	ko04144
24	Pyrimidine metabolism	153	411	0.06632282	0.3085962	ko00240
25	Spliceosome	151	564	0.9998428	0.9999997	ko03040
26	Phagosome	149	398	0.05863126	0.2832	ko04145
27	Cardiac muscle contraction	146	331	4.07883E-05	0.00087015	ko04260
28	Viral myocarditis	145	306	3.58498E-07	1.83551E-05	ko05416
29	Ubiquitin mediated proteolysis	144	520	0.9986154	0.9999997	ko04120
30	ABC transporters	142	400	0.2260629	0.6889536	ko02010
31	Insulin signaling pathway	139	373	0.0745838	0.3291975	ko04910
32	Pancreatic secretion	138	318	0.000161323	0.002581166	ko04972
33	Transcriptional misregulation in cancer	133	391	0.4532997	0.8886488	ko05202
34	Ribosome	131	299	0.00014139	0.002413061	ko03010
35	Cell cycle	131	389	0.5104581	0.9324515	ko04110

36	mRNA surveillance pathway	131	429	0.923133	0.9999997	ko03015
37	Pathogenic *Escherichia coli* infection	128	279	1.27959E-05	0.00040947	ko05130
38	Tuberculosis	127	338	0.06860541	0.3085962	ko05152
39	Amyotrophic lateral sclerosis (ALS)	127	340	0.07995354	0.3469171	ko05014
40	Alzheimer's disease	120	378	0.7977062	0.9999997	ko05010
41	MAPK signaling pathway	111	345	0.7352527	0.9999997	ko04010
42	Gastric acid secretion	108	309	0.3290084	0.8197656	ko04971
43	Ribosome biogenesis in eukaryotes	105	258	0.009962447	0.06924392	ko03008
44	Lysine degradation	105	286	0.1466604	0.536358	ko00310
45	Calcium signaling pathway	105	401	0.9995215	0.9999997	ko04020
46	ECM-receptor interaction	104	299	0.3551002	0.8495855	ko04512
47	Protein processing in endoplasmic reticulum	103	425	0.9999917	0.9999997	ko04141
48	Parkinson's disease	98	269	0.179336	0.6142024	ko05012
49	Adherens junction	98	278	0.3015575	0.8126181	ko04520
50	Peroxisome	92	223	0.01000791	0.06924392	ko04146
51	Salivary secretion	92	255	0.2201792	0.6791069	ko04970
52	Fc gamma R-mediated phagocytosis	91	244	0.124751	0.4769421	ko04666
53	Progesterone-mediated oocyte maturation	91	336	0.9960937	0.9999997	ko04914
54	RNA polymerase	90	275	0.6462091	0.9999997	ko03020
55	Chemokine signaling pathway	89	293	0.894752	0.9999997	ko04062
56	Wnt signaling pathway	88	271	0.6783728	0.9999997	ko04310
57	Oxidative phosphorylation	85	272	0.8153348	0.9999997	ko00190
58	Oocyte meiosis	84	231	0.2062868	0.6684737	ko04114
59	Bacterial invasion of epithelial cells	82	231	0.2939454	0.8069558	ko05100
60	Axon guidance	77	253	0.8749592	0.9999997	ko04360
61	Shigellosis	74	185	0.03995374	0.2172406	ko05131
62	Neurotrophin signaling pathway	71	200	0.310118	0.8130113	ko04722
63	Glycerophospholipid metabolism	71	226	0.7806212	0.9999997	ko00564
64	Antigen processing and presentation	69	147	0.000536997	0.005977008	ko04612
65	Glycerolipid metabolism	69	191	0.2535691	0.7376556	ko00561
66	Dopaminergic synapse	69	214	0.6902526	0.9999997	ko04728
67	Glutamatergic synapse	69	228	0.875673	0.9999997	ko04724
68	Vasopressin-regulated water reabsorption	69	316	0.9999987	0.9999997	ko04962
69	Basal transcription factors	64	154	0.02364345	0.1476274	ko03022
70	T cell receptor signaling pathway	64	184	0.3959467	0.8679914	ko04660
71	Small cell lung cancer	64	202	0.7443913	0.9999997	ko05222
72	Leukocyte transendothelial migration	63	234	0.9889267	0.9999997	ko04670
73	Alcoholism	62	175	0.3320094	0.8197656	ko05034
74	Melanogenesis	60	162	0.1996892	0.6556915	ko04916
75	Complement and coagulation cascades	59	112	2.43556E-05	0.00056682	ko04610

76	Dorso-ventral axis formation	59	187	0.7504647	0.9999997	ko04320
77	Glycine, serine and threonine metabolism	58	117	0.0002543	0.003616715	ko00260
78	Glycolysis / Gluconeogenesis	58	167	0.4089404	0.8724062	ko00010
79	RNA degradation	58	167	0.4089404	0.8724062	ko03018
80	Toxoplasmosis	58	229	0.9974689	0.9999997	ko05145
81	Arginine and proline metabolism	57	138	0.035362	0.1967972	ko00330
82	Fat digestion and absorption	57	146	0.09725682	0.4015765	ko04975
83	Phosphatidylinositol signaling system	57	198	0.9376564	0.9999997	ko04070
84	Amino sugar and nucleotide sugar metabolism	56	139	0.05859164	0.2832	ko00520
85	Prostate cancer	56	181	0.8008161	0.9999997	ko05215
86	Starch and sucrose metabolism	56	273	0.9999995	0.9999997	ko00500
87	Glycosaminoglycan biosynthesis - heparan sulfate	52	114	0.005087719	0.0434152	ko00534
88	Pyruvate metabolism	52	135	0.1324735	0.4914959	ko00620
89	Morphine addiction	51	132	0.1295309	0.4876457	ko05032
90	Fanconi anemia pathway	51	137	0.2094041	0.6700931	ko03460
91	Long-term potentiation	51	158	0.6686885	0.9999997	ko04720
92	ErbB signaling pathway	50	143	0.3970794	0.8679914	ko04012
93	Measles	50	143	0.3970794	0.8679914	ko05162
94	Base excision repair	49	97	0.00042607	0.005453701	ko03410
95	Rheumatoid arthritis	48	123	0.1204082	0.4742231	ko05323
96	Hepatitis C	48	136	0.3701405	0.8518825	ko05160
97	PPAR signaling pathway	48	142	0.5135768	0.9324515	ko03320
98	Galactose metabolism	48	212	0.9998336	0.9999997	ko00052
99	Glutathione metabolism	47	131	0.3216681	0.8197656	ko00480
100	GnRH signaling pathway	47	157	0.8572847	0.9999997	ko04912
101	*Staphylococcus aureus* infection	46	82	2.35773E-05	0.00056682	ko05150
102	Apoptosis	46	100	0.00669497	0.05355976	ko04210
103	Gap junction	46	123	0.2127028	0.6722459	ko04540
104	Cocaine addiction	46	129	0.3424255	0.8269899	ko05030
105	GABAergic synapse	46	137	0.5368708	0.9478547	ko04727
106	VEGF signaling pathway	46	163	0.9410728	0.9999997	ko04370
107	Renal cell carcinoma	45	134	0.5366411	0.9478547	ko05211
108	Amphetamine addiction	45	144	0.7544158	0.9999997	ko05031
109	Drug metabolism - other enzymes	45	206	0.9999283	0.9999997	ko00983
110	DNA replication	44	76	1.19573E-05	0.00040947	ko03030
111	Legionellosis	44	116	0.1870598	0.6300962	ko05134
112	Olfactory transduction	44	122	0.3140129	0.8130113	ko04740
113	Phototransduction - fly	44	128	0.4614448	0.8886488	ko04745
114	Synaptic vesicle cycle	43	158	0.9656425	0.9999997	ko04721
115	Nucleotide excision repair	42	87	0.003219362	0.02943417	ko03420

116	Fructose and mannose metabolism	42	102	0.06659852	0.3085962	ko00051
117	TGF-beta signaling pathway	42	110	0.1799421	0.6142024	ko04350
118	Retrograde endocannabinoid signaling	42	137	0.7953439	0.9999997	ko04723
119	Inositol phosphate metabolism	41	143	0.912435	0.9999997	ko00562
120	Arrhythmogenic right ventricular cardiomyopathy (ARVC)	41	173	0.9983305	0.9999997	ko05412
121	Vitamin digestion and absorption	39	96	0.0902122	0.3849054	ko04977
122	Prion diseases	39	126	0.7661748	0.9999997	ko05020
123	Toll-like receptor signaling pathway	38	78	0.004054886	0.03579486	ko04620
124	Jak-STAT signaling pathway	38	90	0.05449022	0.2804474	ko04630
125	Glioma	38	116	0.6126777	0.9999997	ko05214
126	Cytokine-cytokine receptor interaction	38	139	0.95395	0.9999997	ko04060
127	Cell adhesion molecules (CAMs)	38	145	0.9782668	0.9999997	ko04514
128	p53 signaling pathway	37	99	0.2447237	0.7201065	ko04115
129	Hedgehog signaling pathway	37	104	0.3711843	0.8518825	ko04340
130	Colorectal cancer	36	103	0.4237298	0.8819092	ko05210
131	Epithelial cell signaling in *Helicobacter pylori* infection	36	122	0.8560852	0.9999997	ko05120
132	Pentose and glucuronate interconversions	36	189	0.9999973	0.9999997	ko00040
133	Thyroid cancer	35	73	0.007758599	0.06018792	ko05216
134	Cytosolic DNA-sensing pathway	35	129	0.9535437	0.9999997	ko04623
135	Osteoclast differentiation	34	103	0.5893173	0.9999997	ko04380
136	Tyrosine metabolism	33	72	0.02078962	0.1330536	ko00350
137	Basal cell carcinoma	33	82	0.1248247	0.4769421	ko05217
138	N-Glycan biosynthesis	33	84	0.162115	0.5764089	ko00510
139	Citrate cycle (TCA cycle)	33	93	0.3891399	0.8679914	ko00020
140	Terpenoid backbone biosynthesis	31	56	0.000664991	0.006809505	ko00900
141	Hematopoietic cell lineage	31	69	0.0331314	0.1967972	ko04640
142	Phototransduction	31	88	0.4138174	0.8755145	ko04744
143	Endometrial cancer	31	104	0.8234055	0.9999997	ko05213
144	Pentose phosphate pathway	31	104	0.8234055	0.9999997	ko00030
145	Chronic myeloid leukemia	30	94	0.6740557	0.9999997	ko05220
146	Endocrine and other factor-regulated calcium reabsorption	30	97	0.7467517	0.9999997	ko04961
147	mTOR signaling pathway	30	108	0.9198025	0.9999997	ko04150
148	Natural killer cell mediated cytotoxicity	29	83	0.4402648	0.8886488	ko04650
149	Adipocytokine signaling pathway	29	109	0.9535086	0.9999997	ko04920
150	Pancreatic cancer	28	76	0.3144067	0.8130113	ko05212
151	alpha-Linolenic acid metabolism	28	83	0.5327378	0.9478547	ko00592
152	NF-kappa B signaling pathway	28	94	0.8149403	0.9999997	ko04064
153	Notch signaling pathway	28	99	0.892771	0.9999997	ko04330

154	Serotonergic synapse	28	111	0.9782068	0.9999997	ko04726
155	Carbohydrate digestion and absorption	28	163	0.9999992	0.9999997	ko04973
156	Glyoxylate and dicarboxylate metabolism	27	59	0.03505537	0.1967972	ko00630
157	Collecting duct acid secretion	27	75	0.3722899	0.8518825	ko04966
158	Pertussis	27	78	0.4686234	0.8886488	ko05133
159	Fatty acid elongation	27	78	0.4686234	0.8886488	ko00062
160	Other types of O-glycan biosynthesis	27	114	0.9921163	0.9999997	ko00514
161	Cholinergic synapse	27	119	0.9966433	0.9999997	ko04725
162	Homologous recombination	26	55	0.02454939	0.1496344	ko03440
163	Fc epsilon RI signaling pathway	26	74	0.4342723	0.8886488	ko04664
164	Nicotine addiction	25	69	0.3654699	0.8518825	ko05033
165	Primary immunodeficiency	25	72	0.4656263	0.8886488	ko05340
166	Steroid hormone biosynthesis	25	110	0.9953887	0.9999997	ko00140
167	Fatty acid biosynthesis	24	52	0.04073262	0.2172406	ko00061
168	Non-small cell lung cancer	24	64	0.2963041	0.8069558	ko05223
169	B cell receptor signaling pathway	24	83	0.8478298	0.9999997	ko04662
170	Cysteine and methionine metabolism	24	90	0.9370868	0.9999997	ko00270
171	Mismatch repair	23	41	0.002543094	0.0241123	ko03430
172	Circadian rhythm - fly	23	51	0.05828419	0.2832	ko04711
173	Proximal tubule bicarbonate reclamation	23	54	0.1064173	0.4256692	ko04964
174	NOD-like receptor signaling pathway	23	66	0.4623103	0.8886488	ko04621
175	Acute myeloid leukemia	23	67	0.4971986	0.9157039	ko05221
176	Tryptophan metabolism	23	85	0.9212063	0.9999997	ko00380
177	Chagas disease (American trypanosomiasis)	22	76	0.8379342	0.9999997	ko05142
178	Type II diabetes mellitus	21	87	0.9791705	0.9999997	ko04930
179	Mineral absorption	21	108	0.999633	0.9999997	ko04978
180	Drug metabolism - cytochrome P450	21	130	0.9999978	0.9999997	ko00982
181	Glycosphingolipid biosynthesis - lacto and neolacto series	20	30	0.000218961	0.0032973	ko00601
182	Circadian rhythm - mammal	20	37	0.00828238	0.06236145	ko04710
183	Renin-angiotensin system	20	46	0.1051954	0.4256692	ko04614
184	Biosynthesis of unsaturated fatty acids	20	52	0.2734497	0.7777367	ko01040
185	Metabolism of xenobiotics by cytochrome P450	20	134	0.9999997	0.9999997	ko00980
186	Ether lipid metabolism	19	52	0.3772134	0.854572	ko00565
187	Arachidonic acid metabolism	19	63	0.7608321	0.9999997	ko00590
188	Alanine, aspartate and glutamate metabolism	19	63	0.7608321	0.9999997	ko00250
189	Systemic lupus erythematosus	19	84	0.9898947	0.9999997	ko05322
190	Aldosterone-regulated sodium reabsorption	18	46	0.2594817	0.7463743	ko04960
191	Glycosaminoglycan degradation	18	49	0.3726986	0.8518825	ko00531
192	Insect hormone biosynthesis	18	52	0.4917077	0.9121534	ko00981
193	Bladder cancer	18	52	0.4917077	0.9121534	ko05219

194	Melanoma	18	54	0.5689899	0.9976809	ko05218
195	Glycosphingolipid biosynthesis - globo series	17	21	1.15683E-05	0.00040947	ko00603
196	Glycosaminoglycan biosynthesis - keratan sulfate	17	25	0.000462155	0.005505216	ko00533
197	Fatty acid metabolism	17	62	0.8803886	0.9999997	ko00071
198	Long-term depression	17	78	0.9921002	0.9999997	ko04730
199	Retinol metabolism	17	115	0.9999987	0.9999997	ko00830
200	Glycosaminoglycan biosynthesis - chondroitin sulfate	16	33	0.05477489	0.2804474	ko00532
201	Nicotinate and nicotinamide metabolism	15	41	0.4000898	0.8679914	ko00760
202	SNARE interactions in vesicular transport	15	51	0.7820494	0.9999997	ko04130
203	Propanoate metabolism	15	58	0.9207046	0.9999997	ko00640
204	Ascorbate and aldarate metabolism	15	97	0.9999843	0.9999997	ko00053
205	Porphyrin and chlorophyll metabolism	15	103	0.9999968	0.9999997	ko00860
206	Riboflavin metabolism	14	40	0.4858864	0.9121534	ko00740
207	Linoleic acid metabolism	14	46	0.7267506	0.9999997	ko00591
208	Malaria	14	47	0.7589187	0.9999997	ko05144
209	Aminoacyl-tRNA biosynthesis	14	74	0.9983494	0.9999997	ko00970
210	One carbon pool by folate	13	23	0.02002518	0.1314473	ko00670
211	Other glycan degradation	13	38	0.5303428	0.9478547	ko00511
212	RIG-I-like receptor signaling pathway	13	45	0.7953588	0.9999997	ko04622
213	MAPK signaling pathway - fly	13	49	0.8874133	0.9999997	ko04013
214	beta-Alanine metabolism	13	58	0.9775334	0.9999997	ko00410
215	Valine, leucine and isoleucine degradation	13	75	0.999495	0.9999997	ko00280
216	Non-homologous end-joining	12	22	0.03488188	0.1967972	ko03450
217	Folate biosynthesis	12	29	0.2423891	0.7201065	ko00790
218	Steroid biosynthesis	12	33	0.4330575	0.8886488	ko00100
219	Histidine metabolism	12	37	0.6215929	0.9999997	ko00340
220	Leishmaniasis	12	45	0.8757032	0.9999997	ko05140
221	Sphingolipid metabolism	12	47	0.9109327	0.9999997	ko00600
222	D-Arginine and D-ornithine metabolism	11	15	0.001950526	0.01920518	ko00472
223	Selenocompound metabolism	11	27	0.2764611	0.7777367	ko00450
224	Phenylalanine metabolism	11	38	0.780683	0.9999997	ko00360
225	Glycosylphosphatidylinositol(GPI)-anchor biosynthesis	11	55	0.991277	0.9999997	ko00563
226	Proteasome	11	66	0.9993851	0.9999997	ko03050
227	Sulfur metabolism	10	22	0.1704492	0.5977397	ko00920
228	Butanoate metabolism	10	55	0.9965256	0.9999997	ko00650
229	Mucin type O-Glycan biosynthesis	9	24	0.4174022	0.8758604	ko00512
230	Taste transduction	9	36	0.9011646	0.9999997	ko04742
231	D-Glutamine and D-glutamate metabolism	8	11	0.009336357	0.06828878	ko00471

232	Vitamin B6 metabolism	8	15	0.09220127	0.386943	ko00750
233	Autoimmune thyroid disease	8	19	0.2885816	0.8030097	ko05320
234	Caffeine metabolism	8	36	0.9524421	0.9999997	ko00232
235	Pantothenate and CoA biosynthesis	6	26	0.9149584	0.9999997	ko00770
236	Glycosphingolipid biosynthesis - ganglio series	5	10	0.2190518	0.6791069	ko00604
237	Taurine and hypotaurine metabolism	5	13	0.4569488	0.8886488	ko00430
238	African trypanosomiasis	5	13	0.4569488	0.8886488	ko05143
239	Cyanoamino acid metabolism	5	17	0.7271658	0.9999997	ko00460
240	Type I diabetes mellitus	5	20	0.8548602	0.9999997	ko04940
241	Protein export	5	37	0.9985703	0.9999997	ko03060
242	Ubiquinone and other terpenoid-quinone biosynthesis	4	22	0.9670928	0.9999997	ko00130
243	Maturity onset diabetes of the young	4	23	0.9752563	0.9999997	ko04950
244	Valine, leucine and isoleucine biosynthesis	3	15	0.9240524	0.9999997	ko00290
245	Butirosin and neomycin biosynthesis	3	16	0.9434243	0.9999997	ko00524
246	Sulfur relay system	3	18	0.9691675	0.9999997	ko04122
247	Phenylalanine, tyrosine and tryptophan biosynthesis	2	7	0.741983	0.9999997	ko00400
248	Primary bile acid biosynthesis	2	8	0.8096737	0.9999997	ko00120
249	Biotin metabolism	2	9	0.8610205	0.9999997	ko00780
250	Synthesis and degradation of ketone bodies	2	11	0.9276332	0.9999997	ko00072
251	Regulation of autophagy	2	19	0.9956024	0.9999997	ko04140
252	Allograft rejection	1	1	0.336232	0.8197656	ko05330
253	Graft-versus-host disease	1	1	0.336232	0.8197656	ko05332
254	Intestinal immune network for IgA production	1	1	0.336232	0.8197656	ko04672
255	Thiamine metabolism	1	3	0.707575	0.9999997	ko00730
256	Lysine biosynthesis	1	4	0.805913	0.9999997	ko00300

Table S8a: Gene set enrichment analysis comparing 5th instar nymphs and *A. suturalis* male adults (AMA)

Category	Subcategory	Total(13431)
Biological process	cellular process	1091
	metabolic process	890
	single-organism process	881
	multicellular organismal process	523
	developmental process	497
	biological regulation	493
	regulation of biological process	445
	response to stimulus	383
	cellular component organization or biogenesis	380
	localization	284
	signaling	234
	establishment of localization	226
	reproduction	199
	reproductive process	172
	positive regulation of biological process	132
	negative regulation of biological process	127
	locomotion	111
	multi-organism process	101
	immune system process	81
	growth	57
	biological adhesion	48
	rhythmic process	19
	cell killing	2
Cellular component	cell	797
	cell part	797
	organelle	583
	organelle part	358
	macromolecular complex	341
	membrane	321
	membrane part	186
	extracellular region	110
	membrane-enclosed lumen	107
	extracellular region part	66
	extracellular matrix	42
	extracellular matrix part	26

	cell junction	23
	synapse	21
	synapse part	16
	nucleoid	1
	virion	1
	virion part	1
Molecular function	catalytic activity	868
	binding	833
	structural molecule activity	201
	transporter activity	103
	nucleic acid binding transcription factor activity	59
	molecular transducer activity	55
	receptor activity	55
	enzyme regulator activity	35
	electron carrier activity	16
	antioxidant activity	15
	protein binding transcription factor activity	9
	translation regulator activity	5
	channel regulator activity	2
	receptor regulator activity	2

Table S8b: Gene set pathway analysis comparing 5th instar nymphs and *A. suturalis* male adults (AMA)

	Pathway	AMA/5th (8382)	Unigenes (44390)	P-value	Q-value	Pathway ID
1	Metabolic pathways	524	2737	0.01657234	0.1044057	ko01100
2	Amoebiasis	282	617	2.69647E-60	6.79511E-58	ko05146
3	*Vibrio cholerae* infection	212	476	3.95653E-43	4.98523E-41	ko05110
4	Focal adhesion	158	744	0.006261249	0.05568799	ko04510
5	Epstein-barr virus infection	124	525	0.000290297	0.003850251	ko05169
6	Ribosome	121	299	1.0089E-20	8.47477E-19	ko03010
7	Pathways in cancer	121	613	0.09710424	0.3823479	ko05200
8	Protein digestion and absorption	119	345	3.10355E-14	1.95524E-12	ko04974
9	Huntington's disease	118	632	0.2681064	0.7128799	ko05016
10	Lysosome	117	428	3.63642E-07	1.30911E-05	ko04142
11	Bile secretion	116	530	0.006920971	0.0562608	ko04976
12	Purine metabolism	113	563	0.07456819	0.3416579	ko00230
13	Regulation of actin cytoskeleton	112	804	0.9985133	0.9999999	ko04810
14	RNA transport	109	703	0.945874	0.9999999	ko03013
15	ECM-receptor interaction	104	299	7.14087E-13	3.599E-11	ko04512
16	Spliceosome	98	564	0.5934987	0.9999999	ko03040
17	Pyrimidine metabolism	95	411	0.002797251	0.02773654	ko00240
18	Neuroactive ligand-receptor interaction	93	552	0.714982	0.9999999	ko04080
19	Herpes simplex infection	89	420	0.03483697	0.1828941	ko05168
20	Hypertrophic cardiomyopathy (HCM)	87	450	0.1916481	0.6036915	ko05410
21	ABC transporters	84	400	0.04742673	0.2390307	ko02010
22	Pathogenic *Escherichia coli* infection	82	279	9.21343E-07	2.23773E-05	ko05130
23	Transcriptional misregulation in cancer	82	391	0.05094444	0.2443484	ko05202
24	Dilated cardiomyopathy	82	458	0.4686698	0.9999999	ko05414
25	Phagosome	77	398	0.206164	0.6259437	ko04145
26	Pancreatic secretion	76	318	0.002861706	0.02773654	ko04972
27	RNA polymerase	74	275	8.35551E-05	0.001315993	ko03020
28	Protein processing in endoplasmic reticulum	74	425	0.5782276	0.9999999	ko04141
29	HTLV-I infection	74	434	0.6552217	0.9999999	ko05166
30	Influenza A	73	372	0.1770458	0.5870466	ko05164
31	mRNA surveillance pathway	66	429	0.908993	0.9999999	ko03015

32	Tight junction	66	496	0.9968288	0.9999999	ko04530
33	Ubiquitin mediated proteolysis	66	520	0.9993075	0.9999999	ko04120
34	Cell cycle	64	389	0.7582194	0.9999999	ko04110
35	Endocytosis	62	518	0.99988	0.9999999	ko04144
36	Vascular smooth muscle contraction	62	557	0.9999945	0.9999999	ko04270
37	Salivary secretion	59	255	0.01543715	0.09974774	ko04970
38	MAPK signaling pathway	58	345	0.6864741	0.9999999	ko04010
39	Alzheimer's disease	57	378	0.922384	0.9999999	ko05010
40	Glycerolipid metabolism	55	191	9.83144E-05	0.001457366	ko00561
41	Tuberculosis	55	338	0.773162	0.9999999	ko05152
42	Glycerophospholipid metabolism	54	226	0.01064547	0.07968944	ko00564
43	Adherens junction	54	278	0.2425566	0.6731333	ko04520
44	Small cell lung cancer	53	202	0.001469242	0.0176309	ko05222
45	Insulin signaling pathway	53	373	0.969983	0.9999999	ko04910
46	Lysine degradation	52	286	0.4354131	0.9710097	ko00310
47	Antigen processing and presentation	51	147	5.3096E-07	1.53206E-05	ko04612
48	Fat digestion and absorption	50	146	1.06232E-06	2.23773E-05	ko04975
49	Cardiac muscle contraction	48	331	0.9481973	0.9999999	ko04260
50	Amyotrophic lateral sclerosis (ALS)	48	340	0.9675965	0.9999999	ko05014
51	Parkinson's disease	47	269	0.5609445	0.9999999	ko05012
52	Viral myocarditis	47	306	0.8756063	0.9999999	ko05416
53	Gastric acid secretion	47	309	0.8907503	0.9999999	ko04971
54	Progesterone-mediated oocyte maturation	47	336	0.9714823	0.9999999	ko04914
55	*Salmonella* infection	47	384	0.998702	0.9999999	ko05132
56	Peroxisome	46	223	0.142117	0.5105191	ko04146
57	Toxoplasmosis	46	229	0.1897956	0.6036915	ko05145
58	Oxidative phosphorylation	44	272	0.7661658	0.9999999	ko00190
59	Fc gamma R-mediated phagocytosis	43	244	0.5357462	0.9999999	ko04666
60	*Staphylococcus aureus* infection	42	82	4.66344E-12	1.95865E-10	ko05150
61	Axon guidance	42	253	0.6994008	0.9999999	ko04360
62	Chemokine signaling pathway	41	293	0.9625438	0.9999999	ko04062
63	Calcium signaling pathway	40	401	0.9999955	0.9999999	ko04020
64	Starch and sucrose metabolism	39	273	0.9437643	0.9999999	ko00500
65	Complement and coagulation cascades	38	112	2.52948E-05	0.000424953	ko04610
66	Vitamin digestion and absorption	37	96	1.06559E-06	2.23773E-05	ko04977
67	Shigellosis	37	185	0.2283527	0.6731333	ko05131
68	Glycosaminoglycan biosynthesis - heparan sulfate	36	114	0.00021444	0.003002164	ko00534
69	Amino sugar and nucleotide sugar	36	139	0.009582889	0.07546525	ko00520

	metabolism					
70	PPAR signaling pathway	36	142	0.01345933	0.09166895	ko03320
71	Prostate cancer	36	181	0.2430759	0.6731333	ko05215
72	Ribosome biogenesis in eukaryotes	34	258	0.9797813	0.9999999	ko03008
73	Wnt signaling pathway	34	271	0.991737	0.9999999	ko04310
74	Drug metabolism - other enzymes	33	206	0.7611298	0.9999999	ko00983
75	Bacterial invasion of epithelial cells	33	231	0.929159	0.9999999	ko05100
76	Leukocyte transendothelial migration	33	234	0.9402367	0.9999999	ko04670
77	Vasopressin-regulated water reabsorption	33	316	0.9998884	0.9999999	ko04962
78	Pentose and glucuronate interconversions	32	189	0.6363146	0.9999999	ko00040
79	Fatty acid elongation	31	78	3.6644E-06	7.10329E-05	ko00062
80	Cell adhesion molecules (CAMs)	31	145	0.1442641	0.5105191	ko04514
81	Arrhythmogenic right ventricular cardiomyopathy (ARVC)	31	173	0.4982159	0.9999999	ko05412
82	Hematopoietic cell lineage	30	69	5.47162E-07	1.53206E-05	ko04640
83	Basal transcription factors	30	154	0.3087018	0.7555224	ko03022
84	T cell receptor signaling pathway	30	184	0.7171381	0.9999999	ko04660
85	Tyrosine metabolism	29	72	5.46641E-06	9.83954E-05	ko00350
86	Morphine addiction	29	132	0.1200903	0.4516829	ko05032
87	Measles	29	143	0.2357566	0.6731333	ko05162
88	Steroid hormone biosynthesis	28	110	0.02551464	0.1397759	ko00140
89	Alcoholism	28	175	0.7494823	0.9999999	ko05034
90	Dopaminergic synapse	28	214	0.9723698	0.9999999	ko04728
91	Tryptophan metabolism	27	85	0.001135087	0.0143021	ko00380
92	Glycine, serine and threonine metabolism	27	117	0.08193828	0.3522636	ko00260
93	Prion diseases	27	126	0.1607767	0.55501	ko05020
94	Drug metabolism - cytochrome P450	27	130	0.2060774	0.6259437	ko00982
95	Hepatitis C	27	136	0.2841096	0.7380992	ko05160
96	Retrograde endocannabinoid signaling	27	137	0.2980867	0.7511785	ko04723
97	Arginine and proline metabolism	27	138	0.3122904	0.7567037	ko00330
98	Cytokine-cytokine receptor interaction	27	139	0.3267	0.78408	ko04060
99	Galactose metabolism	27	212	0.980024	0.9999999	ko00052
100	alpha-Linolenic acid metabolism	26	83	0.001737619	0.01990364	ko00592
101	Rheumatoid arthritis	26	123	0.1850829	0.5979601	ko05323

102	GABAergic synapse	26	137	0.3784735	0.8831048	ko04727
103	Dorso-ventral axis formation	26	187	0.9309138	0.9999999	ko04320
104	Metabolism of xenobiotics by cytochrome P450	25	134	0.4178228	0.9485707	ko00980
105	Phosphatidylinositol signaling system	25	198	0.979046	0.9999999	ko04070
106	Neurotrophin signaling pathway	24	200	0.989329	0.9999999	ko04722
107	Cysteine and methionine metabolism	23	90	0.03845576	0.1977725	ko00270
108	Base excision repair	23	97	0.08021174	0.3522636	ko03410
109	p53 signaling pathway	23	99	0.09626898	0.3823479	ko04115
110	Retinol metabolism	23	115	0.2910147	0.7455219	ko00830
111	Fanconi anemia pathway	23	137	0.6427094	0.9999999	ko03460
112	Melanogenesis	23	162	0.9007154	0.9999999	ko04916
113	Jak-STAT signaling pathway	22	90	0.06499354	0.3033032	ko04630
114	Ascorbate and aldarate metabolism	22	97	0.1243261	0.4607379	ko00053
115	Notch signaling pathway	22	99	0.1458626	0.5105191	ko04330
116	Legionellosis	22	116	0.394854	0.9128735	ko05134
117	Glutamatergic synapse	22	228	0.9997843	0.9999999	ko04724
118	Nicotine addiction	21	69	0.006662619	0.055966	ko05033
119	DNA replication	21	76	0.02072911	0.1274082	ko03030
120	Phototransduction	21	88	0.08658626	0.3636623	ko04744
121	Osteoclast differentiation	21	103	0.2705877	0.7128799	ko04380
122	Olfactory transduction	21	122	0.5909844	0.9999999	ko04740
123	Cytosolic DNA-sensing pathway	21	129	0.6969846	0.9999999	ko04623
124	Cocaine addiction	21	129	0.6969846	0.9999999	ko05030
125	Renal cell carcinoma	21	134	0.7620915	0.9999999	ko05211
126	Pyruvate metabolism	21	135	0.7739363	0.9999999	ko00620
127	RNA degradation	21	167	0.97128	0.9999999	ko03018
128	Oocyte meiosis	21	231	0.9999301	0.9999999	ko04114
129	Primary immunodeficiency	20	72	0.02233239	0.1297454	ko05340
130	Glutathione metabolism	20	131	0.7978062	0.9999999	ko00480
131	VEGF signaling pathway	19	163	0.9868603	0.9999999	ko04370
132	Aminoacyl-tRNA biosynthesis	18	74	0.09216423	0.380744	ko00970
133	Endometrial cancer	18	104	0.5799265	0.9999999	ko05213
134	Glycolysis / Gluconeogenesis	18	167	0.9951483	0.9999999	ko00010
135	Insect hormone biosynthesis	17	52	0.006408539	0.05568799	ko00981
136	Biosynthesis of unsaturated fatty acids	17	52	0.006408539	0.05568799	ko01040
137	Glyoxylate and dicarboxylate metabolism	17	59	0.02375328	0.1330184	ko00630

138	Thyroid cancer	17	73	0.1356214	0.4953129	ko05216
139	Citrate cycle (TCA cycle)	17	93	0.4819231	0.9999999	ko00020
140	NF-kappa B signaling pathway	17	94	0.5011045	0.9999999	ko04064
141	Porphyrin and chlorophyll metabolism	17	103	0.6626396	0.9999999	ko00860
142	TGF-beta signaling pathway	17	110	0.7661182	0.9999999	ko04350
143	Serotonergic synapse	17	111	0.7789806	0.9999999	ko04726
144	Other types of O-glycan biosynthesis	17	114	0.8146173	0.9999999	ko00514
145	Inositol phosphate metabolism	17	143	0.9778084	0.9999999	ko00562
146	Circadian rhythm - fly	16	51	0.01230701	0.08614907	ko04711
147	Fatty acid biosynthesis	16	52	0.01492855	0.09899986	ko00061
148	Valine, leucine and isoleucine degradation	16	75	0.2428829	0.6731333	ko00280
149	Glioma	16	116	0.8921692	0.9999999	ko05214
150	Gap junction	16	123	0.9353824	0.9999999	ko04540
151	ErbB signaling pathway	16	143	0.9880433	0.9999999	ko04012
152	Synaptic vesicle cycle	16	158	0.9971957	0.9999999	ko04721
153	beta-Alanine metabolism	15	58	0.07624146	0.3430866	ko00410
154	Toll-like receptor signaling pathway	15	78	0.4045355	0.9267541	ko04620
155	Systemic lupus erythematosus	15	84	0.5277205	0.9999999	ko05322
156	Chronic myeloid leukemia	15	94	0.7101766	0.9999999	ko05220
157	Amphetamine addiction	15	144	0.994547	0.9999999	ko05031
158	Carbohydrate digestion and absorption	15	163	0.9992588	0.9999999	ko04973
159	Fatty acid metabolism	14	62	0.1955727	0.6084484	ko00071
160	Collecting duct acid secretion	14	75	0.4579809	0.9999999	ko04966
161	Apoptosis	14	100	0.8656297	0.9999999	ko04210
162	Pentose phosphate pathway	14	104	0.8995762	0.9999999	ko00030
163	mTOR signaling pathway	14	108	0.9261574	0.9999999	ko04150
164	Other glycan degradation	13	38	0.01075175	0.07968944	ko00511
165	Linoleic acid metabolism	13	46	0.05139073	0.2443484	ko00591
166	Basal cell carcinoma	13	82	0.7114801	0.9999999	ko05217
167	Colorectal cancer	13	103	0.9356066	0.9999999	ko05210
168	Hedgehog signaling pathway	13	104	0.9407539	0.9999999	ko04340
169	Histidine metabolism	12	37	0.02169093	0.1297454	ko00340
170	Phenylalanine metabolism	12	38	0.02673978	0.1433707	ko00360
171	Renin-angiotensin system	12	46	0.09996733	0.3875656	ko04614
172	Melanoma	12	54	0.236522	0.6731333	ko05218
173	Proximal tubule bicarbonate reclamation	12	54	0.236522	0.6731333	ko04964

174	Butanoate metabolism	12	55	0.257308	0.6972217	ko00650
175	B cell receptor signaling pathway	12	83	0.8187393	0.9999999	ko04662
176	Mineral absorption	12	108	0.977941	0.9999999	ko04978
177	Adipocytokine signaling pathway	12	109	0.9799763	0.9999999	ko04920
178	Phototransduction - fly	12	128	0.997249	0.9999999	ko04745
179	Circadian rhythm - mammal	11	37	0.05028766	0.2443484	ko04710
180	Riboflavin metabolism	11	40	0.08247441	0.3522636	ko00740
181	Glycosaminoglycan degradation	11	49	0.238814	0.6731333	ko00531
182	Chagas disease (American trypanosomiasis)	11	76	0.8099873	0.9999999	ko05142
183	Type II diabetes mellitus	11	87	0.920468	0.9999999	ko04930
184	Epithelial cell signaling in *Helicobacter pylori* infection	11	122	0.9977471	0.9999999	ko05120
185	GnRH signaling pathway	11	157	0.9999713	0.9999999	ko04912
186	Long-term potentiation	11	158	0.9999749	0.9999999	ko04720
187	Folate biosynthesis	10	29	0.02265396	0.1297454	ko00790
188	Bladder cancer	10	52	0.4401741	0.9730164	ko05219
189	Arachidonic acid metabolism	10	63	0.696868	0.9999999	ko00590
190	Non-small cell lung cancer	10	64	0.7162254	0.9999999	ko05223
191	Acute myeloid leukemia	10	67	0.7693655	0.9999999	ko05221
192	Pancreatic cancer	10	76	0.8854747	0.9999999	ko05212
193	Long-term depression	10	78	0.9033896	0.9999999	ko04730
194	Fructose and mannose metabolism	10	102	0.9910065	0.9999999	ko00051
195	Autoimmune thyroid disease	9	19	0.002788689	0.02773654	ko05320
196	Steroid biosynthesis	9	33	0.1147142	0.4379997	ko00100
197	Nicotinate and nicotinamide metabolism	9	41	0.2928836	0.7455219	ko00760
198	Propanoate metabolism	9	58	0.7189716	0.9999999	ko00640
199	Natural killer cell mediated cytotoxicity	9	83	0.9690582	0.9999999	ko04650
200	Cholinergic synapse	9	119	0.9995573	0.9999999	ko04725
201	Vitamin B6 metabolism	8	15	0.001864738	0.02043104	ko00750
202	RIG-I-like receptor signaling pathway	8	45	0.5541396	0.9999999	ko04622
203	Aldosterone-regulated sodium reabsorption	8	46	0.5808353	0.9999999	ko04960
204	MAPK signaling pathway - fly	8	49	0.65599	0.9999999	ko04013
205	Fc epsilon RI signaling pathway	8	74	0.9629537	0.9999999	ko04664
206	Pertussis	8	78	0.9759405	0.9999999	ko05133
207	One carbon pool by folate	7	23	0.09664343	0.3823479	ko00670
208	Sphingolipid metabolism	7	47	0.7485209	0.9999999	ko00600

209	Homologous recombination	7	55	0.8763368	0.9999999	ko03440
210	Terpenoid backbone biosynthesis	7	56	0.8876696	0.9999999	ko00900
211	Nucleotide excision repair	7	87	0.9968215	0.9999999	ko03420
212	Sulfur metabolism	6	22	0.1800664	0.5893082	ko00920
213	Pantothenate and CoA biosynthesis	6	26	0.3058481	0.7555224	ko00770
214	Caffeine metabolism	6	36	0.6312434	0.9999999	ko00232
215	Protein export	6	37	0.6589309	0.9999999	ko03060
216	Mismatch repair	6	41	0.7560403	0.9999999	ko03430
217	Malaria	6	47	0.861141	0.9999999	ko05144
218	Alanine, aspartate and glutamate metabolism	6	63	0.9765016	0.9999999	ko00250
219	Biotin metabolism	5	9	0.01154265	0.08310708	ko00780
220	Type I diabetes mellitus	5	20	0.2715733	0.7128799	ko04940
221	Glycosphingolipid biosynthesis - globo series	5	21	0.3088048	0.7555224	ko00603
222	Non-homologous end-joining	5	22	0.346669	0.8241565	ko03450
223	Ether lipid metabolism	5	52	0.9650877	0.9999999	ko00565
224	Glycosylphosphatidylinositol(GPI)-anchor biosynthesis	5	55	0.9760937	0.9999999	ko00563
225	Ubiquinone and other terpenoid-quinone biosynthesis	4	22	0.5621896	0.9999999	ko00130
226	Selenocompound metabolism	4	27	0.7282477	0.9999999	ko00450
227	Glycosaminoglycan biosynthesis - chondroitin sulfate	4	33	0.8593585	0.9999999	ko00532
228	N-Glycan biosynthesis	4	84	0.9999124	0.9999999	ko00510
229	Glycosphingolipid biosynthesis - ganglio series	3	10	0.2534651	0.694274	ko00604
230	Maturity onset diabetes of the young	3	23	0.7994977	0.9999999	ko04950
231	Mucin type O-Glycan biosynthesis	3	24	0.8230113	0.9999999	ko00512
232	Glycosphingolipid biosynthesis - lacto and neolacto series	3	30	0.9198512	0.9999999	ko00601
233	Leishmaniasis	3	45	0.9911699	0.9999999	ko05140
234	SNARE interactions in vesicular transport	3	51	0.996556	0.9999999	ko04130
235	NOD-like receptor signaling pathway	3	66	0.9997022	0.9999999	ko04621
236	Proteasome	3	66	0.9997022	0.9999999	ko03050
237	Phenylalanine, tyrosine and tryptophan biosynthesis	2	7	0.3586554	0.8446837	ko00400
238	Primary bile acid biosynthesis	2	8	0.426758	0.9602055	ko00120
239	Taurine and hypotaurine metabolism	2	13	0.6977472	0.9999999	ko00430

240	African trypanosomiasis	2	13	0.6977472	0.9999999	ko05143
241	Valine, leucine and isoleucine biosynthesis	2	15	0.7720095	0.9999999	ko00290
242	Butirosin and neomycin biosynthesis	2	16	0.8027883	0.9999999	ko00524
243	Cyanoamino acid metabolism	2	17	0.8298135	0.9999999	ko00460
244	Taste transduction	2	36	0.9921057	0.9999999	ko04742
245	Endocrine and other factor-regulated calcium reabsorption	2	97	0.9999999	0.9999999	ko04961
246	Allograft rejection	1	1	0.1767861	0.5870466	ko05330
247	Graft-versus-host disease	1	1	0.1767861	0.5870466	ko05332
248	Lipoic acid metabolism	1	4	0.5407791	0.9999999	ko00785
249	Lysine biosynthesis	1	4	0.5407791	0.9999999	ko00300
250	Synthesis and degradation of ketone bodies	1	11	0.8824094	0.9999999	ko00072
251	Sulfur relay system	1	18	0.9699056	0.9999999	ko04122
252	Glycosaminoglycan biosynthesis - keratan sulfate	1	25	0.9923023	0.9999999	ko00533

Table S9a: Gene set enrichment analysis comparing *A. suturalis* male and female adults

Category	Subcategory	Total (23474)
Biological process	cellular process	2027
	single-organism process	1645
	metabolic process	1472
	biological regulation	971
	regulation of biological process	901
	multicellular organismal process	900
	developmental process	845
	response to stimulus	708
	cellular component organization or biogenesis	697
	localization	518
	signaling	477
	establishment of localization	411
	reproduction	309
	positive regulation of biological process	267
	reproductive process	265
	negative regulation of biological process	258
	locomotion	211
	growth	117
	immune system process	117
	multi-organism process	108
	biological adhesion	62
	rhythmic process	43
Cellular component	cell	1502
	cell part	1502
	organelle	1120
	organelle part	670
	macromolecular complex	567
	membrane	481
	membrane part	248
	membrane-enclosed lumen	228
	extracellular region	55
	synapse	50
	cell junction	45
	extracellular region part	45
	synapse part	37
	extracellular matrix	14

	extracellular matrix part	6
	nucleoid	1
Molecular function	catalytic activity	1568
	binding	1459
	transporter activity	142
	nucleic acid binding transcription factor activity	86
	molecular transducer activity	77
	enzyme regulator activity	66
	structural molecule activity	61
	receptor activity	57
	protein binding transcription factor activity	34
	antioxidant activity	11
	electron carrier activity	4
	translation regulator activity	4
	receptor regulator activity	2
	channel regulator activity	1
	morphogen activity	1
	protein tag	1

Table S9b: Gene set pathway analysis comparing *A. suturalis* adult males and females

	Pathway	AMA/AFA (12367)	Unigenes (44382)	P-value	Q-value	Pathway ID
1	Metabolic pathways	701	2737	0.9999981	1	ko01100
2	Regulation of actin cytoskeleton	245	804	0.2352117	0.9074972	ko04810
3	RNA transport	215	703	0.2315706	0.9074972	ko03013
4	Huntington's disease	190	632	0.34451	1	ko05016
5	Vascular smooth muscle contraction	178	557	0.0874533	0.4390156	ko04270
6	Focal adhesion	174	744	0.9998993	1	ko04510
7	HTLV-I infection	165	434	4.72285E-05	0.001185435	ko05166
8	Amoebiasis	157	617	0.985962	1	ko05146
9	Pathways in cancer	155	613	0.988705	1	ko05200
10	Epstein-Barr virus infection	154	525	0.5060718	1	ko05169
11	Lysosome	153	428	0.002032993	0.02954015	ko04142
12	Tight junction	151	496	0.2975467	0.9812218	ko04530
13	*Vibrio cholerae* infection	147	476	0.2327261	0.9074972	ko05110
14	Endocytosis	147	518	0.6919742	1	ko04144
15	Purine metabolism	146	563	0.9666811	1	ko00230
16	Spliceosome	145	564	0.9748267	1	ko03040
17	Phagosome	144	398	0.001588723	0.02658463	ko04145
18	Influenza A	138	372	0.000632526	0.01221261	ko05164
19	*Salmonella* infection	138	384	0.002612639	0.03122726	ko05132
20	Dilated cardiomyopathy	134	458	0.5223085	1	ko05414
21	Ubiquitin mediated proteolysis	133	520	0.974373	1	ko04120
22	Pyrimidine metabolism	132	411	0.1113596	0.5375242	ko00240
23	Neuroactive ligand-receptor interaction	130	552	0.9990565	1	ko04080
24	Transcriptional misregulation in cancer	129	391	0.05897496	0.3289492	ko05202
25	Hypertrophic cardiomyopathy (HCM)	127	450	0.707703	1	ko05410
26	Herpes simplex infection	125	420	0.4317154	1	ko05168
27	Protein digestion and absorption	123	345	0.00577145	0.05571669	ko04974
28	mRNA surveillance pathway	121	429	0.7066355	1	ko03015
29	Cell cycle	119	389	0.3004444	0.9812218	ko04110
30	Viral myocarditis	117	306	0.000431914	0.009034205	ko05416
31	Protein processing in endoplasmic reticulum	116	425	0.8323293	1	ko04141
32	Amyotrophic lateral sclerosis (ALS)	113	340	0.06100307	0.3328646	ko05014
33	Pancreatic secretion	112	318	0.01209728	0.09488804	ko04972
34	Tuberculosis	112	338	0.06655709	0.3480381	ko05152
35	Cardiac muscle contraction	102	331	0.2864477	0.9812218	ko04260
36	Pathogenic *Escherichia coli* infection	101	279	0.007097571	0.06598112	ko05130

37	Bile secretion	100	530	1	1	ko04976
38	Ribosome biogenesis in eukaryotes	99	258	0.000992773	0.01779901	ko03008
39	Insulin signaling pathway	99	373	0.8916962	1	ko04910
40	MAPK signaling pathway	94	345	0.8148587	1	ko04010
41	Lysine degradation	87	286	0.3561956	1	ko00310
42	Gastric acid secretion	87	309	0.6895969	1	ko04971
43	Calcium signaling pathway	87	401	0.999786	1	ko04020
44	Oocyte meiosis	86	231	0.005382862	0.05404393	ko04114
45	Antigen processing and presentation	84	147	1.57887E-12	3.96297E-10	ko04612
46	Wnt signaling pathway	83	271	0.333296	1	ko04310
47	Progesterone-mediated oocyte maturation	82	336	0.9807981	1	ko04914
48	ABC transporters	82	400	0.9999781	1	ko02010
49	Alzheimer's disease	81	378	0.9998027	1	ko05010
50	Chemokine signaling pathway	74	293	0.9457363	1	ko04062
51	Glycerophospholipid metabolism	73	226	0.1757492	0.7493622	ko00564
52	Glutamatergic synapse	72	228	0.2422403	0.9074972	ko04724
53	Glycerolipid metabolism	71	191	0.0110623	0.08956895	ko00561
54	Bacterial invasion of epithelial cells	70	231	0.3897729	1	ko05100
55	ECM-receptor interaction	70	299	0.9907252	1	ko04512
56	Adherens junction	69	278	0.9583455	1	ko04520
57	Arginine and proline metabolism	68	138	5.63557E-07	1.76816E-05	ko00330
58	Salivary secretion	68	255	0.8395474	1	ko04970
59	Fc gamma R-mediated phagocytosis	64	244	0.8702617	1	ko04666
60	RNA polymerase	64	275	0.9896876	1	ko03020
61	Peroxisome	63	223	0.6573699	1	ko04146
62	Vasopressin-regulated water reabsorption	61	316	0.9999833	1	ko04962
63	RNA degradation	60	167	0.03690707	0.2315919	ko03018
64	Dorso-ventral axis formation	60	187	0.2204041	0.9069087	ko04320
65	Base excision repair	59	97	1.02846E-10	8.60482E-09	ko03410
66	Fat digestion and absorption	59	146	0.002533229	0.03122726	ko04975
67	Neurotrophin signaling pathway	59	200	0.4995561	1	ko04722
68	Parkinson's disease	59	269	0.9974754	1	ko05012
69	Alcoholism	57	175	0.1893419	0.7920803	ko05034
70	Phosphatidylinositol signaling system	56	198	0.6477833	1	ko04070
71	Nucleotide excision repair	54	87	2.13593E-10	1.3403E-08	ko03420
72	Shigellosis	54	185	0.538874	1	ko05131
73	Axon guidance	54	253	0.9983745	1	ko04360
74	Dopaminergic synapse	53	214	0.939474	1	ko04728
75	Leukocyte transendothelial migration	53	234	0.9909752	1	ko04670
76	Basal transcription factors	52	154	0.1279129	0.6034077	ko03022

77	Rheumatoid arthritis	51	123	0.002545299	0.03122726	ko05323
78	DNA replication	50	76	4.60592E-11	5.78043E-09	ko03030
79	Toxoplasmosis	50	229	0.995725	1	ko05145
80	Small cell lung cancer	49	202	0.9530911	1	ko05222
81	Olfactory transduction	48	122	0.01071757	0.08956895	ko04740
82	Measles	48	143	0.1497114	0.6832284	ko05162
83	Oxidative phosphorylation	48	272	0.999997	1	ko00190
84	Starch and sucrose metabolism	48	273	0.9999974	1	ko00500
85	Prion diseases	46	126	0.04754048	0.2734035	ko05020
86	Glutathione metabolism	46	131	0.08598168	0.4390156	ko00480
87	Gap junction	45	123	0.04792731	0.2734035	ko04540
88	Synaptic vesicle cycle	45	158	0.6173332	1	ko04721
89	GnRH signaling pathway	44	157	0.6645975	1	ko04912
90	p53 signaling pathway	43	99	0.001864989	0.02925701	ko04115
91	Complement and coagulation cascades	43	112	0.02358333	0.1599842	ko04610
92	Fanconi anemia pathway	43	137	0.3229038	1	ko03460
93	Melanogenesis	43	162	0.8029831	1	ko04916
94	Galactose metabolism	43	212	0.9989288	1	ko00052
95	ErbB signaling pathway	42	143	0.5222603	1	ko04012
96	Inositol phosphate metabolism	41	143	0.5951856	1	ko00562
97	VEGF signaling pathway	41	163	0.8955773	1	ko04370
98	T cell receptor signaling pathway	41	184	0.9869992	1	ko04660
99	Cocaine addiction	40	129	0.3642596	1	ko05030
100	Glycolysis / Gluconeogenesis	40	167	0.9480392	1	ko00010
101	Apoptosis	39	100	0.02315448	0.1599842	ko04210
102	Legionellosis	39	116	0.1761449	0.7493622	ko05134
103	Vitamin digestion and absorption	38	96	0.01931338	0.1425782	ko04977
104	Amphetamine addiction	38	144	0.8034226	1	ko05031
105	Prostate cancer	38	181	0.9955697	1	ko05215
106	N-Glycan biosynthesis	37	84	0.002791642	0.0318501	ko00510
107	Long-term potentiation	37	158	0.9591308	1	ko04720
108	Cytosolic DNA-sensing pathway	36	129	0.6664118	1	ko04623
109	Hepatitis C	36	136	0.7918323	1	ko05160
110	alpha-Linolenic acid metabolism	35	83	0.00821007	0.07359741	ko00592
111	Glioma	35	116	0.45097	1	ko05214
112	PPAR signaling pathway	35	142	0.9066669	1	ko03320
113	Amino sugar and nucleotide sugar metabolism	34	139	0.9128902	1	ko00520
114	Pentose and glucuronate interconversions	34	189	0.9998783	1	ko00040
115	Toll-like receptor signaling pathway	33	78	0.009514229	0.08234729	ko04620
116	Phototransduction - fly	33	128	0.8339045	1	ko04745

117	Morphine addiction	33	132	0.8822623	1	ko05032
118	Phototransduction	32	88	0.0909099	0.4474193	ko04744
119	Notch signaling pathway	32	99	0.2855201	0.9812218	ko04330
120	Other types of O-glycan biosynthesis	32	114	0.6464234	1	ko00514
121	Epithelial cell signaling in *Helicobacter pylori* infection	32	122	0.799164	1	ko05120
122	Renal cell carcinoma	32	134	0.9320558	1	ko05211
123	Fructose and mannose metabolism	31	102	0.4392428	1	ko00051
124	TGF-beta signaling pathway	30	110	0.7119265	1	ko04350
125	Serotonergic synapse	30	111	0.731974	1	ko04726
126	Mismatch repair	29	41	4.83328E-08	2.42631E-06	ko03430
127	Jak-STAT signaling pathway	29	90	0.3049215	0.9812218	ko04630
128	Endocrine and other factor-regulated calcium reabsorption	29	97	0.4851302	1	ko04961
129	Osteoclast differentiation	29	103	0.6356431	1	ko04380
130	Glycine, serine and threonine metabolism	29	117	0.880909	1	ko00260
131	Cytokine-cytokine receptor interaction	29	139	0.9905601	1	ko04060
132	Chronic myeloid leukemia	28	94	0.4958961	1	ko05220
133	Colorectal cancer	28	103	0.7146228	1	ko05210
134	Hedgehog signaling pathway	28	104	0.7351973	1	ko04340
135	Pyruvate metabolism	28	135	0.990613	1	ko00620
136	GABAergic synapse	28	137	0.992869	1	ko04727
137	Terpenoid backbone biosynthesis	27	56	0.002118417	0.02954015	ko00900
138	Drug metabolism - other enzymes	27	206	1	1	ko00983
139	Homologous recombination	26	55	0.003578948	0.03782088	ko03440
140	NF-kappa B signaling pathway	26	94	0.6725799	1	ko04064
141	Cholinergic synapse	26	119	0.9732396	1	ko04725
142	Fc epsilon RI signaling pathway	25	74	0.2317475	0.9074972	ko04664
143	Collecting duct acid secretion	25	75	0.2560468	0.9314166	ko04966
144	Pancreatic cancer	25	76	0.2813237	0.9812218	ko05212
145	NOD-like receptor signaling pathway	24	66	0.1298168	0.6034077	ko04621
146	Cysteine and methionine metabolism	24	90	0.7430935	1	ko00270
147	Citrate cycle (TCA cycle)	24	93	0.8017267	1	ko00020
148	Endometrial cancer	24	104	0.9362436	1	ko05213
149	Pentose phosphate pathway	24	104	0.9362436	1	ko00030
150	Retrograde endocannabinoid signaling	24	137	0.9994817	1	ko04723
151	Glycosphingolipid biosynthesis - lacto and neolacto series	23	30	1.07324E-07	4.48971E-06	ko00601
152	Hematopoietic cell lineage	23	69	0.2677122	0.9599395	ko04640
153	*Staphylococcus aureus* infection	23	82	0.6376854	1	ko05150
154	Arrhythmogenic right ventricular	23	173	0.9999998	1	ko05412

	cardiomyopathy (ARVC)					
155	Proximal tubule bicarbonate reclamation	22	54	0.04721091	0.2734035	ko04964
156	Natural killer cell mediated cytotoxicity	22	83	0.7479612	1	ko04650
157	Thyroid cancer	21	73	0.5824562	1	ko05216
158	Aminoacyl-tRNA biosynthesis	21	74	0.6111505	1	ko00970
159	B cell receptor signaling pathway	21	83	0.820358	1	ko04662
160	Type II diabetes mellitus	21	87	0.8813751	1	ko04930
161	Carbohydrate digestion and absorption	21	163	0.9999998	1	ko04973
162	Glycosaminoglycan biosynthesis - keratan sulfate	20	25	2.18834E-07	7.84675E-06	ko00533
163	Acute myeloid leukemia	20	67	0.5052118	1	ko05221
164	Pertussis	20	78	0.7958734	1	ko05133
165	Basal cell carcinoma	20	82	0.8645541	1	ko05217
166	Cell adhesion molecules (CAMs)	20	145	0.9999968	1	ko04514
167	mTOR signaling pathway	19	108	0.9982091	1	ko04150
168	Adipocytokine signaling pathway	19	109	0.998497	1	ko04920
169	Glycosaminoglycan biosynthesis - heparan sulfate	19	114	0.9993886	1	ko00534
170	Ether lipid metabolism	18	52	0.2405556	0.9074972	ko00565
171	Melanoma	18	54	0.3013493	0.9812218	ko05218
172	Arachidonic acid metabolism	18	63	0.5964686	1	ko00590
173	Non-small cell lung cancer	18	64	0.626912	1	ko05223
174	Tyrosine metabolism	18	72	0.8230491	1	ko00350
175	Long-term depression	18	78	0.911002	1	ko04730
176	Other glycan degradation	17	38	0.03084296	0.2037259	ko00511
177	Aldosterone-regulated sodium reabsorption	17	46	0.1623924	0.7278659	ko04960
178	Alanine, aspartate and glutamate metabolism	17	63	0.700414	1	ko00250
179	Tryptophan metabolism	17	85	0.9805966	1	ko00380
180	Metabolism of xenobiotics by cytochrome P450	17	134	0.9999986	1	ko00980
181	Glycosaminoglycan biosynthesis - chondroitin sulfate	16	33	0.0153423	0.1166945	ko00532
182	Renin-angiotensin system	16	46	0.251096	0.9268396	ko04614
183	Insect hormone biosynthesis	16	52	0.4583715	1	ko00981
184	Chagas disease (American trypanosomiasis)	16	76	0.9596553	1	ko05142
185	Ascorbate and aldarate metabolism	16	97	0.9988171	1	ko00053
186	Steroid hormone biosynthesis	16	110	0.99991	1	ko00140
187	Retinol metabolism	16	115	0.9999686	1	ko00830

188	Glycosphingolipid biosynthesis - globo series	15	21	7.88495E-05	0.001799202	ko00603
189	Sphingolipid metabolism	15	47	0.3984004	1	ko00600
190	beta-Alanine metabolism	15	58	0.7602694	1	ko00410
191	Glyoxylate and dicarboxylate metabolism	15	59	0.7845302	1	ko00630
192	Drug metabolism - cytochrome P450	15	130	0.9999997	1	ko00982
193	Histidine metabolism	14	37	0.1668651	0.7347919	ko00340
194	SNARE interactions in vesicular transport	14	51	0.6640486	1	ko04130
195	Bladder cancer	14	52	0.6951596	1	ko05219
196	Proteasome	14	66	0.9466184	1	ko03050
197	Fatty acid elongation	14	78	0.9924282	1	ko00062
198	D-Arginine and D-ornithine metabolism	13	15	6.42162E-06	0.000179092	ko00472
199	Glycosaminoglycan degradation	13	49	0.71409	1	ko00531
200	Biosynthesis of unsaturated fatty acids	13	52	0.795299	1	ko01040
201	Systemic lupus erythematosus	13	84	0.9989652	1	ko05322
202	Porphyrin and chlorophyll metabolism	13	103	0.999983	1	ko00860
203	MAPK signaling pathway - fly	12	49	0.8134312	1	ko04013
204	Fatty acid metabolism	12	62	0.9726496	1	ko00071
205	Ribosome	12	299	1	1	ko03010
206	Non-homologous end-joining	11	22	0.03254825	0.2094772	ko03450
207	Mucin type O-Glycan biosynthesis	11	24	0.06340415	0.3386051	ko00512
208	Nicotinate and nicotinamide metabolism	11	41	0.6909206	1	ko00760
209	Linoleic acid metabolism	11	46	0.8319261	1	ko00591
210	Primary immunodeficiency	11	72	0.9982045	1	ko05340
211	Steroid biosynthesis	10	33	0.5140926	1	ko00100
212	RIG-I-like receptor signaling pathway	10	45	0.8885247	1	ko04622
213	Butanoate metabolism	10	55	0.9791908	1	ko00650
214	Valine, leucine and isoleucine degradation	10	75	0.9996701	1	ko00280
215	Mineral absorption	10	108	1	1	ko04978
216	Pantothenate and CoA biosynthesis	9	26	0.3419295	1	ko00770
217	Folate biosynthesis	9	29	0.4871664	1	ko00790
218	Protein export	9	37	0.798477	1	ko03060
219	Circadian rhythm - fly	9	51	0.9806151	1	ko04711
220	Glycosylphosphatidylinositol(GPI)-anchor biosynthesis	9	55	0.991188	1	ko00563
221	Propanoate metabolism	9	58	0.9952516	1	ko00640
222	Nicotine addiction	9	69	0.9995828	1	ko05033
223	D-Glutamine and D-glutamate metabolism	8	11	0.003616339	0.03782088	ko00471
224	Taste transduction	8	36	0.8692817	1	ko04742
225	Fatty acid biosynthesis	8	52	0.9937102	1	ko00061
226	Maturity onset diabetes of the young	7	23	0.5298984	1	ko04950

227	Selenocompound metabolism	7	27	0.7170174	1	ko00450
228	Phenylalanine metabolism	7	38	0.9562257	1	ko00360
229	Riboflavin metabolism	7	40	0.9705265	1	ko00740
230	Leishmaniasis	7	45	0.9896462	1	ko05140
231	Biotin metabolism	6	9	0.02237752	0.1599842	ko00780
232	Glycosphingolipid biosynthesis - ganglio series	6	10	0.04222275	0.2584856	ko00604
233	Malaria	6	47	0.9978786	1	ko05144
234	Taurine and hypotaurine metabolism	5	13	0.3241839	1	ko00430
235	Autoimmune thyroid disease	5	19	0.6943567	1	ko05320
236	Type I diabetes mellitus	5	20	0.7405452	1	ko04940
237	One carbon pool by folate	5	23	0.8478598	1	ko00670
238	Circadian rhythm - mammal	5	37	0.9929338	1	ko04710
239	Vitamin B6 metabolism	4	15	0.6818147	1	ko00750
240	Cyanoamino acid metabolism	4	17	0.7797323	1	ko00460
241	Synthesis and degradation of ketone bodies	3	11	0.6685278	1	ko00072
242	Regulation of autophagy	3	19	0.9472264	1	ko04140
243	Sulfur metabolism	3	22	0.97572	1	ko00920
244	Ubiquinone and other terpenoid-quinone biosynthesis	3	22	0.97572	1	ko00130
245	Butirosin and neomycin biosynthesis	2	16	0.9702047	1	ko00524
246	Intestinal immune network for IgA production	1	1	0.2928036	0.9812218	ko04672
247	Asthma	1	3	0.646335	1	ko05310
248	Lipoic acid metabolism	1	4	0.7499056	1	ko00785
249	African trypanosomiasis	1	13	0.9889518	1	ko05143
250	Valine, leucine and isoleucine biosynthesis	1	15	0.9944777	1	ko00290
251	Sulfur relay system	1	18	0.9980489	1	ko04122

Table S10a: Gene set enrichment analysis comparing *A. suturalis* female adults and eggs

Category	Subcategory	Total
Biological process	cellular process	4014
	single-organism process	3122
	metabolic process	3037
	biological regulation	1936
	regulation of biological process	1774
	multicellular organismal process	1733
	developmental process	1684
	response to stimulus	1447
	cellular component organization or biogenesis	1420
	localization	1070
	signaling	945
	establishment of localization	900
	reproduction	661
	reproductive process	594
	positive regulation of biological process	511
	negative regulation of biological process	485
	locomotion	427
	multi-organism process	281
	immune system process	271
	growth	240
	biological adhesion	120
	rhythmic process	48
	cell killing	3
Cellular component	cell	3021
	cell part	3021
	organelle	2224
	organelle part	1337
	macromolecular complex	1233
	membrane	1019
	membrane part	590
	membrane-enclosed lumen	476
	extracellular region	133
	synapse	117
	cell junction	114
	synapse part	85
	extracellular region part	81

	extracellular matrix	41
	extracellular matrix part	22
	nucleoid	1
Molecular function	binding	2980
	catalytic activity	2817
	transporter activity	342
	structural molecule activity	267
	molecular transducer activity	155
	nucleic acid binding transcription factor activity	153
	enzyme regulator activity	151
	receptor activity	127
	protein binding transcription factor activity	73
	electron carrier activity	39
	antioxidant activity	22
	translation regulator activity	10
	channel regulator activity	6
	receptor regulator activity	3
	metallochaperone activity	2
	morphogen activity	1
	protein tag	1

Table S10b: Gene set pathway analysis comparing *A. suturalis* female adults (AFA) and eggs

	Pathway	AFA/eggs (21206)	All Unigenes (44442)	P-value	Q-value	Pathway ID
**1**	Metabolic pathways	1244	2737	1	1	ko01100
**2**	Regulation of actin cytoskeleton	400	804	0.890269	1	ko04810
**3**	RNA transport	382	703	0.04847981	0.320712589	ko03013
**4**	Pathways in cancer	343	613	0.009229115	0.102733267	ko05200
**5**	Spliceosome	319	564	0.005431824	0.065231629	ko03040
**6**	Focal adhesion	319	744	0.9999985	1	ko04510
**7**	Epstein-Barr virus infection	275	525	0.3066377	0.988906583	ko05169
**8**	Amoebiasis	274	617	0.9997415	1	ko05146
**9**	Tight junction	267	496	0.1264724	0.5530488	ko04530
**10**	Endocytosis	250	518	0.9191326	1	ko04144
**11**	Vascular smooth muscle contraction	250	557	0.9989278	1	ko04270
**12**	Hypertrophic cardiomyopathy (HCM)	249	450	0.04184491	0.294967123	ko05410
**13**	Dilated cardiomyopathy	249	458	0.09234408	0.46352662	ko05414
**14**	HTLV-I infection	242	434	0.03027918	0.260400948	ko05166
**15**	Purine metabolism	241	563	0.9999784	1	ko00230
**16**	Ubiquitin mediated proteolysis	235	520	0.9976184	1	ko04120
**17**	Huntington's disease	230	632	1	1	ko05016
**18**	mRNA surveillance pathway	225	429	0.3175389	1	ko03015
**19**	*Vibrio cholerae* infection	224	476	0.9696473	1	ko05110
**20**	Herpes simplex infection	218	420	0.403856	1	ko05168
**21**	Transcriptional misregulation in cancer	212	391	0.1236278	0.549930559	ko05202
**22**	Protein processing in endoplasmic reticulum	212	425	0.7250178	1	ko04141
**23**	Pyrimidine metabolism	210	411	0.5368	1	ko00240
**24**	Lysosome	209	428	0.8506398	1	ko04142
**25**	Phagosome	206	398	0.4304486	1	ko04145
**26**	*Salmonella* infection	204	384	0.238415	0.842617397	ko05132
**27**	Influenza A	201	372	0.1463561	0.599363076	ko05164
**28**	Tuberculosis	198	338	0.003560156	0.051028903	ko05152
**29**	Ribosome	194	299	1.01247E-06	0.000261216	ko03010
**30**	Cardiac muscle contraction	183	331	0.0739772	0.41122061	ko04260
**31**	Cell cycle	183	389	0.956095	1	ko04110
**32**	Alzheimer's disease	181	378	0.9120957	1	ko05010
**33**	MAPK signaling pathway	177	345	0.505887	1	ko04010
**34**	Viral myocarditis	172	306	0.04344477	0.294967123	ko05416
**35**	Insulin signaling pathway	171	373	0.983872	1	ko04910
**36**	Neuroactive ligand-receptor interaction	159	552	1	1	ko04080
**37**	Wnt signaling pathway	156	271	0.01998194	0.198282328	ko04310

**38**	Chemokine signaling pathway	154	293	0.3406592	1	ko04062
**39**	Pathogenic *Escherichia coli* infection	149	279	0.2475099	0.851434056	ko05130
**40**	Bile secretion	149	530	1	1	ko04976
**41**	Ribosome biogenesis in eukaryotes	147	258	0.03519788	0.292937195	ko03008
**42**	Adherens junction	142	278	0.5401035	1	ko04520
**43**	Calcium signaling pathway	142	401	1	1	ko04020
**44**	Axon guidance	140	253	0.1033234	0.476025664	ko04360
**45**	Pancreatic secretion	139	318	0.9970355	1	ko04972
**46**	Protein digestion and absorption	138	345	0.99999	1	ko04974
**47**	Bacterial invasion of epithelial cells	136	231	0.01107523	0.114296374	ko05100
**48**	ECM-receptor interaction	133	299	0.9918213	1	ko04512
**49**	Lysine degradation	131	286	0.9711711	1	ko00310
**50**	Leukocyte transendothelial migration	130	234	0.1008632	0.476025664	ko04670
**51**	Neurotrophin signaling pathway	128	200	0.000162633	0.005994191	ko04722
**52**	Parkinson's disease	126	269	0.9333598	1	ko05012
**53**	Progesterone-mediated oocyte maturation	126	336	0.9999999	1	ko04914
**54**	Oocyte meiosis	123	231	0.2876977	0.951615469	ko04114
**55**	Fc gamma R-mediated phagocytosis	123	244	0.6227011	1	ko04666
**56**	Amyotrophic lateral sclerosis (ALS)	123	340	1	1	ko05014
**57**	Gastric acid secretion	122	309	0.9999879	1	ko04971
**58**	RNA polymerase	120	275	0.9951886	1	ko03020
**59**	RNA degradation	113	167	1.09523E-05	0.000706425	ko03018
**60**	Salivary secretion	111	255	0.9943122	1	ko04970
**61**	Oxidative phosphorylation	111	272	0.9997842	1	ko00190
**62**	Glycerophospholipid metabolism	109	226	0.8326646	1	ko00564
**63**	Shigellosis	108	185	0.0291676	0.259491062	ko05131
**64**	Phosphatidylinositol signaling system	105	198	0.3276095	1	ko04070
**65**	Toxoplasmosis	104	229	0.966228	1	ko05145
**66**	Dorso-ventral axis formation	101	187	0.2421553	0.844271181	ko04320
**67**	Prostate cancer	100	181	0.1536689	0.607042424	ko05215
**68**	Dopaminergic synapse	100	214	0.9168058	1	ko04728
**69**	Peroxisome	99	223	0.9826798	1	ko04146
**70**	Antigen processing and presentation	97	147	0.000193387	0.006236728	ko04612
**71**	Small cell lung cancer	96	202	0.8687709	1	ko05222
**72**	Starch and sucrose metabolism	96	273	1	1	ko00500
**73**	T cell receptor signaling pathway	95	184	0.4827276	1	ko04660
**74**	Glycerolipid metabolism	94	191	0.7336677	1	ko00561
**75**	Measles	93	143	0.000550286	0.011831138	ko05162
**76**	Melanogenesis	91	162	0.1162769	0.526305968	ko04916
**77**	ABC transporters	91	400	1	1	ko02010

**78**	Alcoholism	89	175	0.5663147	1	ko05034
**79**	Cytosolic DNA-sensing pathway	85	129	0.000502064	0.01177569	ko04623
**80**	ErbB signaling pathway	85	143	0.02871768	0.259491062	ko04012
**81**	GnRH signaling pathway	82	157	0.4290468	1	ko04912
**82**	Drug metabolism - other enzymes	82	206	0.99962	1	ko00983
**83**	Glutamatergic synapse	82	228	0.9999988	1	ko04724
**84**	Vasopressin-regulated water reabsorption	81	316	1	1	ko04962
**85**	Fanconi anemia pathway	80	137	0.05391577	0.338717143	ko03460
**86**	Inositol phosphate metabolism	80	143	0.1455008	0.599363076	ko00562
**87**	Renal cell carcinoma	79	134	0.04276078	0.294967123	ko05211
**88**	Hepatitis C	76	136	0.1560575	0.607042424	ko05160
**89**	VEGF signaling pathway	74	163	0.9413705	1	ko04370
**90**	Glycolysis / Gluconeogenesis	74	167	0.969022	1	ko00010
**91**	Basal transcription factors	73	154	0.8477674	1	ko03022
**92**	Pentose and glucuronate interconversions	72	189	0.9998955	1	ko00040
**93**	Arrhythmogenic right ventricular cardiomyopathy (ARVC)	71	173	0.9971623	1	ko05412
**94**	Galactose metabolism	71	212	1	1	ko00052
**95**	Metabolism of xenobiotics by cytochrome P450	69	134	0.5075243	1	ko00980
**96**	Colorectal cancer	66	103	0.005562387	0.065231629	ko05210
**97**	TGF-beta signaling pathway	66	110	0.03912323	0.294658925	ko04350
**98**	Drug metabolism - cytochrome P450	65	130	0.6415297	1	ko00982
**99**	Pyruvate metabolism	65	135	0.7875112	1	ko00620
**100**	Amino sugar and nucleotide sugar metabolism	65	139	0.8718186	1	ko00520
**101**	Chronic myeloid leukemia	64	94	0.000653131	0.012962138	ko05220
**102**	Nucleotide excision repair	63	87	4.28253E-05	0.002209788	ko03420
**103**	Base excision repair	63	97	0.004236358	0.054649018	ko03410
**104**	Endometrial cancer	61	104	0.07650616	0.41122061	ko05213
**105**	Glutathione metabolism	61	131	0.8753669	1	ko00480
**106**	Fat digestion and absorption	61	146	0.991137	1	ko04975
**107**	Long-term potentiation	61	158	0.9994612	1	ko04720
**108**	Steroid hormone biosynthesis	60	110	0.2715804	0.909970691	ko00140
**109**	Jak-STAT signaling pathway	59	90	0.004078757	0.054649018	ko04630
**110**	Glioma	59	116	0.5660348	1	ko05214
**111**	GABAergic synapse	59	137	0.9771608	1	ko04727
**112**	Notch signaling pathway	58	99	0.08450516	0.444945536	ko04330
**113**	Epithelial cell signaling in *Helicobacter pylori* infection	58	122	0.8163295	1	ko05120
**114**	Rheumatoid arthritis	58	123	0.8390222	1	ko05323
**115**	Carbohydrate digestion and absorption	58	163	0.99998	1	ko04973

**116**	PPAR signaling pathway	57	142	0.99689	1	ko03320
**117**	p53 signaling pathway	56	99	0.1659262	0.629543524	ko04115
**118**	DNA replication	55	76	0.000133207	0.005727901	ko03030
**119**	NF-kappa B signaling pathway	55	94	0.09342397	0.46352662	ko04064
**120**	Osteoclast differentiation	55	103	0.3638463	1	ko04380
**121**	Legionellosis	55	116	0.8188378	1	ko05134
**122**	Amphetamine addiction	55	144	0.9993775	1	ko05031
**123**	Cell adhesion molecules (CAMs)	55	145	0.9995228	1	ko04514
**124**	Retinol metabolism	54	115	0.842834	1	ko00830
**125**	Phototransduction - fly	54	128	0.9837132	1	ko04745
**126**	Toll-like receptor signaling pathway	53	78	0.001979185	0.034041982	ko04620
**127**	Basal cell carcinoma	53	82	0.009556583	0.102733267	ko05217
**128**	Glycine, serine and threonine metabolism	53	117	0.9151669	1	ko00260
**129**	Arginine and proline metabolism	53	138	0.9990738	1	ko00330
**130**	Pancreatic cancer	52	76	0.001715352	0.031611487	ko05212
**131**	Hedgehog signaling pathway	52	104	0.6345051	1	ko04340
**132**	mTOR signaling pathway	52	108	0.768043	1	ko04150
**133**	Olfactory transduction	52	122	0.976898	1	ko04740
**134**	Retrograde endocannabinoid signaling	52	137	0.9993346	1	ko04723
**135**	Fructose and mannose metabolism	51	102	0.6339702	1	ko00051
**136**	Fc epsilon RI signaling pathway	50	74	0.003146067	0.047746193	ko04664
**137**	Other types of O-glycan biosynthesis	50	114	0.9522303	1	ko00514
**138**	Gap junction	50	123	0.9927125	1	ko04540
**139**	Prion diseases	50	126	0.9964318	1	ko05020
**140**	Vitamin digestion and absorption	49	96	0.553175	1	ko04977
**141**	Natural killer cell mediated cytotoxicity	48	83	0.1352199	0.571913675	ko04650
**142**	B cell receptor signaling pathway	48	83	0.1352199	0.571913675	ko04662
**143**	Cysteine and methionine metabolism	48	90	0.3822503	1	ko00270
**144**	Cholinergic synapse	48	119	0.993335	1	ko04725
**145**	Chagas disease (American trypanosomiasis)	47	76	0.03997311	0.294658925	ko05142
**146**	Adipocytokine signaling pathway	47	109	0.9632326	1	ko04920
**147**	Complement and coagulation cascades	47	112	0.9802	1	ko04610
**148**	Morphine addiction	47	132	0.999894	1	ko05032
**149**	Pertussis	46	78	0.1029956	0.476025664	ko05133
**150**	Fatty acid elongation	46	78	0.1029956	0.476025664	ko00062
**151**	Ascorbate and aldarate metabolism	46	97	0.8016482	1	ko00053
**152**	Synaptic vesicle cycle	46	158	1	1	ko04721
**153**	Homologous recombination	45	55	2.30347E-06	0.000297147	ko03440
**154**	Type II diabetes mellitus	44	87	0.588855	1	ko04930
**155**	Phototransduction	44	88	0.6302946	1	ko04744

**156**	Mineral absorption	44	108	0.988721	1	ko04978
**157**	Pentose phosphate pathway	43	104	0.982864	1	ko00030
**158**	Thyroid cancer	41	73	0.2319498	0.83115345	ko05216
**159**	Porphyrin and chlorophyll metabolism	41	103	0.992318	1	ko00860
**160**	Aminoacyl-tRNA biosynthesis	40	74	0.3538864	1	ko00970
**161**	Apoptosis	40	100	0.990652	1	ko04210
**162**	Non-small cell lung cancer	39	64	0.07504388	0.41122061	ko05223
**163**	Citrate cycle (TCA cycle)	38	93	0.9824333	1	ko00020
**164**	Serotonergic synapse	38	111	0.999895	1	ko04726
**165**	Cocaine addiction	38	129	0.9999998	1	ko05030
**166**	Cytokine-cytokine receptor interaction	38	139	1	1	ko04060
**167**	MAPK signaling pathway - fly	37	49	0.000423867	0.010935774	ko04013
**168**	Proteasome	36	66	0.3370792	1	ko03050
**169**	Hematopoietic cell lineage	36	69	0.4836975	1	ko04640
**170**	NOD-like receptor signaling pathway	35	66	0.4309896	1	ko04621
**171**	Acute myeloid leukemia	35	67	0.4811232	1	ko05221
**172**	Tyrosine metabolism	35	72	0.7116486	1	ko00350
**173**	alpha-Linolenic acid metabolism	35	83	0.9608872	1	ko00592
**174**	Insect hormone biosynthesis	34	52	0.02731465	0.259491062	ko00981
**175**	Glycosylphosphatidylinositol(GPI)-anchor biosynthesis	34	55	0.07402611	0.41122061	ko00563
**176**	Valine, leucine and isoleucine degradation	34	75	0.871565	1	ko00280
**177**	*Staphylococcus aureus* infection	34	82	0.9699804	1	ko05150
**178**	Biosynthesis of unsaturated fatty acids	33	52	0.05060797	0.326421407	ko01040
**179**	N-Glycan biosynthesis	33	84	0.9893987	1	ko00510
**180**	Tryptophan metabolism	33	85	0.9918743	1	ko00380
**181**	Mismatch repair	32	41	0.000370858	0.010631266	ko03430
**182**	Arachidonic acid metabolism	32	63	0.5758219	1	ko00590
**183**	Butanoate metabolism	31	55	0.2639435	0.896018724	ko00650
**184**	Long-term depression	31	78	0.9840779	1	ko04730
**185**	Systemic lupus erythematosus	31	84	0.9970016	1	ko05322
**186**	Linoleic acid metabolism	30	46	0.03868515	0.294658925	ko00591
**187**	Melanoma	29	54	0.4085636	1	ko05218
**188**	Endocrine and other factor-regulated calcium reabsorption	29	97	0.9999936	1	ko04961
**189**	Glycosaminoglycan biosynthesis - heparan sulfate	29	114	1	1	ko00534
**190**	Leishmaniasis	28	45	0.0909752	0.46352662	ko05140
**191**	Sphingolipid metabolism	28	47	0.1576428	0.607042424	ko00600
**192**	beta-Alanine metabolism	28	58	0.718204	1	ko00410
**193**	Aldosterone-regulated sodium reabsorption	27	46	0.1920933	0.70800102	ko04960

**194**	Bladder cancer	27	52	0.5140265	1	ko05219
**195**	Riboflavin metabolism	26	40	0.05514	0.338717143	ko00740
**196**	SNARE interactions in vesicular transport	26	51	0.5683405	1	ko04130
**197**	Ether lipid metabolism	26	52	0.6226545	1	ko00565
**198**	Propanoate metabolism	26	58	0.865125	1	ko00640
**199**	Fatty acid metabolism	26	62	0.9441173	1	ko00071
**200**	Glyoxylate and dicarboxylate metabolism	25	59	0.931868	1	ko00630
**201**	Alanine, aspartate and glutamate metabolism	25	63	0.9751317	1	ko00250
**202**	Primary immunodeficiency	25	72	0.9983538	1	ko05340
**203**	Nicotinate and nicotinamide metabolism	24	41	0.2166454	0.787246665	ko00760
**204**	Circadian rhythm - fly	24	51	0.7680063	1	ko04711
**205**	Non-homologous end-joining	21	22	8.73472E-06	0.000706425	ko03450
**206**	Circadian rhythm - mammal	21	37	0.3047952	0.988906583	ko04710
**207**	RIG-I-like receptor signaling pathway	21	45	0.7757602	1	ko04622
**208**	Renin-angiotensin system	21	46	0.8161747	1	ko04614
**209**	Glycosaminoglycan degradation	21	49	0.9054627	1	ko00531
**210**	Fatty acid biosynthesis	21	52	0.955808	1	ko00061
**211**	Terpenoid backbone biosynthesis	19	56	0.9969541	1	ko00900
**212**	Nicotine addiction	18	69	0.9999941	1	ko05033
**213**	Protein export	17	37	0.7893231	1	ko03060
**214**	Phenylalanine metabolism	17	38	0.8314382	1	ko00360
**215**	Malaria	17	47	0.98681	1	ko05144
**216**	Collecting duct acid secretion	17	75	1	1	ko04966
**217**	One carbon pool by folate	16	23	0.05877895	0.3526737	ko00670
**218**	Folate biosynthesis	15	29	0.551805	1	ko00790
**219**	Regulation of autophagy	14	19	0.03989358	0.294658925	ko04140
**220**	Sulfur metabolism	14	22	0.1701039	0.63604067	ko00920
**221**	Pantothenate and CoA biosynthesis	14	26	0.4712136	1	ko00770
**222**	Steroid biosynthesis	14	33	0.881786	1	ko00100
**223**	Glycosaminoglycan biosynthesis - chondroitin sulfate	14	33	0.881786	1	ko00532
**224**	Histidine metabolism	14	37	0.963909	1	ko00340
**225**	Other glycan degradation	14	38	0.9739763	1	ko00511
**226**	Proximal tubule bicarbonate reclamation	13	54	0.999988	1	ko04964
**227**	Ubiquinone and other terpenoid-quinone biosynthesis	12	22	0.4606415	1	ko00130
**228**	Mucin type O-Glycan biosynthesis	12	24	0.6263452	1	ko00512
**229**	Vitamin B6 metabolism	11	15	0.07094447	0.41122061	ko00750
**230**	Maturity onset diabetes of the young	11	23	0.7026723	1	ko04950
**231**	Biotin metabolism	9	9	0.002412544	0.038902272	ko00780
**232**	Taurine and hypotaurine metabolism	9	13	0.1530632	0.607042424	ko00430

**233**	Sulfur relay system	9	18	0.6322396	1	ko04122
**234**	Type I diabetes mellitus	9	20	0.7816582	1	ko04940
**235**	Glycosphingolipid biosynthesis - lacto and neolacto series	9	30	0.9944306	1	ko00601
**236**	Glycosphingolipid biosynthesis - globo series	8	21	0.9227767	1	ko00603
**237**	Glycosaminoglycan biosynthesis - keratan sulfate	8	25	0.9838937	1	ko00533
**238**	Taste transduction	8	36	0.999911	1	ko04742
**239**	African trypanosomiasis	7	13	0.5350937	1	ko05143
**240**	Cyanoamino acid metabolism	7	17	0.8576429	1	ko00460
**241**	Selenocompound metabolism	7	27	0.9979906	1	ko00450
**242**	Caffeine metabolism	7	36	0.9999811	1	ko00232
**243**	Valine, leucine and isoleucine biosynthesis	6	15	0.8701203	1	ko00290
**244**	Autoimmune thyroid disease	6	19	0.9750291	1	ko05320
**245**	Synthesis and degradation of ketone bodies	5	11	0.7520042	1	ko00072
**246**	Butirosin and neomycin biosynthesis	5	16	0.9689864	1	ko00524
**247**	Glycosphingolipid biosynthesis - ganglio series	4	10	0.8471325	1	ko00604
**248**	D-Arginine and D-ornithine metabolism	4	15	0.986079	1	ko00472
**249**	Lipoic acid metabolism	3	4	0.3306581	1	ko00785
**250**	Lysine biosynthesis	3	4	0.3306581	1	ko00300
**251**	Asthma	2	3	0.5179625	1	ko05310
**252**	Thiamine metabolism	2	3	0.5179625	1	ko00730
**253**	Phenylalanine, tyrosine and tryptophan biosynthesis	2	7	0.9450228	1	ko00400
**254**	Allograft rejection	1	1	0.5119767	1	ko05330
**255**	Graft-versus-host disease	1	1	0.5119767	1	ko05332
**256**	Intestinal immune network for IgA production	1	1	0.5119767	1	ko04672
**257**	Primary bile acid biosynthesis	1	8	0.9967874	1	ko00120
**258**	D-Glutamine and D-glutamate metabolism	1	11	0.9996271	1	ko00471

Table S11: Primers sets for qRT-PCR analyses of differentially expressed genes in *A. suturalis*

Genes		Primer sequences
p450	Forward primer	5'TCAGACGACGCCTGCAGCAAGA3'
	Reverse primer	5'TCGCCGAAGTAAGGCTGTAGAG3'
vitellogenin	Forward primer	5'TTACTATACCGAAGCAATCGTA3'
	Reverse primer	5'GATGGGTTGGTCTTCGCATAGG3'
flightin	Forward primer	5'CCCAGCCACCGAAGTGACG3'
	Reverse primer	5'GTGGTAGTAGTTGTACATGTAG3'
Neurofibromin	Forward primer	5'AGTGACCCATCAAAGAATCGAA3'
	Reverse primer	5'GTGCTTGGACGGCAGGGTCAGT3'
thioredoxin	Forward primer	5'TCTATTTCGTTGGCTGCGTCG3'
	Reverse primer	5'TTGGTGTTCTTGAGAAAATC3'
KLHL	Forward primer	5'TCCAATGAAGCCTGTAA3'
	Reverse primer	5'GGGTCATAAACTTCTGCTGTA3'
MHC	Forward primer	5'TCAGAAAGCAAGATGCCGACGT'
	Reverse primer	5'ACCTGTTCACCGTTCTTG3'
titin	Forward primer	5'GGCCATCGCTTCTCTTTCGC3'
	Reverse primer	5'CTATTATGTTGTCACTGCCGTC3'
laminin	Forward primer	5'GAAGTAAAGAGACTGGCGCCAG3'
	Reverse primer	5'AATTAAGTCAAGTCAAGTAAGT'
18S rRNA	Forward primer	5'CCACAGAGCCAGAACTATGGAT3'
	Reverse primer	5'TAATTCCGAAGGTGCCTACTAAGC3'
